# Estimating the Quality of Reprogrammed Cells Using ES Cell Differentiation Expression Patterns

**DOI:** 10.1371/journal.pone.0015336

**Published:** 2011-01-11

**Authors:** Bo Zhang, Beibei Chen, Tao Wu, Yuliang Tan, Shuang Qiu, Zhenyu Xuan, Xiaopeng Zhu, Runsheng Chen

**Affiliations:** 1 National Laboratory of Biomacromolecules, Institute of Biophysics, Chinese Academy of Sciences, Beijing, People's Republic of China; 2 Graduate University of Chinese Academy of Sciences, Beijing, People's Republic of China; 3 Department of Molecular and Cell Biology, Center for Systems Biology, University of Texas at Dallas, Richardson, Texas, United States of America; University of Bristol, United Kingdom

## Abstract

Somatic cells can be reprogrammed to a pluripotent state by over-expression of defined factors, and pluripotency has been confirmed by the tetraploid complementation assay. However, especially in human cells, estimating the quality of Induced Pluripotent Stem Cell(iPSC) is still difficult. Here, we present a novel supervised method for the assessment of the quality of iPSCs by estimating the gene expression profile using a 2-D “Differentiation-index coordinate”, which consists of two “developing lines” that reflects the directions of ES cell differentiation and the changes of cell states during differentiation. By applying a novel liner model to describe the differentiation trajectory, we transformed the ES cell differentiation time-course expression profiles to linear “developing lines”; and use these lines to construct the 2-D “Differentiation-index coordinate” of mouse and human. We compared the published gene expression profiles of iPSCs, ESCs and fibroblasts in mouse and human “Differentiation-index coordinate”. Moreover, we defined the *Distance index* to indicate the qualities of iPS cells, which based on the projection distance of iPSCs-ESCs and iPSCs-fibroblasts. The results indicated that the “Differentiation-index coordinate” can distinguish differentiation states of the different cells types. Furthermore, by applying this method to the analysis of expression profiles in the tetraploid complementation assay, we showed that the *Distance index* which reflected spatial distributions correlated the pluripotency of iPSCs. We also analyzed the significantly changed gene sets of “developing lines”. The results suggest that the method presented here is not only suitable for the estimation of the quality of iPS cells based on expression profiles, but also is a new approach to analyze time-resolved experimental data.

## Introduction

As a promising technology, induced pluripotent stem cells (iPSCs) are playing important roles in many fields, including personal therapy and scientific research. Both human and mouse fibroblast cells have been reprogrammed to a pluripotent cell state by the over-expression of several transcription factors (TF) that appear in embryonic stem cells [1]–[6]. In addition, many kinds of somatic cells, including adipose cell, neurons and so on, have also been reprogrammed to a pluripotent cell state by rapidly developing iPS technologies [1], [2], [3], [4], [5], [6].

Similar to ES cells, the pluripotency of iPS cells evoke expectation and enthusiasm. Different experimental and theoretical approaches have been applied to estimate the similarity between iPSCs and ESCs. Among these approaches, microarray technology and clustering analysis are widely used to detect expression patterns during the reprogramming process. Mark [7] compared the expression profiles of iPSCs and ESCs by clustering analysis and concluded that iPSCs could be considered as a subtype of pluripotent cells. By comparing the percentage of differentially expressed genes between iPSCs and ESCs, Zhumur [8] estimated several iPS cell lines originated from different cell types. However, it is still difficult to accurately measure the quality of iPSCs based on molecular characteristics and to estimate the pluripotency of ES cells and iPSCs.

Here, we introduce a new supervised method to estimate the quality of iPSCs based on gene expression profiles from the perspective of ES cell differentiation ability. Inspired by the description of differentiation trajectories in a high-dimensional state space, time-resolved expression profiles of ESC differentiation processes were transformed into linear scales, which were named “developing lines” and represent differentiation directions and the changes in gene expression over time. Here, these developing lines were used to measure the transcription profiles of iPSCs and undifferentiated ESCs. If the iPSCs are similar to ESCs, they should have the similar projection positions on these “developing lines”. Moreover, we defined the concept of the *Distance index*, which reflects a spatial distance, to measure the similarity of each sample to ESCs. This method not only provided an estimation of the quality of iPSCs based on similarities between iPSCs-ESCs and iPSCs-fibroblasts at the transcriptional level but was also a novel approach for the analysis of time-resolved experimental data.

## Results

### Distinct descriptions of similarities among cell types in mouse and human “Differentiation-index Coordinates”

**Figure 1:**
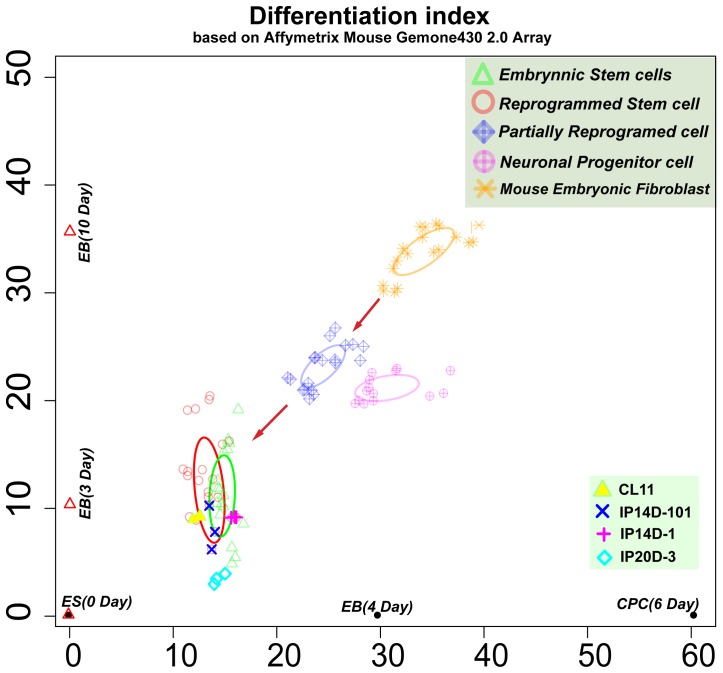
Estimates of Different Cell Types in the Mouse Differentiation Coordinate. The X-axis is the cardiac precursor cell developing line; the Y-axis is the pancreatic islets developing line. CL11, IP14D-101, IP14D-1 and IP20D-3 are contained in Dataset GSE15925. The red arrows indicate the movement of cell state changes. Ellipses were generated by the mean values and standard variances.

To construct the mouse “Differentiation-index Coordinate”, dataset GSE10970, which contains a series of time-resolved differentiation gene expression profiles for ESC-derived cardiac precursor cells (CPCs), and dataset GSE3653, which contains a series of time-resolved differentiation gene expression profiles for ESC-derived pancreatic islets (PIs), were transformed to a CPC developing line and PI developing line as described in the Methods. All collected mouse expression profiles of iPSCs, ESCs, partly reprogrammed cells, neuronal progenitor cells and fibroblast cells were estimated by this two-dimensional surface coordinate (Figure 1).

The mouse Differentiation-index coordinate accurately distinguished between the distributions of different cell types and clearly showed the cell state changes during the reprogramming process. The mouse embryonic fibroblast cells were located the largest distance from the ESCs. The partially reprogrammed cells were located in the middle between the ESC region and fibroblast region. iPSCs were located close to the ESC region and partly overlapped with it. Meanwhile, neuronal progenitor cells were located more closely to the ESC region than the fibroblast cells, which indicated a greater similarity between neuronal progenitor cells and ESCs. Recent studies have indicated that compared with the four factors (Oct3/4, Sox2, c-Myc, and Klf4) required to induce fibroblast cells, neuronal progenitor cells could be induced into a pluripotent state by only Oct4 (Pou5f1) expression[9], [10]. These results suggested that the distance between ESCs and other cell types in the Differentiation-index coordinate not only reflected their cell state and similarities but also partly indicated the difficulty of inducing them to a pluripotent state.

We also included dataset GSE16925, which was generated with the tetraploid complementation assay [11] and showed whether the mouse iPSCs have the ability to develop into an embryo and mature mice. Based on the large number of ESC expression profiles, it was obvious that IP14 (including three replicates of IP14D-1 and IP14D-101) was very close to the ESC region, while the location of IP20 (including three replicates of IP20D-3) was far away from the ESC region. In their study, by using blastocyst injection, 624 IP14D-1 reprogrammed cells generated 22 live pups (3.5%), 181 IP14D-101 reprogrammed cells generated 4 live pups (2.2%), and 204 IP20D-3 reprogrammed cells did not generate any live pups. The “Differentiation index coordinate” clearly described the relationships among all cell types from this study: IP14D-101 was more similar to CL11 ESCs than to IP14D-1 (a shorter distance between IP14D-101 and CL11), which was confirmed by the hierarchical clustering analysis [11]. Moreover, due to the deviation of the CL11 ESCs, hierarchical clustering analysis may not reflect the qualities of iPSCs in this case.

Then, we selected the GSE9940 dataset of human ES cell-derived neural rosette differentiation expression profiles to generate a human neuronal developing line. The GSE8884 dataset of human ES cell-derived blast cell differentiation expression profiles was used to generate the second axis, a blast cell developing line (blast cells generate both hematopoietic and endothelial progenies upon transfer to the appropriate conditions). Thus, the two-dimensional human “Differentiation-index coordinate” was obtained.

**Figure 2:**
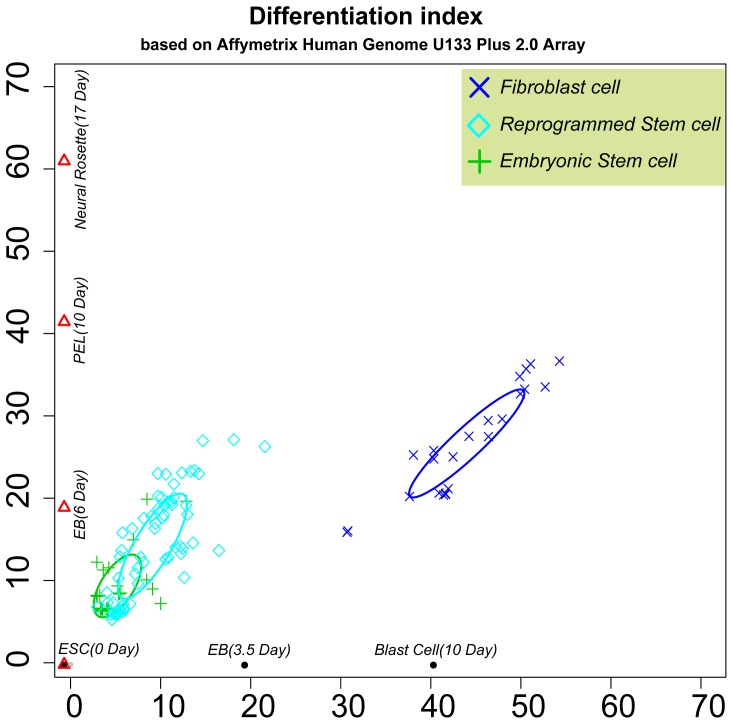
Estimates of Different Cell Types in the Human Differentiation Coordinate. The X-axis is the blast cell developing line; the Y-axis is the neuronal developing line. Ellipses were generated by the mean values and standard variances.

All collected expression profiles of human iPSCs, ESCs and fibroblast cells were estimated using the human Differentiation-index coordinate (Figure 2). The result showed that only part of the iPSCs overlapped with the ESCs, and many iPSCs were still located far away from the ESCs. In addition, we found that the blast cell developing line was more effective and had more resolving power to distinguish iPSCs than the neuronal developing line. For estimating the resolution power of the neuronal developing line for different cell states, we used dataset GSE9921, which contained gene expression profiles of human ESCs and neural rosettes (Figure S1), as a test. The result showed that the neuronal developing line distinguished between the neurons and ESCs. These results indicated that the developing line has the best resolution for distinguishing between corresponding cell types.

### Distance-index calculation

The “Differentiation-index coordinate” could be used to generate a clear and intuitive estimation of pluripotency for different kinds of cells, especially iPSCs. In the Differentiation-index coordinate, the distinct projection positions of iPSCs directly demonstrate their similarities to ESCs in different development directions at the transcriptional level. For estimating the similarities of iPSCs accurately, we defined the *Distance index (Di)*, which reflected the distance ratio of iPS cells to ES cells and fibroblast cells. A smaller *Distance index* (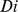) demonstrates a higher similarity of the iPSCs to the center of the ES cells and dissimilarity to the fibroblast cells.

We calculated the *Distance indices (Di)* for all collected ESCs (Tables S1, S2) and iPSCs (Tables S3, S4) for human and mouse. Based on the *Di* of human and mouse ESCs, we set the mean value of all ESC Distance indices as the threshold, which was 0.09384 in human and 0.11024 in mouse. This threshold reflects the dispersion of the ESC transcriptome. Eighty percent of human ESCs (24 of 30) and 60% (12 of 20) of mouse ESCs were under the threshold.

We compared the three strains of iPSCs used in the tetraploid complementation assay [11]. Interestingly, the *Distance indices* of iPSCs reflected their distinct abilities to generate live pups (Table 1). All three repeats of the IP20D-3 strain iPS cells had larger *Distance indices* than the threshold value (0.11024). However, the three replicates of IP14D-101 had larger variances than the other two cell lines.

**Table 1:** *Distance-index* of Dataset of Traploid complementation assay (GSE16925).

Dataset	Sample description	*Distance-index* *	Blastocysts#	Live pups#
GSM424481	IP14D-1-rep1	0.07858	624	22(3.5%)
GSM424482	IP14D-1-rep2	0.074245		
GSM424483	IP14D-1-rep3	0.070892		
GSM424484	IP14D-101-rep1	0.103452	181	4(2.2%)
GSM424485	**IP14D-101-rep2**	**0.146979**		
GSM424486	IP14D-101-rep3	0.048819		
GSM424487	**IP20D-3-rep1**	**0.222602**	204	0
GSM424488	**IP20D-3-rep2**	**0.211654**		
GSM424489	**IP20D-3-rep3**	**0.203322**		

*In mouse Differentiation coordinate, the threshold of ES cells is 0.11024, the Bolded items have a bigger Distance-index and be determined to “not good” iPS cells.

# These data are cited from [11].

### Analysis of significant changed genes

The methods presented here are not limited to the estimation of the similarities between different cell types; they could also be applied to the analysis of time-resolved experimental data. When calculating the developing lines, we also defined a weight for each gene to represent the expression change, and then calculated P and FDR values based on distribution of weights. With a P-value cut-off of <0.01 and an FDR of <0.1, we selected the most significantly changed genes in four different ESC differentiation processes. For assessing the functions of these significantly changed genes, we performed GO analysis using the DAVID bioinformatics resource [12]. The results (GO annotation: Tables S5, S6, S7, S8, S9, S10, S11, S12; genes list: Tables S13, S14, S15, S16, S17, S18, S19, S20) indicated that all clustered genes were involved in many morphogenetic processes of different tissues, including stem cell maintenance, prostate gland morphogenesis, neuron projection morphogenesis, gland development and so on. Moreover, some significantly regulated genes were also involved in developmental pathways, such as the retinoic acid and platelet-derived growth factor receptor signaling pathways.

Furthermore, we compared the significantly changed genes between the two experiments and generated a list of “common genes” in both human and mouse (Tables S21, S22, S23, S24). These lists contained the “common” up-regulated and down-regulated genes in the two differentiation processes with different directions and may provide some information about ESC differentiation. After comparing the lists, we found that only two genes appeared in the common lists of both human and mouse genes. POU5F1 (OCT4), which acts as an important factor in the induced pluripotency process, was the only significantly down-regulated gene in the four ESC differentiation processes. However, the NANOG gene was only in the common list for humans, and SOX2 did not appear in the common list. Correspondingly, TTR, a protein transports vitamin A (retinol) and a hormone called thyroxine throughout the body, was the only significantly up-regulated gene in the four experiments. Another important gene in the common list was GUCY1A3, which is a GTP cyclase that generates the second messenger cGMP. These significantly up-regulated common genes suggest that the initiation of cell-cell communication is crucial during ESC differentiation.

**Figure 3:**
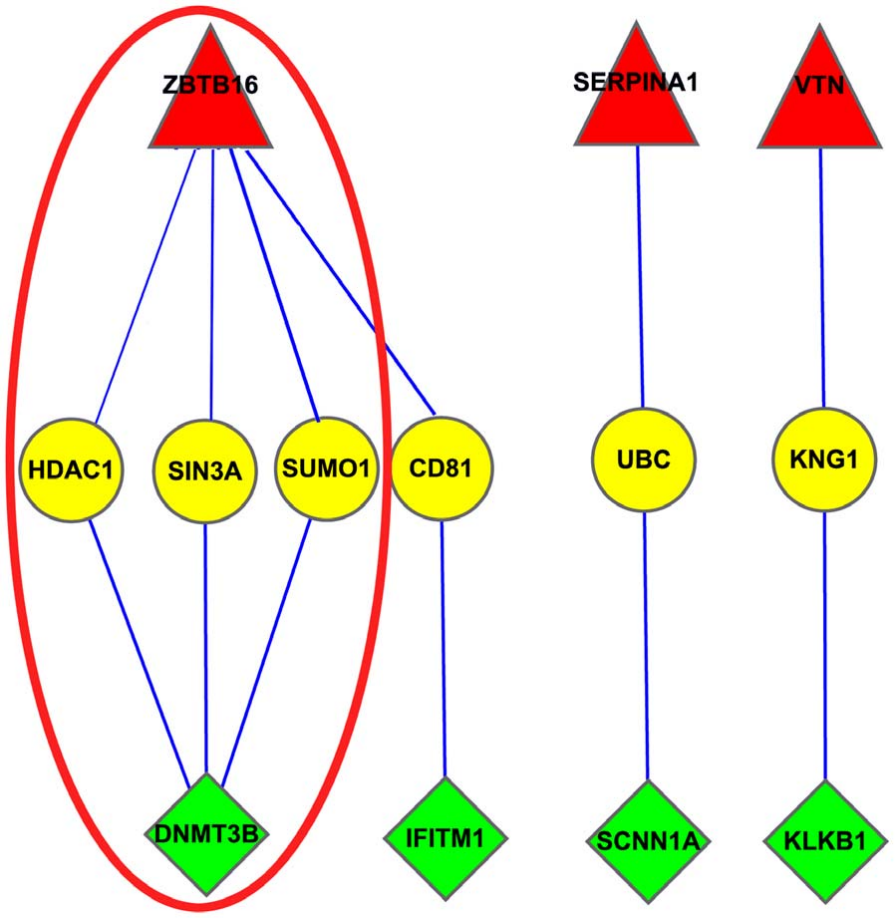
The Seesaw module that appeared in two Human ESC differentiation processes. Red: significantly up-regulated genes; Yellow: insignificantly changed genes; Green: significantly down-regulated genes. Red ellipse: the epigenetic regulation seesaw module.

**Figure 4:**
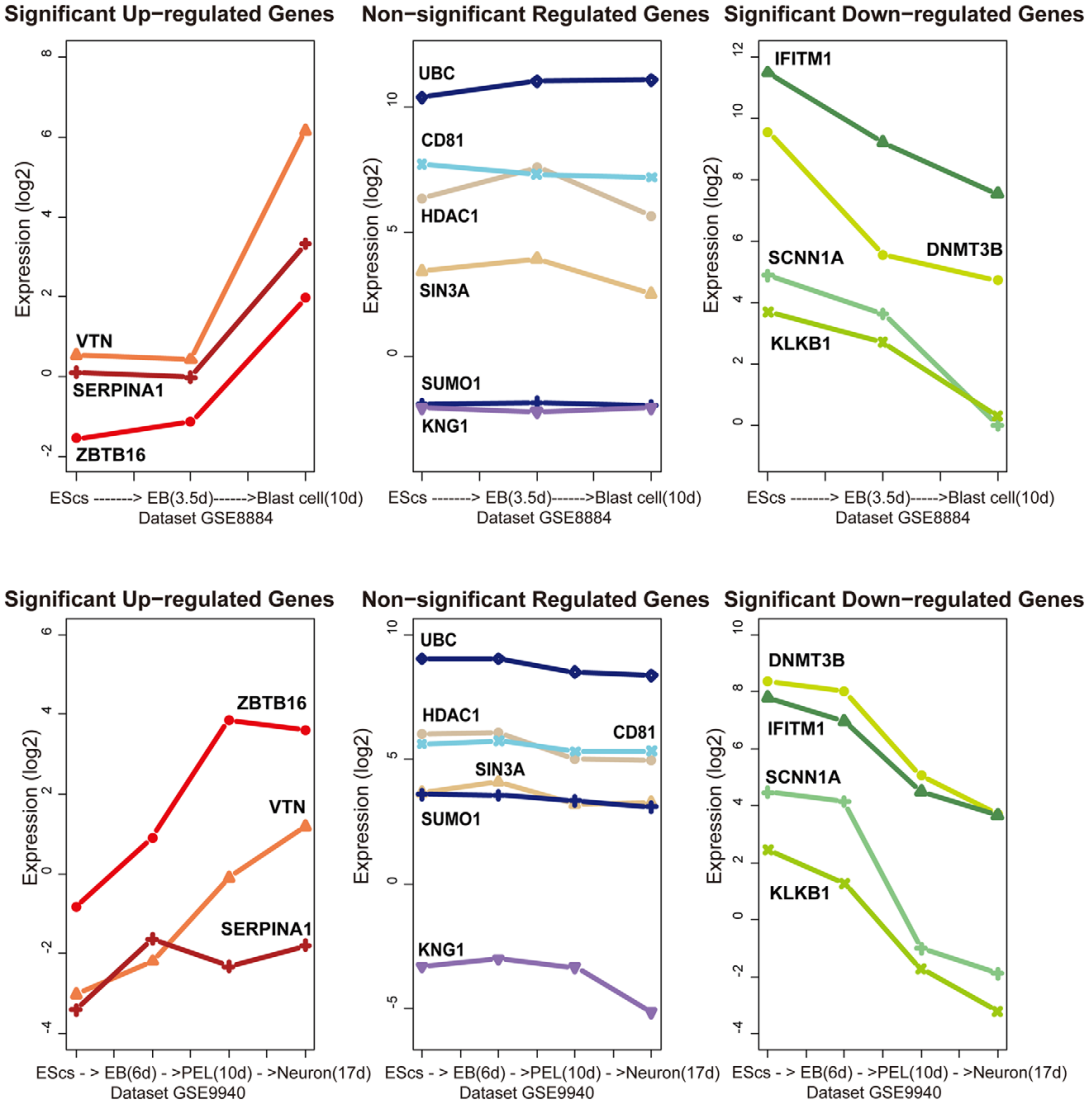
The expression patterns of genes that appeared in the Seesaw modules.

To better understand the relationship and function of these genes, we searched for their protein binding partners using protein-protein interaction data (BioGrid, Version 3.0.66). Interestingly, we found a new model that consisted of three components: down-regulated genes, up-regulated genes, and insignificantly changed genes and named it the “Seesaw module” (Figure 3A). The expression of the genes that appeared in the “Seesaw module” directly described the dynamic changes (Figure 4). The genes that appeared in the seesaw modules have been reported to be involved in developmental processes; for example, in the ZBTB16-CD81-IFITM1 module of membrane proteins, CD81 may play an important role in the regulation of lymphoma cell growth [13] and acts as the receptor for some viruses [14], and IFITM1 has been implicated in the control of cell growth [15]. Some proteinases and their inhibitors were also found in the Seesaw modules, such as SERPINA1, VTN, KNG1, and KLKB1. These genes may be involved in apoptosis or some tissue morphogenetic processes.

## Discussion

The induced pluripotent stem (iPS) cell technology is an enormously promising approach for personal therapy and scientific research. However, how the cell state alteration process happens from terminal differentiation to pluripotency is unclear. The similarities and differences in the transcriptomes of iPSCs and ES cells have been estimated [7], [8], while other properties of iPSCs are also different compared with ESCs, such as the genome methylation state [16], [17], microRNA profiling[18], histone modification, proteomic profiles [19], and so on. It is still a challenge to find an accurate and easy method to estimate the pluripotency of iPSC candidates based on these cellular properties.

The value of iPSCs is their pluripotency. From this perspective, pluripotency should be a gold standard for estimating the quality of iPSCs [20]. The tetraploid complementation assay, with is the most strict standard, has been successfully performed on mouse cells [11], [21]. Our results suggested that genome-wide expression patterns could partly reflect the pluripotency of mouse cells. The *Distance index* of dataset GSE16925 indicated that low quality iPS cells (IP20D-3) distinctly have bigger *Di* then the high quality iPS cells (IP14D-1), and this disparity is also clearly reflected by the success of live pups. We believe that the *Distance index*, as a more accurate and reasonable measurement, have the potential to become an easy standard to estimate the quality of iPSCs at molecular level.

The similarity defined by hierarchical clustering method severely depends on the mathematical characteristics of expression profiles. The system error of ES cells expression profiles would affect the clustering results. In our model, the “developing lines” generated by time-ordered linear model have distinct biological meaning: such lines are projection of ES cells differentiation trajectories. Meanwhile, the calculation of *Distance index* by this supervised method is based on a large number of expression profiles from different laboratories, and these existing datasets give our method greater robustness and accuracy. Such characteristics enable us to compare expression profiles of different sources more easily. Moreover, this method gives a simple and direct description of different cell state distributions. The dynamic changes in cell states induced by reprogramming were also clearly indicated by the “Differentiation-index coordinate”. These dynamic changes of cell states would help us to understand more about the movement trajectories of the ES cells differentiation and the reprogramming process of somatic cells.

As shown, the time-order linear model was also a novel method to analyze time-resolved experimental data. This method generated lists of the significantly up/down regulated genes during the time-resolved experiment. Based on the Protein-protein interaction network and significantly changed genes during human ES cell differentiation, we identified some interesting “seesaw” modules. One of these modules directly regulates the epigenetic changes that occur during the ESC differentiation process: ZBTB16-(HADC1, SIN3A, SUMO1)-DNMT3B. DNMT3B encodes a DNA methyltransferase that is thought to function in genome DNA de novo methylation. HADC1 encodes a histone deacetylase that is responsible for the deacetylation of lysine residues on the N-terminal tails of the core histones (H2A, H2B, H3, and H4). By SUMO1 modification, DNMT3B modulates its interaction with HDAC1 to repress the transcription of target genes [22]. ZBTB16 associates with SIN3A and HDAC1 in vitro and in vivo, and this co-repressor complex down-regulates the expression levels of target genes [23]. Down-regulation of DNMT3B in this epigenetic “seesaw module” indicates that genome methylation is lost during ESC differentiation. Recently, the human genome DNA methylation map was published at single base resolution. Compared with the undifferentiated H1 human embryonic stem cell line, the methylation level of the genome of the fetal lung fibroblast cell line IMR-90 was less than 25%, which is about 1.7*10e7 sites [16]. In particular, most of the mCHG and mCHH modifications were absent from IMR-90 cells; considering the significant down-regulation of the DNMT3B gene, this evidence suggests that DNMT3B may play an important role in the reduction in methylation during development. Moreover, the stable expression of “linker genes” indicates that DNA methylation could provide location information for gene regulation, and histone acetylation and deacetylation might directly control the transcription of target genes.

In this work, we constructed developing lines of some differentiation fates determinations in mouse and human. Theoretically, the developing line had the best resolving power for estimating the cell state of the corresponding cells, i.e, using fibroblast developing line to estimate iPSCs originated from fibroblasts, using the neuronal developing line to estimate iPSCs originated from neurons. However, for the limited number of successful ESC differentiation events in vitro, it is still difficult for us to construct developing lines that represent all directions of every ESC differentiation process. Here, we used two developing lines to estimate the pluripotency of iPSCs and other cells, and the promising results encouraged us to improve this method. Although the resolution powers of the different developing lines still need to assess, we expect that these “Differentiation-index coordinates” could reflect all of cell fates, fit all expression data, or even distinguish all cell types accurately in the future.

The approach presented here could also contribute to the construction of a “Cell type coordinate”, which would reflect the relationship between different cell types and describe the cell type-specific differences at different levels (including RNA expression, protein expression, epigenetic modification, etc.). Recently, the fibroblast cell have been directly reprogrammed to functional neurons and Cardiomyocytes [24], [25]. Such evidence lights a new approach to reprogramming cell fates. We hope to construct a “Cell type coordinate” in the future, which could show the difference among functional cells at the transcriptional level, how TFs contribute the differentiation in developmental processes, and which TF could be used to induce the transformation different functional cells. Furthermore, investigation of differentiation and improvements in reprogramming technology will help us improve the methods for qualified iPSCs selection for scientific research and clinical applications.

## Materials and Methods

### Preparation of gene expression profiles

The gene expression data were obtained from the largest expression database, Gene Expression Omnibus (GEO, http://www.ncbi.nlm.nih.gov/geo/). To construct linear scales that represent the changes in gene expression over differentiation time, we analyzed two datasets that contained the expression profiles of human ESCs differentiating into neural rosettes and blast cells (Table 2).

**Table 2:** The dataset were used to generate human ESCs differentiation developing-lines.

Dataset	Tissue	Experiment type	Publication/Experimenter
GSE9940	ESCs	ESCs in vitro differentiation to neuron rosettes	[**28**]
GSE8884	ESCs	ESCs in vitro differentiation to blast cells	[**29**]

All the gene expression dataset are published on GEO (Gene Expression Omnibus). All the dataset are based on Affymetrix Human Genome U133 Plus 2.0 Chip (GEO platform: GPL570).

To estimate iPS cell pluripotency on a large scale, we collected the expression profiles of iPS cells and ESCs based on the Affymetrix Human Genome U133 Plus 2.0 chip (GEO platform: GPL570) if possible (before Oct. 2009). All of the expression profile datasets of human iPSCs and ESCs are listed in Table 3.

**Table 3:** The datasets were used to estimate relationship between human iPSCs and human ESCs.

Dataset	Experiment samples	Samples Numbers	Publication/Experimenter
GSE12390	Human iPS and ESCs	**21**	[**30**]
GSE12583	Human iPS and ESCs	**9**	[**31**]
GSE13828	Human iPS and ESCs	**10**	[**2**]
GSE14711	Human iPS and ESCs	**11**	[**5**]
GSE15148	Human iPS and ESCs	**28**	[**32**]
GSE16093	Human iPS and ESCs	**5**	[**33**]
GSE16654	Human iPS and ESCs	**36**	[**7**]
GSE9832	Human iPS and ESCs	**16**	[**34**]
GSE9865	Human iPS and ESCs	**13**	[**35**]

All the gene expression dataset are published on GEO (Gene Expression Omnibus). All the dataset are based on Affymetrix Human Genome U133 Plus 2.0 Chip (GEO platform: GPL570).

We also constructed mouse ES cell differentiation developing lines to estimate the relationship between mouse iPSCs and mouse ESCs. All of the expression profiles were based on the Affymetrix Mouse Genome 430 2.0 chip (GEO platform: GPL1226). Two datasets containing the expression profiles of mouse ES cells differentiating to cardiac precursor cells and pancreatic islets were used to generate two differentiation developing lines (Table 4).

**Table 4:** The dataset were used to generate mouse ESCs differentiation developing-lines.

Dataset	Target Tissue	Experiment type	Publication/Experimenter
GSE10970	Cardiac precursors cells	ESCs Differentiation time-course	[**36**]
GSE3653	Pancreatic islets	ESCs Differentiation time-course	[**37**]

All the gene expression dataset are published on GEO (Gene Expression Omnibus). All the dataset are based on Affymetrix Mouse Genome 430 2.0 Chip (GEO platform: GPL1226).

As described above, we collected the expression profiles of mouse iPS cells and mouse ESCs where possible (before Oct. 2009) and analyzed these expression profiles as described in the method (Table 5).

**Table 5:** The datasets were used to estimate relationship between mouse iPSCs and mouse ESCs.

Dataset	Experiment samples	Numbers of Samples	Publication/Experimenter
GSE10806	Mouse iPS and ESCs	**11**	[**38**]
GSE10871	Mouse iPS and ESCs	**32**	[**39**]
GSE12499	Mouse iPS and ESCs	**10**	[**10**]
GSE14012	Mouse iPS and ESCs	**24**	[**40**]
GSE16925	Mouse iPS and ESCs	**15**	[**11**]
GSE8024	Mouse iPS and ESCs	**8**	[**41**]
GSE8128	Mouse iPS and ESCs	**9**	[**42**]

All the gene expression dataset are published on GEO (Gene Expression Omnibus). All the dataset are based on Affymetrix Mouse Genome 430 2.0 Chip (GEO platform: GPL1226).

Probe signal estimates were derived from the SOFT files. Each probe was treated as an independent transcript. The log-transformed values of the expression data were then median-normalized independently for each dataset.

### Construction of Developing-lines and Differentiation-index coordinate

To construct developing lines that represent ESC differentiation processes, we used a time-ordered linear model algorithm to transform time-resolved ESC's differentiation expression profiles into the Octave environment.

Inspiriting from Clustering [26], [27] and PCA method, this linear model is based on the description of the developmental trajectory as a line with a distinct direction, which represents the gene expression change over developmental time. In order to achieve such aim, tow points must be required:

1. the line could preserve the time order of the projected points
2. the line could preserve the distance ratio of joint sample points in the microarray space

In high-dimensional space(in this paper, the dimensions are determined by genes number *N*), a series of unlooped, head-to-tail joint vectors (here which represent sample change at *t* time points, and *N*>*t*) have one co-bisector. Because the angles between each vector and co-bisector are the same, after each vector is projected onto the co-bisector, the strict order and length ratio of every vector is preserved perfectly. Naturally, the co-bisector of a series of vectors has the longest length among all bisectors (Figure S2), and it can represent the moving trend of sample. The co-bisector suited our two requirements for a linear model that represents the processes of tissue development and cell differentiation.

First, we built a microarray space, in which the dimensions were determined by probes that represented transcripts on the microarray chip. Based on the same microarray platform, each expression profile had a unique position in this high-dimensional space.

*X* is a 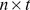 matrix, which represents the expression data for *n* genes measured at *t* time points. *X_i_* represents the expression profile at time point *i*, for all 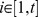. The expression score of gene 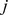 at time point 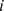 is 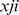.
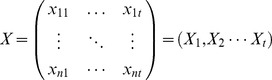
(1)


For the purpose of preserving the strict order of the projected points of 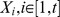 on the projected line, we first generated *(t-1)* vectors 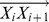, forall 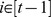, and the vectors are given by: 
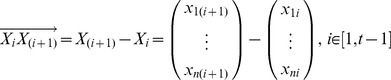
(2)


Then, we defined the co-bisector as 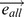. The inner product of vector 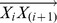
and the co-bisector 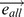 is 
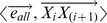
, and it should satisfy the equation below: 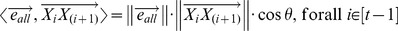
(3)


Naturally, after the points of 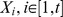 were projected onto the co-bisector of 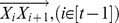, the projected points retained their order. Among all co-bisectors that could preserve the distance ratio of these sample points by projection, the co-bisector 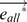
in the linear subspace determined by 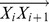, forall 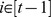, has the longest length. Thus, the optimized 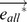 could be represented as the linear combination of 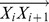, forall 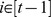:
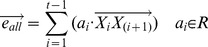
(4)


To simplify our calculations, we set 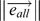 to 1:
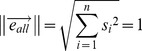
(5)


With equations (2), (3), (4), and (5), the parameters 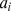 and 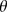 and vector 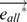
were determined.

Thus 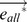
was obtained, and this vector represented the change in the expression level during differentiation from the ESC state to a terminally differentiated state. We named this vector the “developing line”.

When the expression profiles of other samples are projected onto the vector (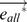), the projection position P_i_ of each sample is calculated by: 
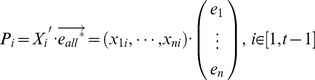
(6)


This projection position represents the relative similarity at the transcriptional level. To account for similar gene expression states, the same kinds of cells were grouped together, even though the expression data came from different laboratories.

We generated two human ESC differentiation developing lines and two mouse ESC differentiation developing lines that corresponded to four time-resolved ESC differentiation expression profiles. Then all of the collected expression data for ESCs, fibroblast cells and iPSCs were individually projected onto the developing lines. The coordinates of all projection positions were analyzed and visualized with the R software. The projection regions of ESCs, fibroblast cells and iPSCs were determined by the mean values and standard deviations of each cell type.

The microarray approach usually contains the noise which generated from experiment stage. In order to see the robustness of our time-ordered linear model, we selected mice fetal liver development time-course expression profiles to make tests. We randomly replaced the genes expression values of samples in GSE13149, and the genes number of randomly replaced was continuously increased from 1% to 20% of all genes. Then, the modified datasets were calculated to generate modified “developing lines”. Another dataset GSE6998 was projected on these modified “developing lines”. The variance of projection locations indicated the robustness of time-linear model. This model can endure 17% random replacement of total genes (Figure S3, Table S25). Such result showed this linear model has a strong robustness to noise.

### Distance index calculation

We defined the concept of a Distance index, which represents the similarity of each sample to ESCs. The centers of the ES cell projection region and the fibroblast cell projection region were set as two anchor points. The Distance index (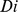) of sample A was defined as:
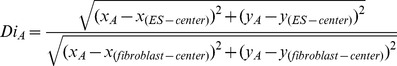
(7)


In equation (7), 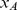 and 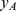 are the projection coordinates values of sample A in the “Differentiation-index coordinate”. A smaller Distance index (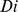) indicates that the projection of the sample is closer to the center of the ES cell projection region. The Distance index (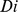) was used to generate an estimation for each iPSC. Furthermore, the distribution of the Distance index (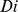) of all ESCs determine a threshold value to estimate the transcriptional similarity of iPSCs. The Distance index reflects the real distance of each cell type to the transcriptome distribution of ESCs in microarray space.

### Functional analysis

By applying multiple testing, we calculated the P-value and Benjamini-Hochberg FDR for the weight of each probe in vector 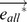. The significantly changed probes were isolated with a cut-off P-value of <0.001 and a FDR of <0.1. The probe sets were further converted to transcripts by Gene Name Bath Viewer (DAVID, http://david.abcc.ncifcrf.gov). We performed a functional analysis on the significantly changed genes on the DAVID bioinformatics resource [12].

## Supporting Information

Figure S1 **Estimates of Human ES cell and neuron rosettes in the Human Differentiation Coordinate.** The X-axis is the blast cell developing line; the Y-axis is the neuronal developing line. Ellipses were generated by the mean values and standard variances.
(PDF)

Figure S2
**Maximizing the projection of each vector on the angle-bisector.** Vector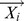 and 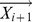 existing in a 3-D space, represent a cell departed form state A, bypassing state B, finally reached to state C. First we transform location of 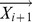 to 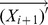, then we get the angle 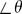, then generate one angle-bisector 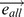, and angle
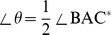
. On this angle-bisector, 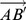 and 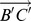 are projections of Vector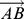 and 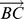. 
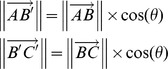
 However, in this 3-D space, there exist a plane 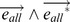 which is perpendicular to the plane ABC; each line passing point A are a angle-bisector of 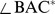, all of them meeting our requirement. Obviously, when the included angle 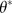 is minimized, the projection 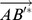 and 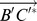 are maximized, at this time, the maximized angel-bisector is uniquely determined by intersection of plane 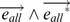 and plane ABC. When the dimensions of this space is over 3, the maximized angel-bisector is uniquely determined by intersection of all angle-bisector plane 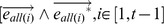. So, when the angle-bisector exists in the subspace which is determined by the parent vector, the projection length of each vector is maximized. Proof: As we know, there exists a 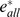


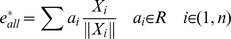


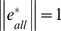


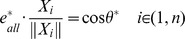
Let assume there is an 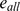 satisfies that
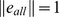


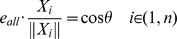


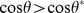
Then
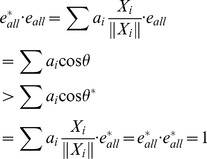
This is impossible. So there is no 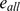 which can satisfy the condition of 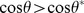. Thus, 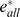 is the longest bisector.(PDF)

Figure S3 **Noise Random permutation testing to developing line (GSE13149): The Projection location of Dataset GSE6998.**
(PDF)

Table S1 **Distance-index of Human Embryonic Stem Cells.**
(PDF)

Table S2 **Distance-index of Mouse Embryonic Stem Cells.**
(PDF)

Table S3 **Distance-index of Human Induced Pluripotent Stem Cells.**
(PDF)

Table S4 **Distance-index of Mouse Induced Pluripotent Stem Cells.**
(PDF)

Table S5 **GO analysis of negative regulated genes in ES cell-derived Cardiac precursors cells Differentiation (GSE10970).**
(PDF)

Table S6 **GO analysis of positive regulated genes in ES cell-derived Cardiac precursors cells Differentiation (GSE10970).**
(PDF)

Table S7 **GO analysis of negative regulated genes in ES cell-derived Pancreatic islets cells Differentiation (GSE3653).**
(PDF)

Table S8 **GO analysis of positive regulated genes in ES cell-derived Pancreatic islets cells Differentiation (GSE3653).**
(PDF)

Table S9 **GO analysis of negative regulated genes in ES cell-derived blast cells Differentiation (GSE8884).**
(PDF)

Table S10 **GO analysis of positive regulated genes in ES cell-derived blast cells Differentiation (GSE8884).**
(PDF)

Table S11 **GO analysis of negative regulated genes in ES cell-derived neuron rosettes Differentiation (GSE9940).**
(PDF)

Table S12 **GO analysis of positive regulated genes in ES cell-derived neuron rosettes Differentiation (GSE9940).**
(PDF)

Table S13 **Positive regulated genes in ES cell-derived blast cell differentiation (GSE8884).**
(PDF)

Table S14 **Negative regulated genes in ES cell-derived blast cell differentiation (GSE8884).**
(PDF)

Table S15 **Positive regulated genes in ES cells-derived neuron rosette differentiation (GSE9940).**
(PDF)

Table S16 **Positive regulated genes in ES cells-derived neuron rosette differentiation (GSE9940).**
(PDF)

Table S17 **Negative regulated genes in ES cell-derived Cardiac precursors cells Differentiation (GSE10970).**
(PDF)

Table S18 **Positive regulated genes in ES cell-derived Cardiac precursors cells Differentiation (GSE10970).**
(PDF)

Table S19 **Negative regulated genes in ES cell-derived Pancreatic islets cells Differentiation (GSE3653).**
(PDF)

Table S20 **Positive regulated genes in ES cell-derived Pancreatic islets cells Differentiation (GSE3653).**
(PDF)

Table S21 **Significant Up-regulated Common Genes in GSE8884 and GSE9940.**
(PDF)

Table S22 **Significant Down-regulated Common Genes in GSE8884 and GSE9940.**
(PDF)

Table S23 **Significant Up-regulated Common Genes in GSE10970 and GSE3653.**
(PDF)

Table S24 **Significant Down-regulated Common Genes in GSE10970 and GSE3653.**
(PDF)

Table S25 **Noise Random permutation testing to developing line (GSE13149): The Projection location of Dataset GSE6998.**
(PDF)
