# Supplementary figures and images for: Estimating the Quality of Reprogrammed Cells Using ES Cell Differentiation Expression Patterns
Source: PLoS One. 2011 Jan 11;6(1):e15336. doi: 10.1371/journal.pone.0015336 (PMC3023460; doi:10.1371/journal.pone.0015336)

# Projection of GSE9865 in Differentiation index

based on Affymetrix Human Genome U133 Plus 2.0 Array

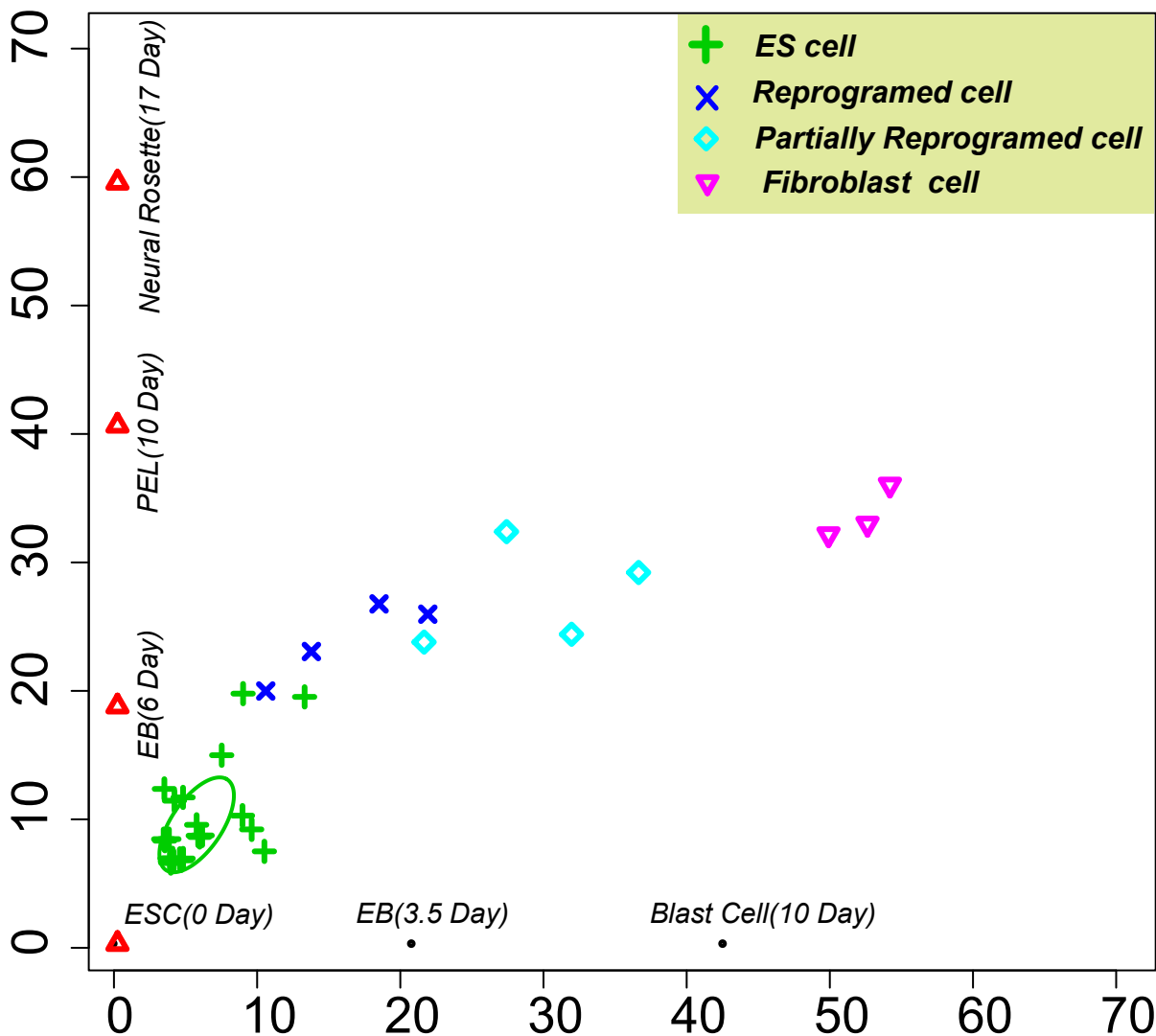

Supplement: Figure S1 — Estimates of Human ES cell and neuron rosettes in the Human Differentiation Coordinate. The X-axis is the blast cell developing line; the Y-axis is the neuronal developing line. Ellipses were generated by the mean values and standard variances. (PDF) [file pone.0015336.s001.pdf]

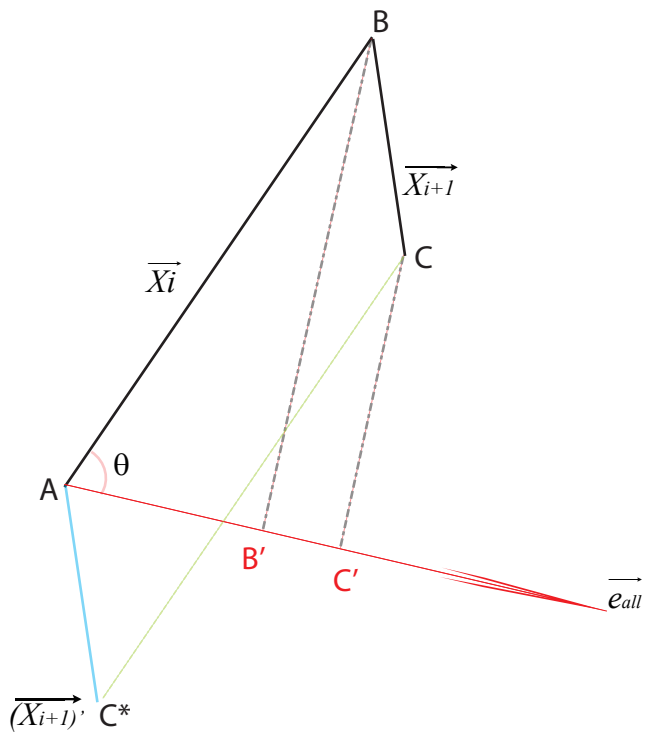

Supplement: Figure S2 — Maximizing the projection of each vector on the angle-bisector. Vector and existing in a 3-D space, represent a cell departed form state A, bypassing state B, finally reached to state C. First we transform location of to , then we get the angle , then generate one angle-bisector , and angle. On this angle-bisector, and are projections of Vector and . However, in this 3-D space, there exist a plane which is perpendicular to the plane ABC; each line passing point A are a angle-bisector of , all of them meeting our requirement. Obviously, when the included angle is minimized, the projection and are maximized, at this time, the maximized angel-bisector is uniquely determined by intersection of plane and plane ABC. When the dimensions of this space is over 3, the maximized angel-bisector is uniquely determined by intersection of all angle-bisector plane . So, when the angle-bisector exists in the subspace which is determined by the parent vector, the projection length of each vector is maximized. Proof: As we know, there exists a Let assume there is an satisfies that ThenThis is impossible. So there is no which can satisfy the condition of . Thus, is the longest bisector. (PDF) [file pone.0015336.s002.pdf]

## Noise test : GSE13149 random noise

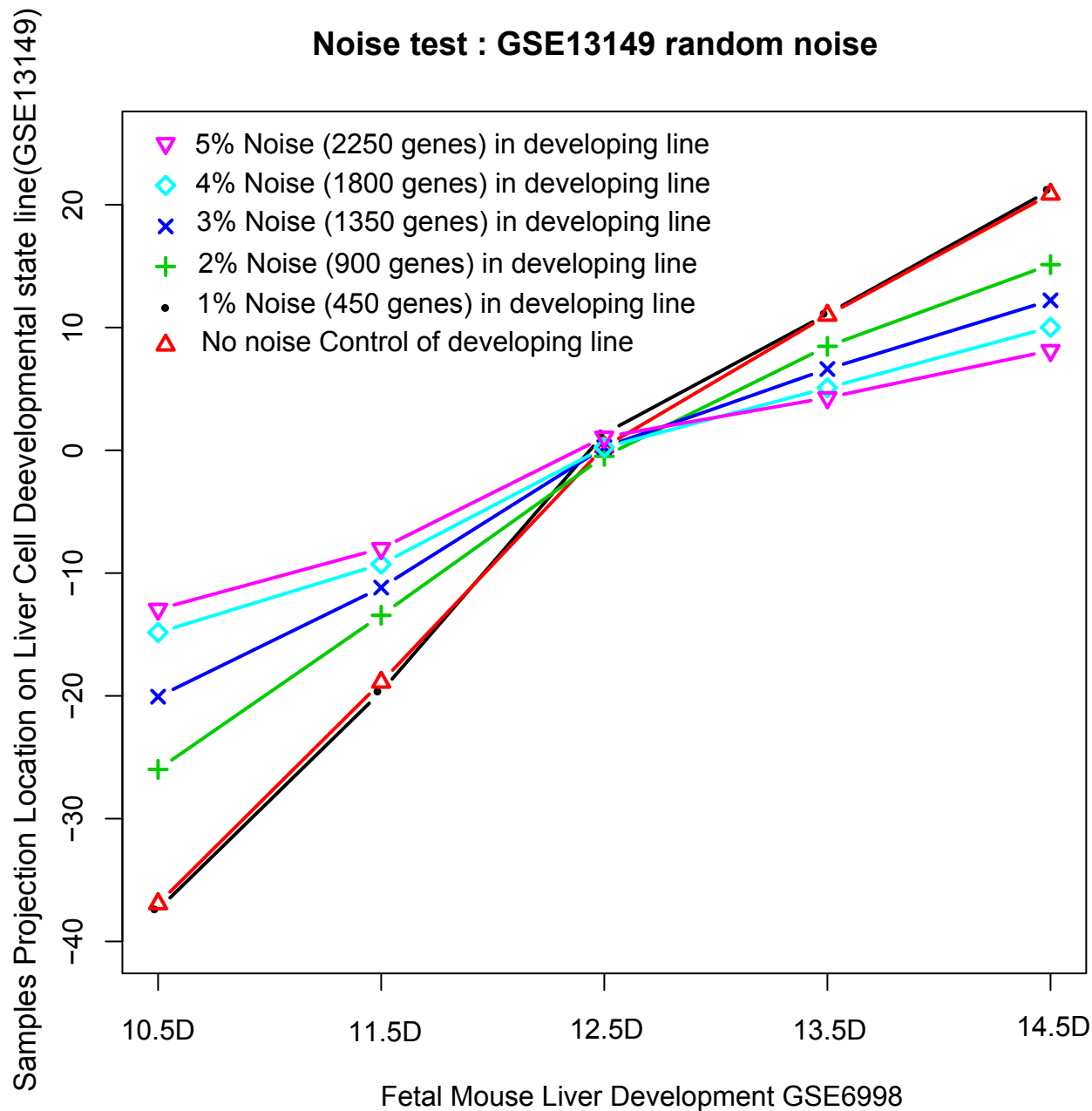

Supplement: Figure S3 — Noise Random permutation testing to developing line (GSE13149): The Projection location of Dataset GSE6998. (PDF) [file pone.0015336.s003.pdf]
