# Supplementary material for: Estimating the Quality of Reprogrammed Cells Using ES Cell Differentiation Expression Patterns
Source: PLoS One. 2011 Jan 11;6(1):e15336. doi: 10.1371/journal.pone.0015336 (PMC3023460; doi:10.1371/journal.pone.0015336)
Supplement: Table S1 — Distance-index of Human Embryonic Stem Cells. (PDF) [file pone.0015336.s004.pdf]

Table S1 Distance-index of Human Embryonic Stem Cells

Dataset	Sample description	Distance-index
GSM310860	HUES8p30 sample1	0.05197
GSM310861	HUES8p30 sample2	0.046727
GSM310862	HUES8p30 sample3	0.077712
GSM315621	Human ES2 cell line	0.095546
GSM315622	Human ES4 cell line	0.133873
GSM347921	H1L ESC	0.06259
GSM347922	H7 ESC	0.077799
GSM347923	H9 ESC	0.083617
GSM347924	H13B ESC	0.033755
GSM347925	H14A ESC	0.086481
GSM367061	hES BG01	0.282761
GSM367062	hES H9	0.131965
GSM372150	H9_undifferentiated_1	0.048682
GSM372151	H9_undifferentiated_2	0.070982
GSM372152	H9_undifferentiated_3	0.043832
GSM378811	H1LESC(setof16)	0.068928
GSM378812	H7ESC(setof16)	0.081479
GSM378813	H9ESC(setof16)	0.086956
GSM378814	H13BESC(setof16)	0.036277
GSM378815	H14AESC(setof16)	0.091547
GSM378816	H1LESC(setof12)	0.068792
GSM378817	H7ESC(setof12)	0.079929
GSM378818	H9ESC(setof12)	0.085561
GSM378819	H13BESC(setof12)	0.033891
GSM378820	H14AESC(setof12)	0.089574
GSM402708	H9 Human Embryonic Stem Cell	0.376185
GSM449729	mRNA_human ES cell line	0.075789
GSM249025	Human Embryonic Stem Cells passage49HSF1	0.053169
GSM249282	Human Embryonic Stem Cellline H9	0.042009
GSM251079	UD-Undifferentiated	0.216826
**Mean**		**0.09384**
