# Supplementary material for: Estimating the Quality of Reprogrammed Cells Using ES Cell Differentiation Expression Patterns
Source: PLoS One. 2011 Jan 11;6(1):e15336. doi: 10.1371/journal.pone.0015336 (PMC3023460; doi:10.1371/journal.pone.0015336)
Supplement: Table S2 — Distance-index of Mouse Embryonic Stem Cells. (PDF) [file pone.0015336.s005.pdf]

Table S2 Distance-index of Mouse Embryonic Stem Cells

Dataset	Sample description	Distance-index
GSM272753	Embryonic Stem cells sample 1	0.055453
GSM272836	Embryonic Stem cells sample 2	0.032067
GSM272837	Embryonic Stem cells sample 3	0.023842
GSM325390	J1 wild-type ES cells (undifferentiated), biological rep 1	0.028354
GSM325391	J1 wild-type ES cells (undifferentiated), biological rep 2	0.051045
GSM325392	CJ7 wild-type ES cells (undifferentiated), biological rep 3	0.019447
GSM325393	CJ7 wild-type ES cells (undifferentiated), biological rep 4	0.152565
GSM325394	E14tg1 wild-type ES cells (undifferentiated), biological rep 5	0.165796
GSM325395	E14tg1 wild-type ES cells (undifferentiated), biological rep 6	0.203782
GSM325396	E14tg1 wild-type ES cells (undifferentiated), biological rep 7	0.192658
GSM344757	v6.5 ES cells #1 - control	0.353605
GSM344758	v6.5 ES cells #2 - control	0.005427
GSM344759	E14 ES cells #1 - control	0.10714
GSM344760	E14 ES cells #2 - control	0.027661
GSM424475	CL11-rep1	0.087046
GSM424476	CL11-rep2	0.106445
GSM424477	CL11-rep3	0.087714
GSM198062	ES_wt_rep1	0.147219
GSM198063	ES_wt_rep2	0.172926
GSM198064	ES_wt_rep3	0.184603
**Mean**		**0.11024**
