# Supplementary material for: Estimating the Quality of Reprogrammed Cells Using ES Cell Differentiation Expression Patterns
Source: PLoS One. 2011 Jan 11;6(1):e15336. doi: 10.1371/journal.pone.0015336 (PMC3023460; doi:10.1371/journal.pone.0015336)
Supplement: Table S3 — Distance-index of Human Induced Pluripotent Stem Cells. (PDF) [file pone.0015336.s006.pdf]

Table S3 Distance-index of Human Induced Pluripotent Stem Cells

Dataset	Sample description	Distance-index
GSM310838	BJhIPS#5p9sample1	0.51789
GSM310839	BJhIPS#5p9sample2	0.511463
GSM310844	BJhIPS#5p9sample3	0.460132
GSM310845	BJhIPS#6p9sample1	0.321249
GSM310846	BJhIPS#6p9sample2	0.395952
GSM310847	BJhIPS#6p9sample3	0.314641
GSM310848	BJhIPS#8p10sample1	0.316488
GSM310849	BJhIPS#8p10sample2	0.348971
GSM310850	BJhIPS#8p10sample3	0.267209
GSM310851	BJhIPS#12p5sample1	0.361708
GSM310852	BJhIPS#12p5sample2	0.329044
GSM310853	BJhIPS#12p5sample3	0.337788
GSM310857	BJhIPS#12p6afp4#12p7sample1	0.273769
GSM310858	BJhIPS#12p6afp4#12p7sample2	0.220554
GSM310859	BJhIPS#12p6afp4#12p7sample3	0.20334
GSM315623	KiPS obtained from reprogramming human keratinocytes with 3 factors (Oct4,Sox2,Klf4)	0.282752
GSM315624	KiPS obtained from reprogramming human keratinocytes with 4 factors (Oct4,Sox2,Klf4,cmyc)1	0.20132
GSM315625	KiPS obtained from reprogramming human keratinocytes with 4 factors (Oct4,Sox2,Klf4,cmyc)2	0.377861
GSM347916	iPS SMA 3.5	0.069368
GSM347917	iPS SMA 3.6	0.050141
GSM347918	iPS SMA 4.2	0.066905
GSM367219	iPS PDB 1lox-17Puro-5	0.254893
GSM367240	iPS PDB 1lox-17Puro-10	0.298478
GSM367241	iPS PDB 1lox-21Puro-20	0.214148
GSM367242	iPS PDB 1lox-21Puro-26	0.23561
GSM367243	iPS PDB 2lox-5	0.166221
GSM367244	iPS PDB 2lox-22	0.144545
GSM367245	iPS PDB 2lox-21	0.090028
GSM367258	iPS PDB 2lox-17	0.070411
GSM372157	iPS_undifferentiated_1	0.077611
GSM372158	iPS_undifferentiated_2	0.053937
GSM372159	iPS_undifferentiated_3	0.184547
GSM378822	iPS cells from episomal vectors(Defined Factor)DF19_1(setof16)	0.088281
GSM378823	iPS cells from episomal vectors(Defined Factor)DF19_2(setof16)	0.08954
GSM378824	iPS cells from episomal vectors(Defined Factor)DF19_3(setof16)	0.092422

GSM378825	iPS cells from episomal vectors(Defined Factor)DF19_4(setof16)	0.090069
GSM378826	iPS cells from episomal vectors(Defined Factor)DF19_5(setof16)	0.089128
GSM378827	iPS cells from episomal vectors(Defined Factor)DF19_6(setof16)	0.09033
GSM378828	iPS cells from episomal vectors(Defined Factor)DF19_7(setof16)	0.070127
GSM378829	iPS cells from episomal vectors(Defined Factor)DF19_8(setof16)	0.085986
GSM378830	iPS cells from episomal vectors(Defined Factor)DF19_9(setof16)	0.078985
GSM378831	iPS cells from episomal vectors(Defined Factor)DF19_10(setof16)	0.08624
GSM378833	iPS cells from episomal vectors(Defined Factor)DF19_9(setof12)	0.07678
GSM378834	iPS cells from episomal vectors (Defined Factor sub-clone)DF19_9_11T(setof12)	0.104358
GSM378835	iPS cells from episomal vectors (Defined Factor sub-clone)DF19_9_7T(setof12)	0.079742
GSM378836	iPS cells from episomal vectors (Defined Factor sub-clone)DF6_9_12T(setof12)	0.061801
GSM378837	iPS cells from episomal vectors (Defined Factor sub-clone)DF6_9_9T(setof12)	0.065439
GSM378838	iPS cells from episomal vectors(Defined Factor)DF6_9(setof12)	0.087329
GSM402717	Human Induced Pluripotent Stem Cells by Direct Delivery of Reprogramming Proteins(Line1)	0.654583
GSM402752	Human Induced Pluripotent Stem Cells by Direct Delivery of Reprogramming Proteins(Line2)	0.408358
GSM402806	Human Induced Pluripotent Stem Cells by Direct Delivery of Reprogramming genes	0.391712
GSM417794	mRNA_Reprogrammed iPSC line1	0.212052
GSM417795	mRNA_Reprogrammed iPSC line2	0.15711
GSM417796	mRNA_Reprogrammed iPSC line18(p56)	0.093834
GSM449730	mRNA_Reprogrammed iPSC line18(p9)	0.230091
GSM248203	dH1f-iPS3-3 iPS cells	0.088834
GSM248205	dH1cf16-iPS5 iPS cells_30	0.234092
GSM248206	dH1cf16-iPS5 iPS cells_32	0.217607
GSM248207	dH1cf32-iPS2 iPS cells_10	0.167889
GSM248208	dH1cf32-iPS2 iPS cells_20	0.045547
GSM248211	MRC5-iPS2 iPS cells_2	0.064593
GSM248212	MRC5-iPS2 iPS cells_22	0.047644
GSM248215	BJ1-iPS1 iPS cells	0.008673
GSM249028	Reprogrammed cloneHips1	0.816985
GSM249095	Reprogrammed clone5hips5	0.495095
GSM249096	Reprogrammed clone2Hips2	0.318206
GSM249137	Reprogrammed clone7Hips7	1.015299
