# Supplementary material for: Estimating the Quality of Reprogrammed Cells Using ES Cell Differentiation Expression Patterns
Source: PLoS One. 2011 Jan 11;6(1):e15336. doi: 10.1371/journal.pone.0015336 (PMC3023460; doi:10.1371/journal.pone.0015336)
Supplement: Table S4 — Distance-index of Mouse Induced Pluripotent Stem Cells. (PDF) [file pone.0015336.s007.pdf]

Table S4 Distance-index of Mouse Induced Pluripotent Stem Cells

Dataset	Sample description	Distance-index
GSM272839	Induced pluripotent stem (iPS) cells (Oct4, Klf4) sample 2	0.045725
GSM272846	Induced pluripotent stem (iPS) cells (Oct4, Klf4) sample 3	0.04332
GSM272890	Induced pluripotent stem (iPS) cells (Oct4, Klf4) sample 1	0.061557
GSM279200	Induced pluripotent stem (iPS) cells (Oct4, Sox2, c-Myc, Klf4) sample 1	0.013451
GSM279201	Induced pluripotent stem (iPS) cells (Oct4, Sox2, c-Myc, Klf4) sample 2	0.105512
GSM279202	Induced pluripotent stem (iPS) cells (Oct4, Sox2, c-Myc, Klf4) sample 3	0.088152
GSM275566	B-iPS replicate 1	0.200341
GSM275567	B-iPS replicate 2	0.182356
GSM275580	Oct4-iPS cell line MCV8.1 (replicate 1)	0.110553
GSM275581	Oct4-iPS cell line MCV8.1 (replicate 2)	0.100534
GSM314038	NSCs-derived iPS cells by one-factor (Oct4) sample_1	0.124258
GSM314039	NSCs-derived iPS cells by one-factor (Oct4) sample_2	0.131441
GSM314040	NSCs-derived iPS cells by one-factor (Oct4) sample_3	0.146432
GSM344761	1D4 iPS clone #1	0.321921
GSM344762	1D4 iPS clone #2	0.319356
GSM344763	2D4 iPS clone #1	0.381719
GSM344764	2D4 iPS clone #2	0.364796
GSM424481	IP14D-1-rep1	0.07858
GSM424482	IP14D-1-rep2	0.074245
GSM424483	IP14D-1-rep3	0.070892
GSM424484	IP14D-101-rep1	0.103452
GSM424485	IP14D-101-rep2	0.146979
GSM424486	IP14D-101-rep3	0.048819
GSM424487	IP20D-3-rep1	0.222602
GSM424488	IP20D-3-rep2	0.211654
GSM424489	IP20D-3-rep3	0.203322
