# Supplementary material for: Estimating the Quality of Reprogrammed Cells Using ES Cell Differentiation Expression Patterns
Source: PLoS One. 2011 Jan 11;6(1):e15336. doi: 10.1371/journal.pone.0015336 (PMC3023460; doi:10.1371/journal.pone.0015336)
Supplement: Table S5 — GO analysis of negative regulated genes in ES cell-derived Cardiac precursors cells Differentiation (GSE10970). (PDF) [file pone.0015336.s008.pdf]

Table S5 GO analysis of negative regulated genes in ES cell-derived Cardiac precursors cells Differentiation (GSE10970)

GO number	Description	P-value	Gene
GO:0019827	stem cell maintenance	8.7E-9	Pou5f1,Sox2,Tcl1,Esrrb,Mm.212127.1,Piwil2,2410002E02Rik
GO:0007276	gamete generation	1.5E-4	D1Pas1,Mm.67875.1,Calca,Clgn,Dazl,AU019877,Morc,Nr0b1,Piwil2,Mm.50751.1,Zfp296
GO:0032526	response to retinoic acid	1.2E-3	Pou5f1,Sox2,Ebaf,2410002E02Rik
GO:0009891	positive regulation of biosynthetic process	2.4E-3	Klf5,Mm.168942.1,Pou5f1,Sox2,Dazl,Niban,Foxd3,Nr5a2,Piwil2,AI505200,Utf1,Mm.52452.1
GO:0007584	response to nutrient	2.3E-3	Pou5f1,Sox2,Mm.172133.1,Ebaf,2410002E02Rik
GO:0000122	negative regulation of transcription from RNA polymerase II promoter	1.5E-3	Mm.168942.1,Pou5f1,Sox2,Foxd3,AU019877,Nr0b1,2410002E02Rik,Crtr1-pending
GO:0001829	trophectodermal cell differentiation	5.9E-3	Pou5f1,Esrrb,Foxd3
GO:0045941	positive regulation of transcription	2.3E-2	Klf5,Mm.168942.1,Pou5f1,Sox2,Foxd3,Nr5a2,AI505200,Utf1,Mm.52452.1
GO:0051327	M phase of meiotic cell cycle	2.6E-2	Clgn,AU019877,Piwil2,Smc1l2
GO:0016049	Cell growth	3.0E-2	Pou4F2,TMP,Ebaf
