# Supplementary material for: Estimating the Quality of Reprogrammed Cells Using ES Cell Differentiation Expression Patterns
Source: PLoS One. 2011 Jan 11;6(1):e15336. doi: 10.1371/journal.pone.0015336 (PMC3023460; doi:10.1371/journal.pone.0015336)
Supplement: Table S6 — GO analysis of positive regulated genes in ES cell-derived Cardiac precursors cells Differentiation (GSE10970). (PDF) [file pone.0015336.s009.pdf]

**Table S6 GO analysis of positive regulated genes in ES cell-derived Cardiac precursors cells Differentiation (GSE10970)**

| GO number | Description | P-value | Gene |
|---|---|---|---|
| GO:0035113 | embryonic appendage morphogenesis | 4.0E-7 | BB131012,Bmp4,Dkk1,Msx1,Prrx1,Prrx2,Fbn2,Hhg1Twist,Wnt5a |
| GO:0030323 | respiratory tube development | 1.6E-6 | Bmp4,Foxa1,Lama1,Lox,Mglap,Pdgfra,RBP,Hhg1Tcf21,1110020H15Rik,Wnt5a |
| GO:0045941 | positive regulation of transcription | 2.4E-6 | Bcl3,Isl1,Klf4,Mm.215008.1,Smarcd3,BB131012,Bmp4,T,Egr1,Foxa1,Foxc1,Foxd1,Msx1,Inhba, Neurod1,Zac1,,rpplf,Mm.32654.1,Hhg1Tcf21,Zfhx1a |
| GO:0016477 | cell migration | 5.5E-5 | Isl1,Foxc1,Gbx-2,Nrp,Mm.186992.1,Plat,Pf4,Nr2f1,AI561871,Apc,Hhg1Tgfb2,Twist |
| GO:0048812 | neuron projection morphogenesis | 8.1E-5 | Isl1,Lhx2,Foxd1,Gbx-2,Nrp,AI561871,Apc,Clu,Slit2,Hhg1Tgfb2 |
| GO:0060512 | prostate gland morphogenesis | 3.5E-4 | AW146109,Bmp4,Foxa1,Hhg1Tnc |
| GO:0048667 | cell morphogenesis involved in neuron differentiation | 5.2E-4 | Isl1,Lhx2,Foxd1,Gbx-2,Nrp,AI561871,Apc,Slit2,Hhg1Tgfb2 |
| GO:0014033 | neural crest cell differentiation | 7.6E-4 | Isl1,Foxc1,Gbx-2,NP2,Hhg1 |
| GO:0015671 | oxygen transport | 3.1E-4 | Hba-x,Hbb-bh1,Hba-a1,Hbb-y |
| GO:0045661 | regulation of myoblast differentiation | 6.7E-3 | Tbx3,Bmp4,mrpplf |
| GO:0043066 | negative regulation of apoptosis | 1.1E-2 | Bcl3,Tbx3,Cdkn1a,Foxc1,Msx1,Neurod1,Apc,Clu,Snail2 |
| GO:0042325 | regulation of phosphorylation | 1.2E-2 | Bmp4,Cav,Csf1,Cdkn1a,Cdkn1c,Pdgfrb,Pdgfc,Apc,Cish3,1110020H15Rik |
| GO:0055010 | ventricular cardiac muscle morphogenesis | 1.5E-2 | Foxc1,Tnni1,Tnnt2 |
| GO:0030239 | myofibril assembly | 2.4E-2 | Myhca,1100001C23Rik,Tnnt2 |
| GO:0005859 | muscle myosin complex | 5.7E-3 | Myhca,1100001C23Rik,Myh7 |
| GO:0007439 | ectodermal digestive tract development | 1.3E-2 | 1200010K03Rik,Hhg1,Wnt5a |
