# Supplementary material for: Estimating the Quality of Reprogrammed Cells Using ES Cell Differentiation Expression Patterns
Source: PLoS One. 2011 Jan 11;6(1):e15336. doi: 10.1371/journal.pone.0015336 (PMC3023460; doi:10.1371/journal.pone.0015336)
Supplement: Table S7 — GO analysis of negative regulated genes in ES cell-derived Pancreatic islets cells Differentiation (GSE3653). (PDF) [file pone.0015336.s010.pdf]

Table S7 GO analysis of negative regulated genes in ES cell-derived Pancreatic islets cells Differentiation (GSE3653)

GO number	Description	P-value	Gene
GO:0043009	chordate embryonic development	2.7E-2	G2e3,Pou5f1,Facl4,Brca1,Cdh1,Etl4,alpha-1 gap junction,Myb,Ptch1,Six4,Tcf7,Ube2b
GO:0019903	protein phosphatase binding	1.8E-2	Cdh1,Catnb,Ipo9,Igf1r,Shoc2,Glut-3
GO:0030054	cell junction	9.5E-3	Acta1,Cdh1,Catnb,Cldn3,Crb3,alpha-1 gap junction,Gria4,Ica1,Veli3-pending,Myb, 2010005A06Rik,Shrom2,Syt9,Syt11,Ube2b,Vcl
GO:0048008	platelet-derived growth factor receptor signaling pathway	2.4E-3	Sox4,Calcr,Catnb,Deltex1,Fgf5,1700058O05Rik,Igf1r,Lpar4,Met,Ptch1,Pten,Plekha1,Ramp3,Rgs11, Sgpl1,Txnip,Tcf7,Ube2b
GO:0042981	regulation of apoptosis	3.0E-2	Ero1l,G2e3,Asc-pending,2610018G03Rik,Xnp,Brca1,Cdh1,Casp3,Dtl,Jmy,Mbd4,Pten,DXCch3,K ras,9030402K04Rik,Tcf7,Ube2b
GO:0048732	gland development	2.9E-2	Braf,Cdh1,Catnb,Hook1,Igf1r,Met,Ptch1,Pten,Shc1,DXCch3,Kras,Ybx2,Sgpl1,Socs2,Tcf7,Ube2b ,Vcl
GO:0034613	cellular protein localization	2.5E-2	Rab25,Rab39,Ap1m2,Catnb,Hook1,Ipo9,Jakmip1,Impnb,Veli3-pending,Optn,5033403E17Rik,Ra mp3,Shrom2,Stx3,Txnip
GO:0031016	pancreas development	7.0E-2	Sox4,Catnb,Igf1r,Pten,DXCch3,Kras,Shoc2,Socs2
GO:0030136	clathrin-coated vesicle	3.4E-2	Ap1m2,Calcr,Coro1a,Gria4,Ica1,Veli3-pending,Fpn1,Pphn,Syt9,Syt11,Trfr
