# Supplementary material for: Estimating the Quality of Reprogrammed Cells Using ES Cell Differentiation Expression Patterns
Source: PLoS One. 2011 Jan 11;6(1):e15336. doi: 10.1371/journal.pone.0015336 (PMC3023460; doi:10.1371/journal.pone.0015336)
Supplement: Table S8 — GO analysis of positive regulated genes in ES cell-derived Pancreatic islets cells Differentiation (GSE3653). (PDF) [file pone.0015336.s011.pdf]

Table S8 GO analysis of positive regulated genes in ES cell-derived Pancreatic islets cells Differentiation (GSE3653)

GO number	Description	P-value	Gene
GO:0001501	skeletal system development	3.4E-19	Nab2,Tbx3,Ankrd1,Axin2,Bmp-1,Bmp4,Col1a1,Col9a1,Col5a2,Dlk1,Foxc1,Foxc2,Hoxa9,Hoxb2,Msx1,Igf1,Igf2,Igfbp5,Mglap,Mn1,Myc,Mef2c,Prrx1,Prrx2,Pitx1,Pdgfra,Pdgfrb,Ptn,Pcsk5,griPGHS,RBP,Zic1,Hhg1,Tgfb2,1110020H15Rik,Wnt5a,Zfhx1a,Zbtb16
GO:0048736	appendage development	4.6E-10	Tbx3,Bmp4,Bra,Dkk1,Mecom,Foxa2,Foxc1,Foxc2,1200010K03Rik,Gbx-2,Hoxa1,Hoxa9,Hoxb2,Msx1,Itga8,Mab21l2,Mbnl,Myc,Neurod1,Otx1,Prrx1,Prrx2,Pitx1,1110003O22Rik,Pcsk5,Pcdh8,Rfx4,RBP,Nr2f1,R75022,Zic1,Fnb2,Msx2,Hhg1,Cish3,Sdccag33,Tgfb1i1,1110020H15Rik,Twist,Vegfc,Wnt5a,Zfhx1a,Zbtb16
GO:0048732	gland development	6.2E-9	CD44,Flt1Gata6,Isl1,Jun,Klf4,9430010M06Rik,Tbx3,Apoa1,Bmp4,BraCav,Csf1,Ccnd1,Mecom,Foxa1,Foxa2,Foxc1,Foxc2,Foxd1,Fzd1,Gbx-2,Hoxa9,Hoxb2,Igf1,Lama1,Lox,Mglap,Nrp,Pitx1,Pdgfra,Pcsk5,griPGHS,Pcdh8,RBP,AI561871,Snai1,Hhg1,Cish3,Tnc,Tcf21,Tgfb1i1,Tgfb2,1110020H15Rik,Tgm1,Tgm2,Tnni1,Tnnt2,Twist,Vegfc,Wnt5a
GO:0035295	tube development	1.0E-11	CD44,Flt1,Gata6,9430010M06Rik,Tbx3,Bmp4,Bra,Csf1,Foxa1,Foxa2,Foxc1,Foxc2,Foxd1,Gbx-2,Igf1,Lama1,Lox,Mglap,Nrp,Pdgfra,Pcsk5,RBP,Hhg1,Tcf21,1110020H15Rik,Tgm2,Twist,Wnt5a
GO:0007155	cell adhesion	2.9E-7	CD44,Frem1,Wisp1,AI853494,Cdh11,Col9a1,AI413331,Col6a1,Col6a2,AW229038,2410043F08Rik,BB099155,Igfbp7,Itga8,Itga9,Lama1,Ncam1,Nrp,NP2,2010012A22Rik,AI595373,Osf2-pending,Pcdh18,Pcdh19,Pcdh7,Pcdh8,Pcdhgc5,Arhb,Nid1,Tnc,Thbs1,Tgfb2,Tgfbi
GO:0043009	chordate embryonic development	1.3E-9	Gata6,Tbx3,Ankrd1,Axin2,Bmp4,Bra,Capn2,Dlk1,Mecom,Foxa2,Foxc1,Foxc2,Gbx-2,Hba-a1,Hoxa9,Hoxb2,Msx1,Mbnl,Myhca,Prrx1,Prrx2,Pdgfra,Pdgfrb,Pcsk5,Pcdh8,RBP,Hhg1,Cish3,1100001C23Rik,1110020H15Rik,Twist,Zfhx1a
GO:0007423	sensory organ development	9.9E-10	Irx5,Klf4,Meis1,Mrg1b,Sox1,Bmp4,Bra,Cryab,Foxc1,Foxc2,Gbx-2,Hoxa1,Hoxb2,Itga8,Mab21l2,Myc,Neurod1,Otx1,Prrx1,Prrx2,Pcsk5,RBP,Zic1,Apc,Hhg1,Cish3,Sdccag33,Tgfb2,1110020H15Rik,Zfhx1a
GO:0001655	urogenital system development	3.4E-12	CD44,Irx3,Pou3f3,Tbx3,ADAMTS-1,Afp,Bmp4,Cav,Csf1,Dmrta2,Foxa1,Foxc1,Foxc2,Foxd1,Fzd1,AI852300,Igf1,Itga8,Lama1,Nrp,Pdgfra,Pdgfrb,Pcsk5,RBP,Nid1,Apc,Slit2,Hhg1,Tnc,Tcf21,1110020H15Rik,Tgm2,Wnt5a,Zbtb16
GO:0035272	exocrine system development	2.0E-4	Foxc1,Igf1,Igf2,Lama1,Nrp,Hhg1,Tgm2
GO:0008191	metalloendopeptidase inhibitor activity	9.8E-3	Lxn,Timp2,Timp3
