# Supplementary material for: Estimating the Quality of Reprogrammed Cells Using ES Cell Differentiation Expression Patterns
Source: PLoS One. 2011 Jan 11;6(1):e15336. doi: 10.1371/journal.pone.0015336 (PMC3023460; doi:10.1371/journal.pone.0015336)
Supplement: Table S9 — GO analysis of negative regulated genes in ES cell-derived blast cells Differentiation (GSE8884). (PDF) [file pone.0015336.s012.pdf]

Table S9 GO analysis of negative regulated genes in ES cell-derived blast cells Differentiation (GSE8884) (212 transcripts)

GO number	GO name	P-value	GENE
GO:0048667	cell morphogenesis involved in neuron differentiation	2.1E-5	KAL1,S100A6,CXCR4,C1orf187,DCLK1,GAP43,KIF5C,NEFL,OTX2,PTPRZ1,W92748,ERBB2
GO:0045860	positive regulation of protein kinase activity	3.4E-3	CXCR4,CCND1,FGF2,GAP43,AL041761,KIAA1804,PAK1,TDGF1,ERBB2
GO:0060537	muscle tissue development	2.7E-3	CAV1,CXADR,GJC1,TNC,TDGF1,TPM1,ERBB2
GO:0016477	cell migration	3.7E-3	CER1,CXCR4,DCLK1,EDNRB,FGF19,FGF2,AL041761,NODAL,PLAU,PODXL
GO:0016481	negative regulation of transcription	3.2E-2	DNMT3B,SOX2,FGF2,FST,FOXH1,HELLS,JARID2,MBD2,NODAL,SALL4,TCF7L1
GO:0048762	mesenchymal cell differentiation	1.9E-2	CYP26A1,EDNRB,EOMES,FGF19
GO:0050880	regulation of blood vessel size	2.1E-2	CAV1,DNRB,NPPB,NTS
GO:0006935	chemotaxis	3.3E-2	KAL1,CXCL5,XCR4,EDNRB,FGF2,PLAU
GO:0030005	cellular di-, tri-valent inorganic cation homeostasis	4.1E-2	CAV1,CXCR4,CCKBR,EDNRB,LCK,MT1H,AW003173
