# Supplementary material for: Estimating the Quality of Reprogrammed Cells Using ES Cell Differentiation Expression Patterns
Source: PLoS One. 2011 Jan 11;6(1):e15336. doi: 10.1371/journal.pone.0015336 (PMC3023460; doi:10.1371/journal.pone.0015336)
Supplement: Table S10 — GO analysis of positive regulated genes in ES cell-derived blast cells Differentiation (GSE8884). (PDF) [file pone.0015336.s013.pdf]

Table S10 GO analysis of positive regulated genes in ES cell-derived blast cells Differentiation (GSE8884) (513 transcripts)

GO number	GO name	P-value	GENE
GO:0015671	oxygen transport	1.5E-7	HBA2,HBB,HBD,HBE1,HBG1,HBM,HBZ
GO:0042981	regulation of apoptosis	2.8E-4	BNIP3L,BTG2,CITED2,SMAD6,TBX3,AMIGO2,ANXA1,ANXA4,ALOX12,CBX4,F2R,EGFR,HGF,HIPK2,IGF2,IFI6,MSX1,MEF2C,BF698797,PF4,PCSK6,SCG2,SNCA,THBS1
GO:0048534	hemopoietic or lymphoid organ development	1.2E-3	KLF1,LMO2,TAL1,TIMP1,AF130113,EGR1,EPAS1,HBZ,HOXB7,ID2,IFI16,MMP9,PLEK,SPTA1,SYK,TGFB1,D50683,TGFBR3,AI492388
GO:0060323	head morphogenesis	4.0E-3	ARID5B,ASPH,COL1A1,PDGFRA
GO:0030323	respiratory tube development	4.1E-3	HOPX,AI934569,CRISPLD2,EPAS1,FOXF1,LOX,PDGFRA,RBP4,D50683,ZFPM2
GO:0070201	regulation of establishment of protein localization	4.1E-3	ADAM9,MXI1,APOA1,APOA2,IGF2,LCP1,PKIA,RBP4,SRGN,TGFB1,VEGFC
GO:0016481	negative regulation of transcription	2.1E-3	ARID5B,CITED2,HOPX,NM_020149,TBX3,CBX4,CDKN1B,CDKN1C,EGR1,GFI1B,HEY1,HAND1,HBZ,HIPK2,ID1,ID2,ID4,LEF1,MBD2,MSX1,M,F2C,PKIA,SNAI2,TGFB1,ZEB2,AI492388,ZFPM2
GO:0065005	protein-lipid complex assembly	5.2E-3	ABCA1,APOA1,APOA2,APOB
GO:0032963	collagen metabolic process	1.0E-2	BE221212,COL3A1,COL5A1,C8orf57,MMP9
GO:0009749	response to glucose stimulus	4.9E-2	APOA2,AI439556,THBS1,TGFB1,D50683
