# Supplementary material for: Estimating the Quality of Reprogrammed Cells Using ES Cell Differentiation Expression Patterns
Source: PLoS One. 2011 Jan 11;6(1):e15336. doi: 10.1371/journal.pone.0015336 (PMC3023460; doi:10.1371/journal.pone.0015336)
Supplement: Table S11 — GO analysis of negative regulated genes in ES cell-derived neuron rosettes Differentiation (GSE9940). (PDF) [file pone.0015336.s014.pdf]

Table S11 GO analysis of negative regulated genes in ES cell-derived neuron rosettes Differentiation (GSE9940) (182 transcripts)

GO number	GO name	P-value	GENE
GO:0045935	positive regulation of nucleobase, nucleoside, nucleotide and nucleic acid metabolic process	3.3E-7	GLIS3,AL832482,NKX2-1,AW149422,PRDM16,AA651631,ASCL1,DLX2,EBF1,AI758408,HES5, NEUROD1,PAX6,RFX4,ZEB1
GO:0016481	negative regulation of transcription	5.3E-7	GLIS3,AL832482,NKX2-1,AW149422,PRDM16,AA651631,DLX1,DLX2,HES5,NR2E1,NR2F1,Z EB1,AW244016
GO:0045941	positive regulation of transcription	6.9E-7	GLIS3,AL832482,NKX2-1,AW149422,PRDM16,AA651631,ASCL1,DLX2,EBF1,HES5,NEUROD 1,PAX6,RFX4,ZEB1
GO:0016477	cell migration	2.8E-6	NKX2-1,POU3F2,AW149422,SOX1,ASCL1,ARX,DCX,EMX2,NR2E1,NR2F1,PAX6,RELN,AI692 523
GO:0060284	regulation of cell development	2.9E-5	ASCL1,,DLX1,DLX2,FOXG1,HES5,NEUROD1,NR2E1,PAX6
GO:0000904	cell morphogenesis involved in differentiation	8.7E-5	FEZF2,LHX2,NKX2-1,ARX,DCX,NM_004471,NR2E1,PAX6,RELN,AI692523,SNAP25
GO:0007601	visual perception	3.4E-4	SIX6,COL2A1,CRYBA1,BE674583,LUM,NR2E1,PAX6
GO:0048704	embryonic skeletal system morphogenesis	2.6E-2	COL2A1,DLX2,AI806174
GO:0008076	voltage-gated potassium channel complex	3.3E-2	KCNN3,AB013889,R52825,SNAP25
GO:0032526	response to retinoic acid	4.3E-2	HSD17B2,LRP2,AI792842
