# Supplementary material for: Estimating the Quality of Reprogrammed Cells Using ES Cell Differentiation Expression Patterns
Source: PLoS One. 2011 Jan 11;6(1):e15336. doi: 10.1371/journal.pone.0015336 (PMC3023460; doi:10.1371/journal.pone.0015336)
Supplement: Table S12 — GO analysis of positive regulated genes in ES cell-derived neuron rosettes Differentiation (GSE9940). (PDF) [file pone.0015336.s015.pdf]

Table S12 GO analysis of positive regulated genes in ES cell-derived neuron rosettes Differentiation (GSE9940) (156 transcripts)

GO number	GO name	P-value	GENE
GO:0019748	secondary metabolic process	4.0E-3	CYP26A1,INDO,LRAT,AK090801,RARRES2
GO:0019882	antigen processing and presentation	5.9E-4	MICB,IFI30,HLA-DOA,HLA-DQB1,PSMB8,PSMB9
GO:0030001	metal ion transport	4.9E-3	GRID2,HCN1,ITPR3,LCK,KCTD14,KCNN2,KCNG3,SCNN1A,SLC13A3,R00975,TRPC6,TMEM37
GO:0042981	regulation of apoptosis	6.5E-2	BIK,HTATIP2,MICB,PYCARD,RABGAP1L,CDH1,FLT1,GAL,GRID2,INDO,LCK,MAP4K1,PMAIP1,PIM2,PSMB8,PSMB9,TERF1,TDGF1
GO:0044459	plasma membrane part	1.7E-2	HTR1D,CD72,CD9,EPHA1,GPR64,MICB,AP1M2,CDH1,CLDN7,TACSTD1,EPB41L5,FLT1,GRID2,ITPR3,LCK,HLA-DOA,HLA-DQB1,MAP7,KCTD1,,KCNG3,SCNN1A,SLC13A3,SNTG2,TDGF1,TMPRSS11E,TMPRSS2,TNFSF11
GO:0005576	extracellular region	1.1E-2	HTR1D,MICB,TIMP4,CCL26,CXCL5,CXCL6,MGC45438,CR2,CDA,DMKN,AA579773,FGF1,FLT1,GAL,GRID2,INDO,IFI30,KLKB1,LEFTY1,LEC,1,HLA-DOA,HLA-DQB1,MMP1,OLFML3,PSMB8,PSMB9,RARRES2,SCGB3A2,SPINT1,SMPDL3B,TDGF1,TMPRSS11E,TMPRSS2,TNFSF11, FLJ14712
