# Supplementary material for: Estimating the Quality of Reprogrammed Cells Using ES Cell Differentiation Expression Patterns
Source: PLoS One. 2011 Jan 11;6(1):e15336. doi: 10.1371/journal.pone.0015336 (PMC3023460; doi:10.1371/journal.pone.0015336)
Supplement: Table S13 — Positive regulated genes in ES cell-derived blast cell differentiation (GSE8884). (PDF) [file pone.0015336.s016.pdf]

Table S13 Positive regulated genes in ES cell-derived blast cell differentiation (GSE8884)(556 transcript)

Probe Set_ID	Gene Name	Weight	P-value	FDR<0.1
213515_x_at	HBG2	0.067504	1.08E-54	3.66E-06
204419_x_at	HBG2	0.065997	2.62E-52	7.32E-06
206647_at	HBZ	0.064815	1.78E-50	1.10E-05
205919_at	HBE1	0.06376	7.20E-49	1.46E-05
204848_x_at	HBG1	0.063099	7.11E-48	1.83E-05
214414_x_at	HBA1	0.059513	1.15E-42	2.20E-05
201744_s_at	LUM	0.058029	1.34E-40	2.56E-05
210095_s_at	IGFBP3	0.057295	1.35E-39	2.93E-05
202409_at	IGF2	0.055814	1.30E-37	3.30E-05
211161_s_at	AF130082	0.05556	2.81E-37	3.66E-05
211745_x_at	HBA2	0.05515	9.73E-37	4.03E-05
209458_x_at	HBA2	0.055019	1.44E-36	4.39E-05
217414_x_at	HBA2	0.054839	2.47E-36	4.76E-05
224997_x_at	H19	0.054756	3.17E-36	5.13E-05
204018_x_at	HBA1	0.052101	7.26E-33	5.49E-05
211699_x_at	HBA2	0.052063	8.08E-33	5.86E-05
224646_x_at	H19	0.051606	2.94E-32	6.23E-05
214146_s_at	PPBP	0.048144	3.63E-28	6.59E-05
220138_at	HAND1	0.047922	6.50E-28	6.96E-05
212143_s_at	IGFBP3	0.047049	6.23E-27	7.32E-05
215076_s_at	COL3A1	0.046248	4.78E-26	7.69E-05
206390_x_at	PF4	0.045189	6.71E-25	8.06E-05
206145_at	RHAG	0.043677	2.61E-23	8.42E-05
204694_at	AFP	0.04183	1.94E-21	8.79E-05
228708_at	BF438386	0.041689	2.67E-21	9.16E-05
200878_at	EPAS1	0.041445	4.64E-21	9.52E-05
205523_at	HAPLN1	0.041082	1.05E-20	9.89E-05
214407_x_at	GYPB	0.040901	1.57E-20	0.0001025
209560_s_at	DLK1	0.040094	9.37E-20	0.0001062
204114_at	NID2	0.040003	1.14E-19	0.0001099
204249_s_at	LMO2	0.039251	5.81E-19	0.0001135
209660_at	TTR	0.038178	5.58E-18	0.0001172
201852_x_at	COL3A1	0.038165	5.74E-18	0.0001209
206283_s_at	TAL1	0.037794	1.24E-17	0.0001245
219612_s_at	FGG	0.037137	4.74E-17	0.0001282
203698_s_at	FRZB	0.037015	6.06E-17	0.0001318
203697_at	FRZB	0.036473	1.80E-16	0.0001355
206834_at	HBD	0.036268	2.69E-16	0.0001392
201427_s_at	SEPP1	0.036096	3.78E-16	0.0001428
228186_s_at	RSPO3	0.035686	8.44E-16	0.0001465
201288_at	ARHGDIB	0.035378	1.53E-15	0.0001501

211560_s_at	AF130113	0.035313	1.74E-15	0.0001538
206332_s_at	IFI16	0.035213	2.11E-15	0.0001575
208966_x_at	IFI16	0.035078	2.73E-15	0.0001611
201893_x_at	DCN	0.034935	3.58E-15	0.0001648
201150_s_at	TIMP3	0.03486	4.13E-15	0.0001685
203471_s_at	PLEK	0.034775	4.85E-15	0.0001721
211821_x_at	GYPA	0.034481	8.45E-15	0.0001758
201147_s_at	TIMP3	0.034103	1.71E-14	0.0001794
206239_s_at	SPINK1	0.033972	2.19E-14	0.0001831
207069_s_at	SMAD6	0.033802	2.99E-14	0.0001868
223044_at	SLC40A1	0.033755	3.26E-14	0.0001904
204988_at	FGB	0.033746	3.32E-14	0.0001941
230147_at	F2RL2	0.03351	5.12E-14	0.0001978
205108_s_at	APOB	0.033167	9.53E-14	0.0002014
205984_at	CRHBP	0.032982	1.33E-13	0.0002051
215446_s_at	LOX	0.032934	1.45E-13	0.0002087
201858_s_at	SRGN	0.032799	1.85E-13	0.0002124
218002_s_at	CXCL14	0.032791	1.87E-13	0.0002161
219140_s_at	RBP4	0.032572	2.77E-13	0.0002197
1555812_a_at	ARHGDIB	0.032388	3.83E-13	0.0002234
212192_at	KCTD12	0.032239	4.98E-13	0.0002271
204466_s_at	SNCA	0.032224	5.12E-13	0.0002307
216238_s_at	FGB	0.032094	6.43E-13	0.0002344
205650_s_at	FGA	0.032013	7.40E-13	0.000238
201506_at	TGFBI	0.032012	7.42E-13	0.0002417
201721_s_at	LAPTM5	0.031971	7.96E-13	0.0002454
222529_at	SLC25A37	0.031812	1.05E-12	0.000249
229450_at	IFIT3	0.031721	1.23E-12	0.0002527
230690_at	TUBB1	0.031713	1.25E-12	0.0002563
212195_at	IL6ST	0.031674	1.33E-12	0.00026
213139_at	SNAI2	0.031656	1.38E-12	0.0002637
201859_at	SRGN	0.03133	2.41E-12	0.0002673
209116_x_at	HBB	0.031176	3.13E-12	0.000271
227404_s_at	EGR1	0.031143	3.31E-12	0.0002747
209386_at	TM4SF1	0.03068	7.21E-12	0.0002783
1554012_at	RSPO2	0.030637	7.76E-12	0.000282
206049_at	SELP	0.030514	9.51E-12	0.0002856
222484_s_at	CXCL14	0.030391	1.17E-11	0.0002893
203477_at	COL15A1	0.030347	1.26E-11	0.000293
221577_x_at	GDF15	0.030346	1.26E-11	0.0002966
204115_at	GNG11	0.030289	1.38E-11	0.0003003
222528_s_at	SLC25A37	0.030288	1.38E-11	0.000304
230204_at	AU144114	0.030127	1.80E-11	0.0003076
221920_s_at	SLC25A37	0.029941	2.45E-11	0.0003113

213258_at	TFPI	0.029624	4.09E-11	0.0003149
225911_at	NPNT	0.029377	6.08E-11	0.0003186
237403_at	GFI1B	0.0293	6.88E-11	0.0003223
215034_s_at	TM4SF1	0.029079	9.79E-11	0.0003259
226751_at	CNRIP1	0.02906	1.01E-10	0.0003296
219454_at	EGFL6	0.029034	1.05E-10	0.0003333
226625_at	TGFBR3	0.028999	1.11E-10	0.0003369
209199_s_at	MEF2C	0.02886	1.38E-10	0.0003406
202411_at	IFI27	0.028751	1.64E-10	0.0003442
1558048_x_at	BG389789	0.028741	1.67E-10	0.0003479
219992_at	TAC3	0.028374	2.95E-10	0.0003516
204627_s_at	ITGB3	0.028228	3.70E-10	0.0003552
208965_s_at	IFI16	0.028183	3.96E-10	0.0003589
213620_s_at	ICAM2	0.028043	4.91E-10	0.0003626
209930_s_at	NFE2	0.02801	5.16E-10	0.0003662
228098_s_at	MYLIP	0.027922	5.90E-10	0.0003699
203680_at	PRKAR2B	0.027869	6.39E-10	0.0003735
216833_x_at	GYPB	0.027814	6.95E-10	0.0003772
231274_s_at	R92925	0.027799	7.11E-10	0.0003809
1565358_at	RARA	0.027676	8.56E-10	0.0003845
210395_x_at	AF116676	0.027617	9.36E-10	0.0003882
203973_s_at	CEBPD	0.02749	1.13E-09	0.0003918
204319_s_at	RGS10	0.027346	1.40E-09	0.0003955
205524_s_at	HAPLN1	0.027249	1.62E-09	0.0003992
219791_s_at	NBLA00301	0.027155	1.86E-09	0.0004028
207558_s_at	PITX2	0.027058	2.15E-09	0.0004065
204534_at	VTN	0.027051	2.17E-09	0.0004102
219466_s_at	APOA2	0.026995	2.36E-09	0.0004138
234989_at	TncRNA	0.026986	2.39E-09	0.0004175
219465_at	APOA2	0.02698	2.41E-09	0.0004211
223303_at	FERMT3	0.026851	2.91E-09	0.0004248
211820_x_at	GYPA	0.026803	3.13E-09	0.0004285
242093_at	SYTL5	0.026791	3.18E-09	0.0004321
224348_s_at	AF116709	0.026735	3.46E-09	0.0004358
203325_s_at	COL5A1	0.026612	4.13E-09	0.0004395
201565_s_at	ID2	0.026606	4.17E-09	0.0004431
227561_at	DDR2	0.026604	4.18E-09	0.0004468
201438_at	COL6A3	0.02655	4.52E-09	0.0004504
207459_x_at	GYPB	0.026209	7.37E-09	0.0004541
218136_s_at	NM_018579	0.026209	7.37E-09	0.0004578
209291_at	ID4	0.026188	7.60E-09	0.0004614
212224_at	ALDH1A1	0.026179	7.70E-09	0.0004651
204415_at	IFI6	0.026172	7.77E-09	0.0004688
204955_at	SRPX	0.026055	9.18E-09	0.0004724

209200_at	MEF2C	0.025985	1.01E-08	0.0004761
212614_at	ARID5B	0.025929	1.10E-08	0.0004797
210664_s_at	TFPI	0.025831	1.26E-08	0.0004834
211597_s_at	HOPX	0.025538	1.90E-08	0.0004871
207206_s_at	ALOX12	0.025534	1.91E-08	0.0004907
219682_s_at	TBX3	0.025445	2.16E-08	0.0004944
207480_s_at	NM_020149	0.025439	2.18E-08	0.000498
206167_s_at	NM_001174	0.025438	2.18E-08	0.0005017
208982_at	AW574504	0.025391	2.33E-08	0.0005054
204677_at	CDH5	0.025353	2.45E-08	0.000509
205837_s_at	GYPA	0.025292	2.67E-08	0.0005127
204450_x_at	APOA1	0.025266	2.77E-08	0.0005164
219938_s_at	PSTPIP2	0.025222	2.94E-08	0.00052
204731_at	TGFBR3	0.025204	3.01E-08	0.0005237
220496_at	CLEC1B	0.025203	3.01E-08	0.0005273
225895_at	SYNPO2	0.025129	3.34E-08	0.000531
205923_at	RELN	0.025069	3.63E-08	0.0005347
204624_at	ATP7B	0.025068	3.63E-08	0.0005383
211896_s_at	DCN	0.025035	3.79E-08	0.000542
215813_s_at	PTGS1	0.025004	3.96E-08	0.0005457
217073_x_at	APOA1	0.025001	3.97E-08	0.0005493
209355_s_at	PPAP2B	0.024955	4.23E-08	0.000553
226179_at	SLC25A37	0.024892	4.61E-08	0.0005566
203548_s_at	LPL	0.024838	4.96E-08	0.0005603
207808_s_at	PROS1	0.024833	5.00E-08	0.000564
1553171_x_at	LRRN4	0.024794	5.27E-08	0.0005676
204298_s_at	LOX	0.024783	5.34E-08	0.0005713
230942_at	CMTM5	0.024751	5.58E-08	0.000575
206806_at	DGKI	0.024598	6.85E-08	0.0005786
212298_at	BE620457	0.024531	7.49E-08	0.0005823
216063_at	HBBP1	0.024475	8.08E-08	0.0005859
219778_at	ZFPM2	0.024457	8.28E-08	0.0005896
201280_s_at	DAB2	0.024439	8.47E-08	0.0005933
204938_s_at	PLN	0.024391	9.03E-08	0.0005969
203037_s_at	MTSS1	0.02435	9.53E-08	0.0006006
212196_at	IL6ST	0.024281	1.04E-07	0.0006043
213348_at	CDKN1C	0.024175	1.20E-07	0.0006079
201185_at	HTRA1	0.024114	1.30E-07	0.0006116
212230_at	PPAP2B	0.024054	1.41E-07	0.0006152
210302_s_at	MAB21L2	0.02401	1.49E-07	0.0006189
224823_at	MYLK	0.023993	1.52E-07	0.0006226
205128_x_at	PTGS1	0.023973	1.57E-07	0.0006262
1558678_s_at	BE708432	0.023922	1.67E-07	0.0006299
224759_s_at	C12orf23	0.023887	1.75E-07	0.0006335

225681_at	CTHRC1	0.023883	1.76E-07	0.0006372
204236_at	FLI1	0.023863	1.81E-07	0.0006409
205504_at	BTK	0.023839	1.87E-07	0.0006445
212473_s_at	MICAL2	0.023787	2.00E-07	0.0006482
211254_x_at	RHAG	0.023758	2.07E-07	0.0006519
205649_s_at	FGA	0.023719	2.18E-07	0.0006555
202410_x_at	IGF2	0.023716	2.19E-07	0.0006592
220794_at	GREM2	0.023634	2.43E-07	0.0006628
209278_s_at	TFPI2	0.023589	2.58E-07	0.0006665
229452_at	TMEM88	0.023583	2.59E-07	0.0006702
203936_s_at	MMP9	0.023478	2.97E-07	0.0006738
208501_at	GFI1B	0.023452	3.07E-07	0.0006775
211813_x_at	DCN	0.023437	3.13E-07	0.0006812
200839_s_at	CTSB	0.023417	3.21E-07	0.0006848
223670_s_at	HEMGN	0.023416	3.21E-07	0.0006885
203153_at	IFIT1	0.02341	3.24E-07	0.0006921
201700_at	CCND3	0.023271	3.87E-07	0.0006958
226702_at	CMPK2	0.02324	4.02E-07	0.0006995
226777_at	AA147933	0.023127	4.63E-07	0.0007031
205269_at	LCP2	0.023121	4.67E-07	0.0007068
227443_at	C9orf150	0.023052	5.09E-07	0.0007105
211075_s_at	CD47	0.022941	5.85E-07	0.0007141
209318_x_at	BG547855	0.022904	6.13E-07	0.0007178
228854_at	AI492388	0.022897	6.18E-07	0.0007214
205838_at	GYPA	0.022872	6.37E-07	0.0007251
202404_s_at	COL1A2	0.022858	6.49E-07	0.0007288
225242_s_at	CCDC80	0.022833	6.70E-07	0.0007324
212489_at	COL5A1	0.022819	6.81E-07	0.0007361
225442_at	DDR2	0.022804	6.94E-07	0.0007398
210504_at	KLF1	0.022738	7.53E-07	0.0007434
207172_s_at	CDH11	0.022726	7.64E-07	0.0007471
209685_s_at	PRKCB1	0.022646	8.44E-07	0.0007507
210809_s_at	POSTN	0.022637	8.53E-07	0.0007544
206207_at	CLC	0.022614	8.77E-07	0.0007581
201720_s_at	LAPTM5	0.02258	9.15E-07	0.0007617
201061_s_at	STOM	0.022495	1.02E-06	0.0007654
213921_at	SST	0.022427	1.10E-06	0.000769
1556499_s_at	BE221212	0.022401	1.14E-06	0.0007727
204232_at	FCER1G	0.022395	1.15E-06	0.0007764
212488_at	COL5A1	0.022387	1.16E-06	0.00078
202310_s_at	COL1A1	0.022371	1.18E-06	0.0007837
208885_at	LCP1	0.022313	1.27E-06	0.0007874
202555_s_at	NM_005965	0.022262	1.35E-06	0.000791
209684_at	RIN2	0.022251	1.37E-06	0.0007947

203504_s_at	ABCA1	0.022242	1.38E-06	0.0007983
225809_at	DKFZP564O0823	0.022214	1.43E-06	0.000802
203074_at	ANXA8L2	0.022209	1.44E-06	0.0008057
205239_at	AREG	0.022169	1.51E-06	0.0008093
217757_at	A2M	0.022161	1.53E-06	0.000813
207173_x_at	CDH11	0.022139	1.57E-06	0.0008167
203887_s_at	THBD	0.022124	1.59E-06	0.0008203
221760_at	MAN1A1	0.022103	1.64E-06	0.000824
222717_at	SDPR	0.022065	1.71E-06	0.0008276
208981_at	PECAM1	0.022057	1.73E-06	0.0008313
238197_at	GATA5	0.022042	1.76E-06	0.000835
221748_s_at	TNS1	0.02201	1.83E-06	0.0008386
235210_s_at	RPESP	0.022009	1.83E-06	0.0008423
212226_s_at	PPAP2B	0.021943	1.98E-06	0.000846
218109_s_at	MFSD1	0.021937	2.00E-06	0.0008496
201149_s_at	TIMP3	0.021918	2.04E-06	0.0008533
1554966_a_at	FILIP1L	0.021915	2.05E-06	0.0008569
213992_at	COL4A6	0.021887	2.12E-06	0.0008606
201279_s_at	DAB2	0.021809	2.32E-06	0.0008643
212667_at	AL575922	0.021729	2.56E-06	0.0008679
204220_at	GMFG	0.021622	2.90E-06	0.0008716
212573_at	ENDOD1	0.021484	3.41E-06	0.0008752
212956_at	TBC1D9	0.021466	3.48E-06	0.0008789
203989_x_at	F2R	0.021432	3.62E-06	0.0008826
203304_at	BAMBI	0.021304	4.20E-06	0.0008862
210757_x_at	DAB2	0.021244	4.50E-06	0.0008899
218309_at	CAMK2N1	0.021223	4.62E-06	0.0008936
213603_s_at	RAC2	0.021205	4.71E-06	0.0008972
201860_s_at	PLAT	0.0212	4.74E-06	0.0009009
203549_s_at	LPL	0.0212	4.74E-06	0.0009045
213238_at	ATP10D	0.021149	5.03E-06	0.0009082
209047_at	AQP1	0.021113	5.24E-06	0.0009119
205694_at	TYRP1	0.021014	5.87E-06	0.0009155
204187_at	GMPR	0.020903	6.67E-06	0.0009192
231735_s_at	NM_014086	0.020871	6.91E-06	0.0009229
204135_at	FILIP1L	0.020859	7.01E-06	0.0009265
204720_s_at	DNAJC6	0.020766	7.78E-06	0.0009302
240336_at	HBM	0.020667	8.70E-06	0.0009338
213857_s_at	CD47	0.020618	9.19E-06	0.0009375
244050_at	PTPLAD2	0.020605	9.33E-06	0.0009412
204073_s_at	C11orf9	0.020599	9.39E-06	0.0009448
210830_s_at	PON2	0.020455	1.10E-05	0.0009485
202554_s_at	GSTM3	0.020444	1.12E-05	0.0009522
217232_x_at	HBB	0.020431	1.13E-05	0.0009558

230895_at	AI760252	0.020421	1.15E-05	0.0009595
202733_at	P4HA2	0.020407	1.16E-05	0.0009631
228527_s_at	SLC25A37	0.020404	1.17E-05	0.0009668
202948_at	IL1R1	0.020337	1.26E-05	0.0009705
201891_s_at	B2M	0.020335	1.26E-05	0.0009741
209387_s_at	TM4SF1	0.020269	1.36E-05	0.0009778
202403_s_at	COL1A2	0.020262	1.37E-05	0.0009815
205374_at	SLN	0.020224	1.43E-05	0.0009851
208944_at	D50683	0.02016	1.53E-05	0.0009888
226853_at	BMP2K	0.020113	1.61E-05	0.0009924
221541_at	CRISPLD2	0.020111	1.61E-05	0.0009961
201324_at	EMP1	0.020082	1.66E-05	0.0009998
201876_at	PON2	0.020072	1.68E-05	0.0010034
213110_s_at	COL4A5	0.02007	1.69E-05	0.0010071
223939_at	SUCNR1	0.020024	1.77E-05	0.0010107
202769_at	CCNG2	0.019967	1.89E-05	0.0010144
219403_s_at	HPSE	0.019965	1.89E-05	0.0010181
37892_at	COL11A1	0.01993	1.96E-05	0.0010217
228863_at	PCDH17	0.019898	2.03E-05	0.0010254
208983_s_at	PECAM1	0.019869	2.10E-05	0.0010291
204612_at	PKIA	0.019864	2.11E-05	0.0010327
206702_at	TEK	0.019864	2.11E-05	0.0010364
223809_at	RGS18	0.019765	2.35E-05	0.00104
238447_at	RBMS3	0.019684	2.56E-05	0.0010437
210044_s_at	LYL1	0.019679	2.57E-05	0.0010474
201060_x_at	AI537887	0.019667	2.61E-05	0.001051
204904_at	GJA4	0.019644	2.67E-05	0.0010547
212651_at	RHOBTB1	0.019632	2.71E-05	0.0010584
227750_at	KALRN	0.019611	2.77E-05	0.001062
218501_at	ARHGEF3	0.019593	2.82E-05	0.0010657
218353_at	NM_025226	0.019566	2.91E-05	0.0010693
205612_at	MMRN1	0.019514	3.07E-05	0.001073
220330_s_at	SAMSN1	0.01951	3.08E-05	0.0010767
219534_x_at	CDKN1C	0.019503	3.11E-05	0.0010803
242335_at	SLC25A37	0.019484	3.17E-05	0.001084
201148_s_at	TIMP3	0.019484	3.17E-05	0.0010877
212335_at	GNS	0.019469	3.22E-05	0.0010913
212188_at	KCTD12	0.019448	3.29E-05	0.001095
229580_at	R71596	0.019448	3.29E-05	0.0010986
201109_s_at	THBS1	0.019442	3.31E-05	0.0011023
217989_at	HSD17B11	0.019354	3.64E-05	0.001106
209189_at	FOS	0.019274	3.95E-05	0.0011096
202458_at	PRSS23	0.019255	4.03E-05	0.0011133
200911_s_at	TACC1	0.019231	4.14E-05	0.001117

217826_s_at	UBE2J1	0.019228	4.15E-05	0.0011206
204517_at	BE962749	0.019227	4.15E-05	0.0011243
201566_x_at	ID2	0.019211	4.22E-05	0.0011279
44783_s_at	HEY1	0.0192	4.27E-05	0.0011316
201278_at	DAB2	0.019144	4.53E-05	0.0011353
210446_at	GATA1	0.019121	4.64E-05	0.0011389
202878_s_at	CD93	0.019042	5.04E-05	0.0011426
212843_at	NCAM1	0.019041	5.04E-05	0.0011462
223754_at	MGC13057	0.019026	5.12E-05	0.0011499
222108_at	AMIGO2	0.019019	5.16E-05	0.0011536
205935_at	FOXF1	0.019015	5.18E-05	0.0011572
216231_s_at	B2M	0.019014	5.19E-05	0.0011609
204417_at	GALC	0.019013	5.19E-05	0.0011646
201010_s_at	TXNIP	0.018973	5.41E-05	0.0011682
202664_at	WIPF1	0.018954	5.51E-05	0.0011719
201008_s_at	TXNIP	0.018939	5.60E-05	0.0011755
203717_at	DPP4	0.01893	5.65E-05	0.0011792
221766_s_at	FAM46A	0.018914	5.75E-05	0.0011829
203813_s_at	SLIT3	0.018877	5.97E-05	0.0011865
227654_at	FAM65C	0.018853	6.12E-05	0.0011902
223130_s_at	MYLIP	0.018825	6.30E-05	0.0011939
31845_at	ELF4	0.018798	6.47E-05	0.0011975
203042_at	LAMP2	0.018785	6.56E-05	0.0012012
221478_at	BNIP3L	0.01878	6.60E-05	0.0012048
226279_at	PRSS23	0.018779	6.60E-05	0.0012085
226188_at	HSPC159	0.018707	7.10E-05	0.0012122
226068_at	SYK	0.018648	7.55E-05	0.0012158
40687_at	GJA4	0.018639	7.61E-05	0.0012195
203505_at	ABCA1	0.01863	7.68E-05	0.0012232
200838_at	CTSB	0.018625	7.72E-05	0.0012268
204320_at	COL11A1	0.018623	7.74E-05	0.0012305
211682_x_at	UGT2B28	0.018606	7.87E-05	0.0012341
202855_s_at	SLC16A3	0.018594	7.97E-05	0.0012378
225033_at	LOC286167	0.018539	8.43E-05	0.0012415
208949_s_at	LGALS3	0.018523	8.56E-05	0.0012451
206032_at	DSC3	0.018522	8.57E-05	0.0012488
224559_at	AF001540	0.018424	9.45E-05	0.0012524
213455_at	FAM114A1	0.018413	9.56E-05	0.0012561
215111_s_at	TSC22D1	0.018403	9.65E-05	0.0012598
218700_s_at	RAB7L1	0.018397	9.71E-05	0.0012634
206033_s_at	DSC3	0.018373	9.95E-05	0.0012671
225171_at	ARHGAP18	0.018368	0.00010005	0.0012708
234994_at	TMEM200A	0.018337	0.00010315	0.0012744
203455_s_at	SAT1	0.018335	0.00010338	0.0012781

210088_x_at	MYL4	0.018321	0.00010479	0.0012817
226849_at	DENND1A	0.018307	0.00010627	0.0012854
202766_s_at	FBN1	0.018279	0.00010928	0.0012891
209691_s_at	DOK4	0.018268	0.0001105	0.0012927
209960_at	HGF	0.018233	0.00011441	0.0012964
218718_at	PDGFC	0.018221	0.00011573	0.0013001
209869_at	ADRA2A	0.018185	0.00012	0.0013037
204069_at	MEIS1	0.01818	0.00012055	0.0013074
204863_s_at	IL6ST	0.018165	0.00012239	0.001311
228582_x_at	AI475544	0.018141	0.00012535	0.0013147
201041_s_at	DUSP1	0.018138	0.00012565	0.0013184
207002_s_at	PLAGL1	0.018135	0.00012603	0.001322
209806_at	HIST1H2BK	0.018128	0.00012697	0.0013257
204468_s_at	TIE1	0.018125	0.00012731	0.0013294
207854_at	GYPE	0.01812	0.00012793	0.001333
224567_x_at	MALAT1	0.01812	0.00012798	0.0013367
202484_s_at	MBD2	0.0181	0.00013041	0.0013403
223129_x_at	MYLIP	0.018068	0.00013462	0.001344
205366_s_at	HOXB6	0.018058	0.00013593	0.0013477
235209_at	RPESP	0.018039	0.00013849	0.0013513
209710_at	GATA2	0.018001	0.00014384	0.001355
209676_at	TFPI	0.017984	0.00014624	0.0013587
213183_s_at	CDKN1C	0.017887	0.00016068	0.0013623
228195_at	MGC13057	0.017874	0.00016271	0.001366
207815_at	PF4V1	0.017869	0.00016361	0.0013696
209604_s_at	GATA3	0.017849	0.00016677	0.0013733
204637_at	CGA	0.017831	0.00016969	0.001377
204939_s_at	PLN	0.017825	0.00017074	0.0013806
205453_at	HOXB2	0.017823	0.00017101	0.0013843
229576_s_at	TBX3	0.01777	0.00018006	0.0013879
202007_at	NID1	0.017761	0.00018164	0.0013916
202381_at	ADAM9	0.017752	0.00018315	0.0013953
204122_at	TYROBP	0.017709	0.00019096	0.0013989
207467_x_at	CAST	0.017687	0.0001951	0.0014026
226928_x_at	SLC25A37	0.017678	0.00019676	0.0014063
209897_s_at	SLIT2	0.017654	0.0002014	0.0014099
211000_s_at	IL6ST	0.017625	0.00020691	0.0014136
206494_s_at	ITGA2B	0.017608	0.00021031	0.0014172
200743_s_at	TPP1	0.017608	0.00021044	0.0014209
203835_at	LRRC32	0.017578	0.00021656	0.0014246
231866_at	LNPEP	0.017577	0.00021667	0.0014282
202336_s_at	PAM	0.01756	0.0002202	0.0014319
205592_at	SLC4A1	0.017556	0.00022106	0.0014356
225922_at	FNIP2	0.017548	0.00022283	0.0014392

206039_at	RAB33A	0.017533	0.00022594	0.0014429
223168_at	RHOU	0.017502	0.00023274	0.0014465
228850_s_at	SLIT2	0.017486	0.00023638	0.0014502
204017_at	KDELR3	0.017477	0.00023829	0.0014539
210881_s_at	IGF2	0.017475	0.00023875	0.0014575
206698_at	XK	0.017465	0.00024116	0.0014612
206561_s_at	AKR1B10	0.017385	0.00026013	0.0014649
213274_s_at	CTSB	0.017303	0.00028105	0.0014685
204588_s_at	SLC7A7	0.017278	0.00028776	0.0014722
203603_s_at	ZEB2	0.017268	0.00029056	0.0014758
234897_s_at	AF129756	0.017264	0.00029168	0.0014795
203924_at	GSTA2	0.017254	0.00029423	0.0014832
210592_s_at	SAT1	0.017221	0.00030359	0.0014868
213975_s_at	AV711904	0.017206	0.00030775	0.0014905
203196_at	ABCC4	0.017184	0.00031424	0.0014941
213182_x_at	CDKN1C	0.01713	0.00033054	0.0014978
226492_at	SEMA6D	0.017079	0.00034663	0.0015015
205883_at	ZBTB16	0.017061	0.00035233	0.0015051
224002_s_at	FKBP7	0.017058	0.00035348	0.0015088
202201_at	BLVRB	0.017039	0.00035955	0.0015125
212472_at	MICAL2	0.017037	0.00036029	0.0015161
202311_s_at	COL1A1	0.01699	0.00037635	0.0015198
213702_x_at	AI934569	0.016969	0.00038358	0.0015234
1554899_s_at	BC020763	0.016957	0.00038798	0.0015271
204467_s_at	SNCA	0.016957	0.00038806	0.0015308
227923_at	SHANK3	0.016947	0.00039159	0.0015344
217858_s_at	ARMCX3	0.016921	0.00040119	0.0015381
209946_at	VEGFC	0.01692	0.00040125	0.0015418
217823_s_at	UBE2J1	0.016918	0.0004023	0.0015454
210222_s_at	RTN1	0.016887	0.00041363	0.0015491
204683_at	ICAM2	0.016884	0.00041502	0.0015527
222435_s_at	UBE2J1	0.016872	0.00041934	0.0015564
201236_s_at	BTG2	0.016868	0.00042115	0.0015601
227195_at	ZNF503	0.016866	0.00042173	0.0015637
204628_s_at	ITGB3	0.016838	0.00043269	0.0015674
226911_at	EGFLAM	0.016827	0.00043737	0.0015711
215071_s_at	AL353759	0.016824	0.00043855	0.0015747
205943_at	TDO2	0.016803	0.00044705	0.0015784
223169_s_at	RHOU	0.016792	0.00045143	0.001582
233177_s_at	PNKD	0.01679	0.00045235	0.0015857
200794_x_at	DAZAP2	0.016756	0.00046655	0.0015894
218989_x_at	SLC30A5	0.01674	0.00047335	0.001593
204223_at	PRELP	0.016713	0.00048509	0.0015967
225387_at	AA059445	0.01671	0.00048624	0.0016004

223577_x_at	MALAT1	0.016704	0.00048925	0.001604
227062_at	TncRNA	0.016703	0.0004896	0.0016077
205609_at	ANGPT1	0.016698	0.00049191	0.0016113
205390_s_at	ANK1	0.016691	0.00049488	0.001615
1553169_at	LRRN4	0.016674	0.00050261	0.0016187
1559477_s_at	MEIS1	0.016655	0.00051108	0.0016223
230391_at	BF439449	0.016634	0.00052127	0.001626
213001_at	ANGPTL2	0.016627	0.00052452	0.0016296
216894_x_at	CDKN1C	0.016625	0.00052515	0.0016333
225133_at	KLF3	0.016619	0.00052828	0.001637
223320_s_at	ABCB10	0.01661	0.00053262	0.0016406
216054_x_at	MYL4	0.016593	0.0005407	0.0016443
204917_s_at	MLLT3	0.016582	0.00054622	0.001648
204364_s_at	REEP1	0.016543	0.00056593	0.0016516
211709_s_at	CLEC11A	0.016496	0.00058997	0.0016553
205270_s_at	LCP2	0.016493	0.00059166	0.0016589
205534_at	PCDH7	0.016491	0.00059271	0.0016626
204537_s_at	GABRE	0.016469	0.00060463	0.0016663
213413_at	GTF2A1L	0.016443	0.00061854	0.0016699
225369_at	ESAM	0.016442	0.00061943	0.0016736
239624_at	AA725362	0.016436	0.0006227	0.0016773
227099_s_at	LOC387763	0.016433	0.00062441	0.0016809
232458_at	COL3A1	0.016426	0.00062843	0.0016846
234980_at	TMEM56	0.016398	0.00064426	0.0016882
205932_s_at	MSX1	0.016394	0.0006464	0.0016919
209220_at	GPC3	0.016392	0.0006475	0.0016956
1555950_a_at	CA448665	0.016352	0.00067134	0.0016992
201302_at	ANXA4	0.016322	0.00068913	0.0017029
213030_s_at	PLXNA2	0.01632	0.00069052	0.0017066
206493_at	ITGA2B	0.016317	0.00069229	0.0017102
204172_at	CPOX	0.016316	0.00069313	0.0017139
225224_at	ASXL1	0.016301	0.00070194	0.0017175
213275_x_at	W47179	0.016292	0.00070767	0.0017212
209357_at	CITED2	0.016277	0.00071731	0.0017249
225129_at	CPNE2	0.016266	0.00072402	0.0017285
220319_s_at	MYLIP	0.016253	0.00073257	0.0017322
218048_at	COMMD3	0.016248	0.00073598	0.0017359
225415_at	DTX3L	0.016233	0.0007459	0.0017395
223614_at	C8orf57	0.016226	0.00075034	0.0017432
202729_s_at	LTBP1	0.016157	0.00079745	0.0017468
39248_at	AQP3	0.01615	0.00080241	0.0017505
200742_s_at	TPP1	0.016141	0.00080849	0.0017542
210980_s_at	ASAH1	0.016122	0.00082201	0.0017578
205608_s_at	ANGPT1	0.016114	0.00082825	0.0017615

209292_at	ID4	0.016105	0.00083437	0.0017651
203041_s_at	LAMP2	0.016079	0.00085381	0.0017688
220751_s_at	C5orf4	0.016056	0.00087098	0.0017725
226875_at	DOCK11	0.016055	0.00087142	0.0017761
225368_at	HIPK2	0.016053	0.0008734	0.0017798
225626_at	PAG1	0.016046	0.00087839	0.0017835
213782_s_at	MYOZ2	0.016033	0.00088834	0.0017871
202283_at	SERPINF1	0.016031	0.00089038	0.0017908
218872_at	TESC	0.015958	0.0009482	0.0017944
225721_at	SYNPO2	0.015919	0.00098078	0.0017981
219905_at	ERMAP	0.015916	0.00098411	0.0018018
226899_at	UNC5B	0.015913	0.00098637	0.0018054
232432_s_at	SLC30A5	0.015911	0.00098789	0.0018091
211748_x_at	PTGDS	0.015907	0.00099155	0.0018128
219148_at	PBK	0.015899	0.00099833	0.0018164
204779_s_at	HOXB7	0.015891	0.00100484	0.0018201
223553_s_at	DOK3	0.015888	0.00100756	0.0018237
207057_at	SLC16A7	0.015862	0.00103079	0.0018274
1555419_a_at	ASAH1	0.01583	0.0010599	0.0018311
209651_at	TGFB1I1	0.015824	0.00106469	0.0018347
207148_x_at	MYOZ2	0.015801	0.00108607	0.0018384
212334_at	BE880245	0.015791	0.00109531	0.0018421
240157_at	AV694053	0.01576	0.00112499	0.0018457
221529_s_at	PLVAP	0.015752	0.00113327	0.0018494
1568597_at	LOC646762	0.015741	0.00114395	0.001853
209398_at	HIST1H1C	0.015707	0.00117717	0.0018567
222437_s_at	VPS24	0.015686	0.00119842	0.0018604
202888_s_at	ANPEP	0.015662	0.00122358	0.001864
59644_at	BMP2K	0.015654	0.00123186	0.0018677
200665_s_at	SPARC	0.015653	0.00123271	0.0018713
202833_s_at	SERPINA1	0.015634	0.00125312	0.001875
226811_at	FAM46C	0.015628	0.00125879	0.0018787
201110_s_at	THBS1	0.015615	0.00127338	0.0018823
229178_at	LOC145786	0.015608	0.00128082	0.001886
212646_at	RFTN1	0.015604	0.00128521	0.0018897
1555590_a_at	GATA1	0.015588	0.00130261	0.0018933
227558_at	CBX4	0.015561	0.0013325	0.001897
225912_at	TP53INP1	0.01556	0.00133369	0.0019006
230179_at	LOC285812	0.015554	0.00134068	0.0019043
213994_s_at	SPON1	0.015545	0.00135133	0.001908
208926_at	NEU1	0.015532	0.00136644	0.0019116
221942_s_at	GUCY1A3	0.015498	0.0014056	0.0019153
211429_s_at	SERPINA1	0.015489	0.0014164	0.001919
226460_at	FNIP2	0.015489	0.00141651	0.0019226

209890_at	TSPAN5	0.015479	0.00142892	0.0019263
209070_s_at	RGS5	0.015475	0.00143288	0.0019299
227626_at	PAQR8	0.015471	0.00143802	0.0019336
207414_s_at	PCSK6	0.015468	0.00144114	0.0019373
203131_at	PDGFRA	0.015433	0.00148517	0.0019409
225239_at	AI355441	0.015403	0.00152261	0.0019446
225116_at	HIPK2	0.015402	0.00152371	0.0019483
212418_at	ELF1	0.015399	0.0015282	0.0019519
203642_s_at	COBLL1	0.015394	0.00153415	0.0019556
227719_at	AA934610	0.015389	0.00154033	0.0019592
208937_s_at	ID1	0.015377	0.00155561	0.0019629
201301_s_at	ANXA4	0.01537	0.00156555	0.0019666
209392_at	ENPP2	0.015353	0.00158756	0.0019702
227289_at	PCDH17	0.015344	0.00159975	0.0019739
229288_at	BF439579	0.0153	0.00165948	0.0019776
212136_at	ATP2B4	0.015293	0.00166901	0.0019812
226152_at	BE963437	0.015281	0.0016852	0.0019849
209774_x_at	CXCL2	0.015251	0.00172781	0.0019885
218175_at	CCDC92	0.015237	0.00174824	0.0019922
221558_s_at	LEF1	0.015235	0.00175058	0.0019959
201009_s_at	AI439556	0.015229	0.00175916	0.0019995
223669_at	AF130060	0.015179	0.0018345	0.0020032
224690_at	C20orf108	0.015174	0.00184169	0.0020068
202656_s_at	SERTAD2	0.015168	0.00185148	0.0020105
218559_s_at	MAFB	0.015164	0.00185651	0.0020142
206414_s_at	DDEF2	0.015162	0.00185922	0.0020178
213113_s_at	SLC43A3	0.015161	0.00186212	0.0020215
236300_at	BF698797	0.015105	0.00194891	0.0020252
202506_at	SSFA2	0.015093	0.00196812	0.0020288
204940_at	PLN	0.015073	0.00200058	0.0020325
205177_at	TNNI1	0.01506	0.00202277	0.0020361
