# Supplementary material for: Estimating the Quality of Reprogrammed Cells Using ES Cell Differentiation Expression Patterns
Source: PLoS One. 2011 Jan 11;6(1):e15336. doi: 10.1371/journal.pone.0015336 (PMC3023460; doi:10.1371/journal.pone.0015336)
Supplement: Table S14 — Negative regulated genes in ES cell-derived blast cell differentiation (GSE8884). (PDF) [file pone.0015336.s017.pdf]

Table S14 Negative regulated genes in ES cell-derived blast cell differentiation (GSE8884)(224 transcript)

Probe Set_ID	Gene Name	Weight	P-value	FDR<0.1
231381_at	HESRG	-0.05797673	9.84E-41	3.66E-06
1569023_a_at	BC020935	-0.052786012	6.64E-34	7.32E-06
219955_at	L1TD1	-0.048111836	2.66E-28	1.10E-05
231061_at	AI671581	-0.044656179	1.71E-24	1.46E-05
214240_at	GAL	-0.044540742	2.27E-24	1.83E-05
229800_at	DCLK1	-0.044389049	3.29E-24	2.20E-05
237275_at	AI950472	-0.044266918	4.42E-24	2.56E-05
206224_at	CST1	-0.042610345	2.27E-22	2.93E-05
221378_at	CER1	-0.042536084	2.70E-22	3.30E-05
228038_at	SOX2	-0.042312259	4.53E-22	3.66E-05
237911_at	BF057809	-0.04158079	2.43E-21	4.03E-05
206012_at	LEFTY2	-0.041250098	5.14E-21	4.39E-05
223642_at	ZIC2	-0.040378501	3.60E-20	4.76E-05
220053_at	GDF3	-0.03845307	2.30E-18	5.13E-05
206286_s_at	TDGF1	-0.038207	3.85E-18	5.49E-05
205350_at	CRABP1	-0.037932527	6.81E-18	5.86E-05
220184_at	NANOG	-0.037252358	2.76E-17	6.23E-05
214023_x_at	TUBB2B	-0.035690909	6.24E-16	6.59E-05
223122_s_at	SFRP2	-0.034556138	5.53E-15	6.96E-05
229724_at	GABRB3	-0.034339199	8.33E-15	7.32E-05
206268_at	LEFTY1	-0.033994895	1.59E-14	7.69E-05
243610_at	C9orf135	-0.033383418	4.90E-14	8.06E-05
207197_at	ZIC3	-0.033236291	6.40E-14	8.42E-05
235343_at	AI961235	-0.032570205	2.13E-13	8.79E-05
223121_s_at	SFRP2	-0.032547106	2.22E-13	9.16E-05
206653_at	POLR3G	-0.032289805	3.50E-13	9.52E-05
227997_at	IL17RD	-0.032057938	5.26E-13	9.89E-05
210265_x_at	POU5F1P3	-0.03182471	7.92E-13	0.00010254
230356_at	AW014743	-0.031607047	1.16E-12	0.000106202
208286_x_at	POU5F1	-0.031406354	1.63E-12	0.000109864
206424_at	CYP26A1	-0.031383606	1.70E-12	0.000113526
205206_at	KAL1	-0.031361033	1.77E-12	0.000117188
205399_at	DCLK1	-0.030750679	4.98E-12	0.00012085
237193_s_at	AI435590	-0.030747096	5.01E-12	0.000124512
204469_at	PTPRZ1	-0.030701962	5.41E-12	0.000128175
237192_at	AI435590	-0.030440049	8.39E-12	0.000131837
1564413_at	FLJ36116	-0.030438612	8.41E-12	0.000135499
1554007_at	BC036488	-0.030318191	1.03E-11	0.000139161

230195_at	LOC100131138	-0.03025757	1.14E-11	0.000142823
215145_s_at	CNTNAP2	-0.030200761	1.25E-11	0.000146485
231776_at	EOMES	-0.029604294	3.31E-11	0.000150147
227830_at	GABRB3	-0.029217219	6.18E-11	0.00015381
219300_s_at	CNTNAP2	-0.028812327	1.18E-10	0.000157472
226847_at	FST	-0.02858802	1.67E-10	0.000161134
206291_at	NTS	-0.028507437	1.90E-10	0.000164796
1553874_a_at	ZSCAN10	-0.027899953	4.85E-10	0.000168458
203448_s_at	TERF1	-0.027817352	5.51E-10	0.00017212
232985_s_at	DPPA4	-0.027494879	8.97E-10	0.000175782
206023_at	NMU	-0.027115988	1.58E-09	0.000179444
222662_at	W60806	-0.026564437	3.56E-09	0.000183107
204891_s_at	LCK	-0.026445869	4.23E-09	0.000186769
219651_at	DPPA4	-0.026310691	5.14E-09	0.000190431
208939_at	SEPHS1	-0.026216212	5.89E-09	0.000194093
204984_at	GPC4	-0.026098021	6.97E-09	0.000197755
1559410_at	AA524609	-0.025575126	1.46E-08	0.000201417
227760_at	IGFBPL1	-0.025514875	1.59E-08	0.000205079
231407_s_at	FOXH1	-0.025444623	1.75E-08	0.000208742
204948_s_at	FST	-0.025117666	2.76E-08	0.000212404
212097_at	CAV1	-0.025080632	2.90E-08	0.000216066
230423_at	AI554075	-0.025071659	2.94E-08	0.000219728
224048_at	USP44	-0.025032242	3.10E-08	0.00022339
219740_at	VASH2	-0.024889681	3.77E-08	0.000227052
227690_at	GABRB3	-0.024885225	3.79E-08	0.000230714
231460_at	AI702438	-0.024728972	4.69E-08	0.000234376
201645_at	TNC	-0.024705805	4.84E-08	0.000238039
229661_at	SALL4	-0.024561866	5.88E-08	0.000241701
205603_s_at	DIAPH2	-0.024547163	6.00E-08	0.000245363
208940_at	SEPHS1	-0.024469227	6.66E-08	0.000249025
204271_s_at	EDNRB	-0.024459577	6.75E-08	0.000252687
230597_at	SLC7A3	-0.02425875	8.82E-08	0.000256349
231310_at	BF057073	-0.024211109	9.40E-08	0.000260011
205627_at	CDA	-0.024192418	9.63E-08	0.000263673
220668_s_at	DNMT3B	-0.024150162	1.02E-07	0.000267336
243161_x_at	N32798	-0.023897503	1.42E-07	0.000270998
1568609_s_at	AL118843	-0.023863711	1.48E-07	0.00027466
219301_s_at	CNTNAP2	-0.023857587	1.50E-07	0.000278322
206994_at	CST4	-0.02380355	1.61E-07	0.000281984
223449_at	SEMA6A	-0.023792807	1.63E-07	0.000285646
203453_at	SCNN1A	-0.023750989	1.72E-07	0.000289308

210905_x_at	POU5F1P4	-0.023737077	1.75E-07	0.000292971
227475_at	FOXQ1	-0.023721357	1.79E-07	0.000296633
223062_s_at	PSAT1	-0.023479009	2.45E-07	0.000300295
242128_at	OTX2	-0.023335282	2.94E-07	0.000303957
1559280_a_at	AA483467	-0.023089442	4.02E-07	0.000307619
230916_at	NODAL	-0.022935212	4.88E-07	0.000311281
230067_at	FAM124A	-0.022799344	5.79E-07	0.000314943
206701_x_at	EDNRB	-0.02264461	7.02E-07	0.000318605
209583_s_at	CD200	-0.022475625	8.65E-07	0.000322268
209372_x_at	TUBB2A	-0.022395247	9.55E-07	0.00032593
205286_at	TFAP2C	-0.022300859	1.07E-06	0.000329592
216405_at	M14087	-0.022218435	1.19E-06	0.000333254
205850_s_at	GABRB3	-0.022145542	1.30E-06	0.000336916
213629_x_at	MT1F	-0.022135654	1.31E-06	0.000340578
225958_at	PHC1	-0.022008455	1.53E-06	0.00034424
214396_s_at	MBD2	-0.021719957	2.16E-06	0.000347903
214397_at	MBD2	-0.021640065	2.38E-06	0.000351565
235845_at	SP5	-0.021595131	2.51E-06	0.000355227
204983_s_at	GPC4	-0.021583193	2.54E-06	0.000358889
219302_s_at	CNTNAP2	-0.02153023	2.71E-06	0.000362551
204141_at	TUBB2A	-0.021527842	2.71E-06	0.000366213
229518_at	FAM46B	-0.02150145	2.80E-06	0.000369875
228415_at	AA205444	-0.021472784	2.90E-06	0.000373537
225660_at	W92748	-0.021454714	2.96E-06	0.0003772
214532_x_at	POU5F1P1	-0.021403866	3.14E-06	0.000380862
226926_at	DMKN	-0.021365084	3.29E-06	0.000384524
242890_at	AI650364	-0.021193762	4.01E-06	0.000388186
226452_at	PDK1	-0.021173264	4.11E-06	0.000391848
203449_s_at	TERF1	-0.021037783	4.81E-06	0.00039551
204422_s_at	FGF2	-0.020956894	5.28E-06	0.000399172
205110_s_at	FGF13	-0.020870548	5.83E-06	0.000402834
1569287_at	BC017942	-0.020833635	6.08E-06	0.000406497
206801_at	NPPB	-0.020761539	6.60E-06	0.000410159
1570266_x_at	BC015108	-0.020691281	7.15E-06	0.000413821
223075_s_at	C9orf58	-0.020584175	8.07E-06	0.000417483
225846_at	RBM35A	-0.020551501	8.37E-06	0.000421145
225342_at	AK3L1	-0.020522276	8.65E-06	0.000424807
232111_at	LOC730125	-0.020519411	8.68E-06	0.000428469
210524_x_at	AF078844	-0.020513016	8.74E-06	0.000432132
228010_at	PPP2R2C	-0.020452908	9.35E-06	0.000435794
228906_at	TET1	-0.020404939	9.87E-06	0.000439456

222906_at	FLVCR1	-0.020276618	1.14E-05	0.000443118
228462_at	IRX2	-0.020235728	1.19E-05	0.00044678
213484_at	AI097640	-0.02022636	1.20E-05	0.000450442
220454_s_at	SEMA6A	-0.02014015	1.32E-05	0.000454104
226587_at	BE783065	-0.020031199	1.49E-05	0.000457766
213131_at	OLFM1	-0.02001267	1.52E-05	0.000461429
230543_at	USP9X	-0.019911324	1.70E-05	0.000465091
225627_s_at	CACHD1	-0.019906648	1.71E-05	0.000468753
208941_s_at	SEPHS1	-0.019884536	1.75E-05	0.000472415
217165_x_at	MT1F	-0.01988186	1.76E-05	0.000476077
214071_at	MPPE1	-0.01985242	1.82E-05	0.000479739
223541_at	HAS3	-0.019811005	1.90E-05	0.000483401
219121_s_at	RBM35A	-0.01974155	2.05E-05	0.000487064
225314_at	BG291649	-0.019677664	2.19E-05	0.000490726
209169_at	GPM6B	-0.019517639	2.61E-05	0.000494388
226482_s_at	hCG_20857	-0.019501213	2.65E-05	0.00049805
214974_x_at	CXCL5	-0.019485907	2.70E-05	0.000501712
203130_s_at	KIF5C	-0.019476135	2.72E-05	0.000505374
201601_x_at	IFITM1	-0.019458175	2.78E-05	0.000509036
237974_at	ABHD12B	-0.019455182	2.79E-05	0.000512698
240987_at	AW196940	-0.019329091	3.19E-05	0.000516361
227785_at	SDCCAG8	-0.019309896	3.25E-05	0.000520023
232235_at	DSEL	-0.019306541	3.26E-05	0.000523685
210381_s_at	CCKBR	-0.019295655	3.30E-05	0.000527347
227920_at	KIAA1553	-0.01929312	3.31E-05	0.000531009
213747_at	AZIN1	-0.019236164	3.51E-05	0.000534671
223385_at	CYP2S1	-0.019180055	3.73E-05	0.000538333
204326_x_at	MT1L	-0.019177848	3.74E-05	0.000541995
207644_at	FOXH1	-0.019157132	3.82E-05	0.000545658
221245_s_at	NM_030804	-0.019117541	3.98E-05	0.00054932
206783_at	BF510715	-0.019088025	4.11E-05	0.000552982
217546_at	MT1M	-0.019062319	4.22E-05	0.000556644
200862_at	DHCR24	-0.019017884	4.42E-05	0.000560306
227769_at	AI703476	-0.019007903	4.46E-05	0.000563968
201968_s_at	PGM1	-0.018980879	4.59E-05	0.00056763
216319_at	AK022686	-0.018869216	5.16E-05	0.000571293
212560_at	SORL1	-0.018781756	5.64E-05	0.000574955
213721_at	SOX2	-0.018744471	5.86E-05	0.000578617
209168_at	GPM6B	-0.01868391	6.24E-05	0.000582279
220892_s_at	PSAT1	-0.018671855	6.32E-05	0.000585941
219823_at	LIN28	-0.018560472	7.08E-05	0.000589603

204944_at	PTPRG	-0.018442948	7.98E-05	0.000593265
239752_at	BE551781	-0.018420837	8.16E-05	0.000596927
226834_at	BG112263	-0.018350146	8.76E-05	0.00060059
212859_x_at	MT1E	-0.018340918	8.84E-05	0.000604252
226591_at	BG413612	-0.018209799	0.000100832	0.000607914
228955_at	AL041761	-0.018130642	0.000109109	0.000611576
218683_at	PTBP2	-0.018070073	0.000115872	0.000615238
227496_at	LOC253842	-0.017994314	0.000124889	0.0006189
204836_at	GLDC	-0.017900491	0.000136975	0.000622562
232060_at	AK000776	-0.017798011	0.000151433	0.000626225
210544_s_at	ALDH3A2	-0.017796951	0.00015159	0.000629887
211596_s_at	LRIG1	-0.017783383	0.000153611	0.000633549
205478_at	PPP1R1A	-0.017770311	0.000155581	0.000637211
206117_at	TPM1	-0.017753961	0.00015808	0.000640873
208555_x_at	CST2	-0.017745022	0.000159462	0.000644535
218338_at	PHC1	-0.0177165	0.000163948	0.000648197
1552670_a_at	PPP1R3B	-0.01765462	0.000174093	0.000651859
236519_at	C9orf135	-0.017653295	0.000174316	0.000655522
230788_at	GCNT2	-0.017651665	0.000174592	0.000659184
221805_at	NEFL	-0.017614657	0.000180955	0.000662846
205268_s_at	ADD2	-0.017576764	0.000187697	0.000666508
214022_s_at	IFITM1	-0.017562593	0.000190278	0.00067017
209167_at	AI419030	-0.017553775	0.000191902	0.000673832
207345_at	FST	-0.017503161	0.000201473	0.000677494
229456_s_at	AI885718	-0.017497849	0.000202503	0.000681157
209170_s_at	AF016004	-0.017464814	0.000209022	0.000684819
202054_s_at	ALDH3A2	-0.017427641	0.000216593	0.000688481
203065_s_at	CAV1	-0.017289784	0.000246981	0.000692143
242346_x_at	BF222929	-0.017257867	0.000254566	0.000695805
229202_at	AI768826	-0.01723529	0.000260063	0.000699467
204348_s_at	AK3L1	-0.017213126	0.000265568	0.000703129
230493_at	SHISA2	-0.017071402	0.000303436	0.000706791
1553991_s_at	FLJ20674	-0.017070976	0.000303557	0.000710454
223038_s_at	FAM60A	-0.017015977	0.000319578	0.000714116
205805_s_at	ROR1	-0.016997138	0.000325246	0.000717778
228120_at	AW136032	-0.016907262	0.000353606	0.00072144
213709_at	H11725	-0.016893186	0.000358251	0.000725102
237896_at	NODAL	-0.016880662	0.000362433	0.000728764
227350_at	AI889959	-0.016796465	0.000391754	0.000732426
221974_at	AW770748	-0.016767931	0.000402185	0.000736088
208581_x_at	MT1X	-0.01676505	0.000403252	0.000739751

202712_s_at	CKMT1B	-0.016752276	0.000408017	0.000743413
204745_x_at	MT1G	-0.016716663	0.000421581	0.000747075
1570153_at	SOHLH2	-0.01670654	0.000425513	0.000750737
208712_at	CCND1	-0.016698992	0.000428467	0.000754399
206461_x_at	MT1H	-0.016686547	0.000433379	0.000758061
213880_at	LGR5	-0.016652145	0.000447234	0.000761723
220085_at	HELLS	-0.016634256	0.000454601	0.000765386
243672_at	SALL3	-0.016623051	0.000459272	0.000769048
206243_at	TIMP4	-0.016555785	0.000488275	0.00077271
206541_at	KLKB1	-0.016542314	0.000494286	0.000776372
204471_at	GAP43	-0.016539015	0.000495768	0.000780034
204347_at	AI653169	-0.016529032	0.000500279	0.000783696
223234_at	MAD2L2	-0.016498227	0.000514441	0.000787358
223761_at	FGF19	-0.016475834	0.00052497	0.00079102
237862_at	AW590614	-0.016454911	0.00053499	0.000794683
230785_at	AI634411	-0.016364039	0.000580611	0.000798345
221916_at	NEFL	-0.016325528	0.000601018	0.000802007
230372_at	HAS2	-0.016305203	0.000612055	0.000805669
214823_at	ZNF204	-0.016271816	0.000630598	0.000809331
216836_s_at	ERBB2	-0.016143709	0.00070671	0.000812993
229700_at	BE966267	-0.016049711	0.000767902	0.000816655
209582_s_at	CD200	-0.016017106	0.000790253	0.000820318
