# Supplementary material for: Estimating the Quality of Reprogrammed Cells Using ES Cell Differentiation Expression Patterns
Source: PLoS One. 2011 Jan 11;6(1):e15336. doi: 10.1371/journal.pone.0015336 (PMC3023460; doi:10.1371/journal.pone.0015336)
Supplement: Table S15 — Positive regulated genes in ES cells-derived neuron rosette differentiation (GSE9940). (PDF) [file pone.0015336.s018.pdf]

Table S15 Positive regulated genes in ES cells-derived neuron rosette differentiation (GSE9940) (135 transcripts)

Probe Set_ID	Gene Name	Weight	P-value	FDR<0.1
217404_s_at	COL2A1	0.0346827	1.47E-15	3.66E-06
206140_at	LHX2	0.031200251	9.04E-13	7.32E-06
206373_at	ZIC1	0.029373319	2.01E-11	1.10E-05
206163_at	MAB21L1	0.029120326	3.04E-11	1.46E-05
239230_at	HES5	0.027276806	5.63E-10	1.83E-05
230882_at	AA129217	0.027044524	8.02E-10	2.20E-05
211219_s_at	LHX2	0.026726266	1.30E-09	2.56E-05
205337_at	TGDS	0.026546003	1.70E-09	2.93E-05
204818_at	HSD17B2	0.026022897	3.68E-09	3.30E-05
235795_at	PAX6	0.025953074	4.07E-09	3.66E-05
227498_at	AI480314	0.02573449	5.59E-09	4.03E-05
228854_at	AI492388	0.025649371	6.32E-09	4.39E-05
205612_at	MMRN1	0.025607799	6.71E-09	4.76E-05
233257_at	AU148032	0.025423319	8.75E-09	5.13E-05
201744_s_at	LUM	0.025357732	9.60E-09	5.49E-05
207443_at	NR2E1	0.025254107	1.11E-08	5.86E-05
206018_at	FOXG1	0.025203388	1.20E-08	6.23E-05
237472_at	SOX1	0.02447387	3.31E-08	6.59E-05
229290_at	DAPL1	0.024390607	3.72E-08	6.96E-05
233972_s_at	FEZF2	0.024309412	4.15E-08	7.32E-05
240713_s_at	AI697836	0.024109063	5.45E-08	7.69E-05
210875_s_at	ZEB1	0.02407507	5.71E-08	8.06E-05
230184_at	AL035834	0.023974702	6.54E-08	8.42E-05
230865_at	LIX1	0.023686677	9.63E-08	8.79E-05
238877_at	BE674583	0.023662779	9.94E-08	9.16E-05
213921_at	SST	0.02357887	1.11E-07	9.52E-05
207250_at	SIX6	0.023385744	1.43E-07	9.89E-05
221950_at	EMX2	0.022636468	3.79E-07	0.00010254
243561_at	AA651631	0.022552399	4.22E-07	0.000106202
230272_at	LOC645323	0.0225271	4.35E-07	0.000109864
212764_at	AI806174	0.022465204	4.71E-07	0.000113526
227347_x_at	HES4	0.022060108	7.83E-07	0.000117188
228780_at	AW149422	0.021948647	8.99E-07	0.00012085
243061_at	C14orf23	0.021904417	9.49E-07	0.000124512
229435_at	GLIS3	0.021717046	1.20E-06	0.000128175
209505_at	NR2F1	0.02165739	1.29E-06	0.000131837
209560_s_at	DLK1	0.02160643	1.37E-06	0.000135499
236896_at	ZIC1	0.021545555	1.47E-06	0.000139161
209987_s_at	ASCL1	0.02128723	2.01E-06	0.000142823
207147_at	DLX2	0.021159222	2.35E-06	0.000146485
238850_at	LOC645323	0.021106155	2.50E-06	0.000150147
213492_at	COL2A1	0.021066116	2.62E-06	0.00015381
1557545_s_at	RNF165	0.020999115	2.84E-06	0.000157472

210179_at	KCNJ13	0.020998753	2.84E-06	0.000161134
229281_at	N51682	0.020881721	3.26E-06	0.000164796
212070_at	GPR56	0.020708727	3.99E-06	0.000168458
1552388_at	NM_152514	0.020542109	4.85E-06	0.00017212
206070_s_at	EPHA3	0.020502446	5.08E-06	0.000175782
223204_at	C4orf18	0.020374621	5.88E-06	0.000179444
227235_at	AI758408	0.020286012	6.51E-06	0.000183107
238914_at	AI962169	0.020240361	6.86E-06	0.000186769
236163_at	LIX1	0.020224387	6.99E-06	0.000190431
228943_at	MAP6	0.020190337	7.27E-06	0.000194093
209988_s_at	ASCL1	0.020136102	7.73E-06	0.000197755
207658_s_at	NM_004471	0.020024697	8.77E-06	0.000201417
242715_at	AA331548	0.019848582	1.07E-05	0.000205079
205030_at	FABP7	0.019667159	1.31E-05	0.000208742
205029_s_at	FABP7	0.019454534	1.66E-05	0.000212404
232204_at	EBF1	0.019350521	1.86E-05	0.000216066
204851_s_at	DCX	0.019274613	2.02E-05	0.000219728
214183_s_at	TKTL1	0.019270321	2.03E-05	0.00022339
228915_at	DACH1	0.019267748	2.03E-05	0.000227052
213880_at	LGR5	0.01925497	2.06E-05	0.000230714
241440_at	FLJ30375	0.019215628	2.15E-05	0.000234376
209074_s_at	FAM107A	0.019195206	2.20E-05	0.000238039
232424_at	PRDM16	0.019133628	2.35E-05	0.000241701
221029_s_at	WNT5B	0.019086011	2.47E-05	0.000245363
225540_at	MAP2	0.019070708	2.52E-05	0.000249025
230547_at	R52825	0.018906748	3.00E-05	0.000252687
227082_at	AI760356	0.018904023	3.01E-05	0.000256349
226535_at	ITGB6	0.018845849	3.20E-05	0.000260011
211024_s_at	NKX2-1	0.018819428	3.29E-05	0.000263673
209469_at	GPM6A	0.018742417	3.57E-05	0.000267336
228979_at	SFTPH	0.018631006	4.02E-05	0.000270998
203000_at	BF967657	0.018580018	4.24E-05	0.00027466
1558170_at	BG746017	0.018544057	4.41E-05	0.000278322
206282_at	NEUROD1	0.018469395	4.76E-05	0.000281984
205646_s_at	PAX6	0.018450979	4.86E-05	0.000285646
203001_s_at	STMN2	0.018409671	5.07E-05	0.000289308
212224_at	ALDH1A1	0.018377802	5.24E-05	0.000292971
209470_s_at	GPM6A	0.018319595	5.57E-05	0.000296633
238878_at	ARX	0.018317548	5.58E-05	0.000300295
214803_at	BF344237	0.018115693	6.87E-05	0.000303957
206349_at	LGI1	0.018104836	6.95E-05	0.000307619
226552_at	IER5L	0.018011019	7.65E-05	0.000311281
206982_at	CRYBA1	0.017824438	9.24E-05	0.000314943
221086_s_at	FEZF2	0.017817516	9.30E-05	0.000318605
211161_s_at	AF130082	0.017816696	9.31E-05	0.000322268
230290_at	SCUBE3	0.01778669	9.60E-05	0.00032593

227762_at	AW244016	0.017684478	0.000106358	0.000329592
227646_at	EBF1	0.017511921	0.000126324	0.000333254
242138_at	DLX1	0.017490422	0.000129045	0.000336916
210729_at	NPY2R	0.017453251	0.000133883	0.000340578
1566163_at	AL832482	0.01744418	0.000135089	0.00034424
212758_s_at	ZEB1	0.017417233	0.000138732	0.000347903
204850_s_at	DCX	0.017247826	0.00016385	0.000351565
230412_at	NPAS3	0.017230997	0.000166567	0.000355227
207672_at	RFX4	0.017209291	0.000170134	0.000358889
229715_at	AW006182	0.017187737	0.000173746	0.000362551
218870_at	ARHGAP15	0.01714009	0.000181991	0.000366213
230130_at	AI692523	0.017107382	0.000187862	0.000369875
238073_at	ELAVL4	0.01699963	0.00020849	0.000373537
202410_x_at	IGF2	0.016896283	0.00023026	0.0003772
207336_at	SOX5	0.016894002	0.000230764	0.000380862
202833_s_at	SERPINA1	0.016892982	0.00023099	0.000384524
223673_at	RFX4	0.016879327	0.00023403	0.000388186
210432_s_at	SCN3A	0.01681748	0.000248278	0.000391848
223537_s_at	WNT5B	0.016786341	0.000255756	0.00039551
219895_at	FAM70A	0.016785907	0.000255861	0.000399172
235888_at	AI873678	0.016759747	0.000262309	0.000402834
203030_s_at	PTPRN2	0.016748483	0.000265132	0.000406497
204424_s_at	LMO3	0.016710265	0.000274924	0.000410159
227666_at	DCLK2	0.016673706	0.000284608	0.000413821
229580_at	R71596	0.016666675	0.000286507	0.000417483
231032_at	BE503158	0.016645456	0.00029231	0.000421145
229645_at	H14197	0.016632079	0.000296024	0.000424807
206622_at	TRH	0.016615031	0.000300822	0.000428469
206634_at	SIX3	0.016611342	0.00030187	0.000432132
207018_s_at	RAB27B	0.016607972	0.00030283	0.000435794
207480_s_at	NM_020149	0.016603165	0.000304205	0.000439456
226814_at	ADAMTS9	0.016538641	0.000323231	0.000443118
207195_at	CNTN6	0.016518038	0.000329538	0.00044678
212843_at	NCAM1	0.016515977	0.000330175	0.000450442
205883_at	ZBTB16	0.016483732	0.000340295	0.000454104
236360_at	FLJ42875	0.016457246	0.000348824	0.000457766
236563_at	RD3	0.016416322	0.000362398	0.000461429
1553614_a_at	NM_173604	0.016407091	0.000365527	0.000465091
228720_at	SORCS2	0.016267419	0.000416073	0.000468753
214628_at	NHLH1	0.016224436	0.000432898	0.000472415
213055_at	CD47	0.016218339	0.000435336	0.000476077
244744_at	LOC100130502	0.016212293	0.000437766	0.000479739
206051_at	ELAVL4	0.016156441	0.000460819	0.000483401
205358_at	GRIA2	0.016140236	0.000467717	0.000487064
235600_at	N63890	0.016109997	0.000480847	0.000490726
229441_at	PRSS23	0.016089138	0.000490104	0.000494388
