# Supplementary material for: Estimating the Quality of Reprogrammed Cells Using ES Cell Differentiation Expression Patterns
Source: PLoS One. 2011 Jan 11;6(1):e15336. doi: 10.1371/journal.pone.0015336 (PMC3023460; doi:10.1371/journal.pone.0015336)
Supplement: Table S16 — Positive regulated genes in ES cells-derived neuron rosette differentiation (GSE9940). (PDF) [file pone.0015336.s019.pdf]

Table S16 Negative regulated genes in ES cell-derived neuron rosettes differentiation (GSE9940) (89 transcripts)

Probe Set_ID	Gene Name	Weight	P-value	FDR<0.1
1554776_at	ZFP42	-0.031307974	3.58E-12	3.66E-06
228782_at	SCGB3A2	-0.029006974	1.56E-10	7.32E-06
1559280_a_at	AA483467	-0.028878117	1.91E-10	1.10E-05
220184_at	NANOG	-0.028431731	3.83E-10	1.46E-05
205309_at	SMPDL3B	-0.028112237	6.26E-10	1.83E-05
231381_at	HESRG	-0.027642336	1.28E-09	2.20E-05
206286_s_at	TDGF1	-0.027626518	1.31E-09	2.56E-05
237192_at	AI435590	-0.027557161	1.45E-09	2.93E-05
209040_s_at	PSMB8	-0.027394473	1.85E-09	3.30E-05
210905_x_at	POU5F1P4	-0.026393797	7.97E-09	3.66E-05
1554777_at	ZFP42	-0.026081797	1.24E-08	4.03E-05
216405_at	M14087	-0.025594617	2.46E-08	4.39E-05
237193_s_at	AI435590	-0.025186339	4.32E-08	4.76E-05
231690_at	AI962352	-0.024837099	6.93E-08	5.13E-05
210029_at	INDO	-0.024780769	7.48E-08	5.49E-05
210265_x_at	POU5F1P3	-0.024650388	8.91E-08	5.86E-05
1553874_a_at	ZSCAN10	-0.024430198	1.19E-07	6.23E-05
203453_at	SCNN1A	-0.024220634	1.57E-07	6.59E-05
206002_at	GPR64	-0.023282046	5.27E-07	6.96E-05
202790_at	CLDN7	-0.023080644	6.78E-07	7.32E-05
224344_at	COX6A1	-0.022984041	7.65E-07	7.69E-05
237911_at	BF057809	-0.02281879	9.39E-07	8.06E-05
243161_x_at	N32798	-0.022797499	9.64E-07	8.42E-05
214532_x_at	POU5F1P1	-0.022790318	9.72E-07	8.79E-05
220714_at	PRDM14	-0.022673308	1.12E-06	9.16E-05
214974_x_at	CXCL5	-0.022296255	1.78E-06	9.52E-05
230195_at	LOC100131138	-0.022149293	2.12E-06	9.89E-05
203397_s_at	GALNT3	-0.021765142	3.35E-06	0.00010254
243610_at	C9orf135	-0.021352466	5.41E-06	0.000106202
206541_at	KLKB1	-0.021352212	5.41E-06	0.000109864
219955_at	L1TD1	-0.021234186	6.20E-06	0.000113526
233305_at	NECAB1	-0.021195141	6.49E-06	0.000117188
1556351_at	HCN1	-0.021176458	6.63E-06	0.00012085
206309_at	LECT1	-0.021164073	6.72E-06	0.000124512
208286_x_at	POU5F1	-0.021063844	7.54E-06	0.000128175
206268_at	LEFTY1	-0.021021099	7.91E-06	0.000131837
239781_at	hCG_1815504	-0.020958724	8.49E-06	0.000135499
1553875_s_at	ZSCAN10	-0.020933769	8.73E-06	0.000139161
220065_at	TNMD	-0.020811961	1.00E-05	0.000142823
239552_at	FLJ14712	-0.020746285	1.08E-05	0.000146485
207644_at	FOXH1	-0.020736672	1.09E-05	0.000150147
206424_at	CYP26A1	-0.020569661	1.31E-05	0.00015381
1552897_a_at	KCNG3	-0.020568361	1.32E-05	0.000157472

204891_s_at	LCK	-0.020260828	1.85E-05	0.000161134
231544_s_at	POLR3G	-0.020235041	1.90E-05	0.000164796
219121_s_at	RBM35A	-0.020224323	1.92E-05	0.000168458
226498_at	AA149648	-0.020152362	2.08E-05	0.00017212
231079_at	AW137517	-0.019996025	2.46E-05	0.000175782
220317_at	LRAT	-0.019945388	2.60E-05	0.000179444
231698_at	UGP2	-0.019905398	2.71E-05	0.000183107
231061_at	AI671581	-0.019843614	2.90E-05	0.000186769
1564083_at	AL833138	-0.019749156	3.21E-05	0.000190431
225846_at	RBM35A	-0.019700654	3.38E-05	0.000194093
231628_s_at	AW262311	-0.019635577	3.62E-05	0.000197755
214240_at	GAL	-0.01953377	4.03E-05	0.000201417
229292_at	EPB41L5	-0.019241867	5.47E-05	0.000205079
213982_s_at	RABGAP1L	-0.019175249	5.86E-05	0.000208742
203567_s_at	AU157590	-0.019068784	6.54E-05	0.000212404
202890_at	AW242297	-0.019049934	6.67E-05	0.000216066
218180_s_at	EPS8L2	-0.01892932	7.55E-05	0.000219728
221666_s_at	PYCARD	-0.018905576	7.73E-05	0.00022339
223423_at	GPR160	-0.018858862	8.11E-05	0.000227052
1553132_a_at	TC2N	-0.01878976	8.70E-05	0.000230714
230641_at	AI792670	-0.018746748	9.08E-05	0.000234376
215101_s_at	CXCL5	-0.018625724	0.000102609	0.000238039
206653_at	POLR3G	-0.018573122	0.000108164	0.000241701
239975_at	HLA-DPB2	-0.018533865	0.000112493	0.000245363
228441_s_at	BE550153	-0.018476271	0.000119142	0.000249025
239319_at	LOC728342	-0.018461085	0.000120956	0.000252687
205627_at	CDA	-0.018425895	0.00012526	0.000256349
219545_at	KCTD14	-0.018379716	0.000131128	0.000260011
1570266_x_at	BC015108	-0.018351121	0.000134891	0.000263673
201422_at	IFI30	-0.018350979	0.00013491	0.000267336
231407_s_at	FOXH1	-0.018249476	0.000149106	0.000270998
240681_at	AW118997	-0.018247812	0.00014935	0.00027466
202889_x_at	MAP7	-0.018180577	0.000159533	0.000278322
1561101_at	LOC153469	-0.018160352	0.000162722	0.000281984
215471_s_at	MAP7	-0.018130916	0.00016747	0.000285646
1569886_a_at	GLB1L3	-0.017995455	0.000191055	0.000289308
1570422_at	SNTG2	-0.017858096	0.000218137	0.000292971
229095_s_at	LIMS3	-0.017797054	0.000231297	0.000296633
236894_at	L1TD1	-0.017771182	0.000237096	0.000300295
228440_at	BE550153	-0.017753063	0.000241239	0.000303957
201131_s_at	CDH1	-0.017697174	0.00025445	0.000307619
216319_at	AK022686	-0.017688729	0.000256504	0.000311281
236058_at	C1orf172	-0.01765282	0.000265414	0.000314943
220668_s_at	DNMT3B	-0.017622347	0.000273203	0.000318605
230423_at	AI554075	-0.017606406	0.000277362	0.000322268
216171_at	AK025271	-0.017461034	0.000318132	0.00032593
