# Supplementary material for: Estimating the Quality of Reprogrammed Cells Using ES Cell Differentiation Expression Patterns
Source: PLoS One. 2011 Jan 11;6(1):e15336. doi: 10.1371/journal.pone.0015336 (PMC3023460; doi:10.1371/journal.pone.0015336)
Supplement: Table S17 — Negative regulated genes in ES cell-derived Cardiac precursors cells Differentiation (GSE10970). (PDF) [file pone.0015336.s020.pdf]

Table S17 Negative regulated genes in ES cell-derived Cardiac precursors cells Differentiation (GSE10970)

Probe Set_ID	Acc_Num	Gene Name	Weight	P-value	FDR<0.1
1429654_at	AK010743	2410088E07Rik	-0.039207386	9.78E-16	4.44E-06
1417760_at	NM_007430	Nr0b1	-0.038016178	7.71E-15	8.88E-06
1419418_a_at	NM_010816	Morc	-0.036310427	1.33E-13	1.33E-05
1449254_at	NM_009263	Spp1	-0.034301937	3.21E-12	1.78E-05
1438872_at	AV278675	Mm.64542.1	-0.031327593	2.56E-10	2.22E-05
1436837_at	AV279061	AU019877	-0.031121418	3.42E-10	2.66E-05
1456242_at	AV101904	Mm.45676.1	-0.030714213	6.02E-10	3.11E-05
1457033_at	BM198753	Mm.131747.1	-0.029997445	1.60E-09	3.55E-05
1418362_at	NM_009556	Zfp42	-0.029707736	2.35E-09	4.00E-05
1427479_at	AA413531	Eif1a	-0.029572269	2.82E-09	4.44E-05
1428640_at	AK016553	Hsf2bp	-0.029544114	2.93E-09	4.88E-05
1443961_at	BG071029	AU017962	-0.028891336	6.88E-09	5.33E-05
1427238_at	AF176530	Fbxo15	-0.028598124	1.00E-08	5.77E-05
1449288_at	NM_008108	Gdf3	-0.028385277	1.32E-08	6.22E-05
1453223_s_at	AK010743	2410088E07Rik	-0.028082103	1.94E-08	6.66E-05
1434917_at	BQ173923	Mm.22847.1	-0.027261941	5.36E-08	7.11E-05
1444529_at	BM197200	Mm.145922.1	-0.026363709	1.58E-07	7.55E-05
1457314_at	BB041571	Mm.34340.1	-0.026222644	1.86E-07	7.99E-05
1416552_at	NM_025274	Esg1	-0.026091875	2.17E-07	8.44E-05
1420085_at	BB709552	Mm.212127.1	-0.026089217	2.18E-07	8.88E-05
1431416_a_at	AK010826	Jcam2	-0.025762306	3.18E-07	9.33E-05
1425035_s_at	AF220524	Dnmt3l	-0.025381025	4.93E-07	9.77E-05
1419542_at	NM_010021	Dazl	-0.024291943	1.66E-06	0.000102138
1438237_at	BM198106	Mm.21900.1	-0.024288328	1.66E-06	0.000106579
1420086_x_at	BB709552	Mm.212127.1	-0.024197441	1.84E-06	0.00011102
1429701_at	AK010362	2410003J06Rik	-0.02391097	2.50E-06	0.000115461
1445849_at	BG070782	Mm.26025	-0.023761594	2.93E-06	0.000119901
1443844_at	AV269723	Mm.50505.1	-0.023690585	3.16E-06	0.000124342
1418091_at	NM_023755	Crtr1-pending	-0.023665258	3.25E-06	0.000128783
1460471_at	BB702364	2410146L05Rik	-0.023626277	3.39E-06	0.000133224
1437786_at	BG069933	C80008	-0.023617933	3.42E-06	0.000137665
1429388_at	AK010332	2410002E02Rik	-0.023436652	4.14E-06	0.000142105
1419758_at	M30697	Abcb1a	-0.023136359	5.66E-06	0.000146546
1444390_at	BB283625	Mm.67875.1	-0.022917999	7.09E-06	0.000150987
1449088_at	NM_007994	Fbp2	-0.022887106	7.31E-06	0.000155428
1419106_at	NM_028218	2210409E12Rik	-0.022806366	7.94E-06	0.000159869
1429525_s_at	AK021181	Myo1f	-0.02260129	9.79E-06	0.000164309
1419959_s_at	AU019881	AU019881	-0.022428376	1.17E-05	0.00016875
1449064_at	NM_021480	Tdh	-0.022404142	1.19E-05	0.000173191
1416899_at	NM_009482	Utf1	-0.022333187	1.28E-05	0.000177632
1420773_at	NM_007887	Dub1	-0.022311051	1.31E-05	0.000182073
1434025_at	BG069607	Klf5	-0.022194338	1.47E-05	0.000186513
1436568_at	AU016127	AU016127	-0.022185721	1.48E-05	0.000190954
1435154_at	AV099404	AU018091	-0.022152934	1.53E-05	0.000195395

1425220_x_at	AF067062	variable group of 2-cell-stage gene family	-0.021959312	1.86E-05	0.000199836
1442334_at	BB711873	Mm.212193.1	-0.02172603	2.33E-05	0.000204276
1426858_at	BB253137	Inhbb	-0.021582629	2.68E-05	0.000208717
1423327_at	AK005645	4930517K11Rik	-0.021388693	3.23E-05	0.000213158
1436926_at	AV333667	Esrrb	-0.021307531	3.49E-05	0.000217599
1423582_at	AL133300	Dmrt1	-0.021212069	3.82E-05	0.00022204
1460226_at	NM_011635	Trap1a	-0.02089109	5.18E-05	0.00022648
1423523_at	BF687395	Lorsdh	-0.020803599	5.62E-05	0.000230921
1418569_at	BG070068	2410043F08Rik	-0.020745982	5.92E-05	0.000235362
1449408_at	NM_023844	1110002N23Rik	-0.020633445	6.58E-05	0.000239803
1417945_at	NM_013633	Pou5f1	-0.020605359	6.75E-05	0.000244244
1449170_at	NM_021308	Piwil2	-0.020502278	7.42E-05	0.000248684
1422937_at	NM_022721	Fzd5	-0.020310906	8.84E-05	0.000253125
1452008_at	AK018685	9130422G05Rik	-0.020296944	8.95E-05	0.000257566
1451021_a_at	BI465857	Klf5	-0.019975112	0.000119611	0.000262007
1416529_at	U25633	TMP	-0.019938135	0.000123627	0.000266448
1416318_at	AF426024	Serpinb1	-0.019918851	0.000125772	0.000270888
1437015_x_at	AV060866	Pla2g1b	-0.019797293	0.000140121	0.000275329
1418395_at	NM_026183	1300013J15Rik	-0.019784247	0.000141749	0.00027977
1442384_at	BG065559	Mm.39486.1	-0.019750419	0.000146055	0.000284211
1429802_at	AK002831	0610039E24Rik	-0.019684901	0.000154748	0.000288652
1419759_at	M30697	Abcb1a	-0.019630368	0.000162352	0.000293092
1427242_at	AK014844	Ddx4	-0.019504859	0.000181212	0.000297533
1417482_at	NM_028602	Tex19	-0.019503504	0.000181427	0.000301974
1418215_at	NM_008586	Mep1b	-0.019380278	0.000201957	0.000306415
1422567_at	NM_022018	Niban	-0.019358493	0.000205806	0.000310856
1416316_at	BC013442	Slc27a2	-0.019334044	0.000210208	0.000315296
1429483_at	BG066947	2410154J16Rik	-0.019319968	0.000212783	0.000319737
1422458_at	NM_009337	Tcl1	-0.019304732	0.000215603	0.000324178
1431865_a_at	AK015213	4933405K07Rik	-0.019291354	0.000218108	0.000328619
1423378_at	AI838132	Adam23	-0.019280168	0.000220223	0.000333059
1421117_at	NM_010081	Dst	-0.019249908	0.000226043	0.0003375
1436562_at	BG063981	Mm.80701.1	-0.019157827	0.000244654	0.000341941
1429366_at	AK005720	1700007J06Rik	-0.019153772	0.000245506	0.000346382
1426808_at	X16834	Lgals3	-0.019144774	0.000247406	0.000350823
1420337_at	L39770	Gbx-2	-0.019044308	0.000269578	0.000355263
1456127_at	AV299691	Mm.68286.1	-0.019002587	0.000279323	0.000359704
1448713_at	NM_011487	Stat4	-0.018987403	0.00028295	0.000364145
1437588_at	BB547375	Mm.168942.1	-0.018962441	0.00028901	0.000368586
1452050_at	BG071931	Mm.26888.1	-0.018957003	0.000290346	0.000373027
1423281_at	BM115022	Scgn10	-0.018934854	0.000295849	0.000377467
1447997_s_at	BG066908	Mm.38540.1	-0.018878991	0.000310164	0.000381908
1449592_at	NM_009328	Tcf15	-0.01871531	0.000355932	0.000386349
1423280_at	BM115022	Scgn10	-0.018653285	0.000374871	0.00039079
1436742_a_at	BG070524	Mm.9889.1	-0.018642792	0.000378166	0.000395231
