# Supplementary material for: Estimating the Quality of Reprogrammed Cells Using ES Cell Differentiation Expression Patterns
Source: PLoS One. 2011 Jan 11;6(1):e15336. doi: 10.1371/journal.pone.0015336 (PMC3023460; doi:10.1371/journal.pone.0015336)
Supplement: Table S18 — Positive regulated genes in ES cell-derived Cardiac precursors cells Differentiation (GSE10970). (PDF) [file pone.0015336.s021.pdf]

Table S18 Positive regulated genes in ES cell-derived Cardiac precursors cells Differentiation (GSE10970)

Probe Set_ID	Acc_Num	Gene Name	Weight	P-value	FDR<0.1
1437990_x_at	AV147727	Hbb-bh1	0.04277662	7.89E-19	4.44E-06
1450736_a_at	NM_008219	Hbb-bh1	0.042209849	2.35E-18	8.88E-06
1437810_a_at	AV311770	Hbb-bh1	0.041303041	1.30E-17	1.33E-05
1436823_x_at	AV148191	Hbb-y	0.041051484	2.08E-17	1.78E-05
1436717_x_at	AV156860	Hbb-y	0.033299969	9.56E-12	2.22E-05
1448716_at	M26898	Hba-x	0.03325663	1.02E-11	2.66E-05
1438651_a_at	BB483357	Agtrl1	0.030986026	2.77E-10	3.11E-05
1436041_at	BB029192	AI661148	0.029868532	1.29E-09	3.55E-05
1428361_x_at	AK011116	Hba-a1	0.029280464	2.84E-09	4.00E-05
1433919_at	AV302111	Asb4	0.02920801	3.13E-09	4.44E-05
1452757_s_at	AK011116	Hba-a1	0.028736469	5.81E-09	4.88E-05
1454608_x_at	BG141874	Ttr	0.0285628	7.28E-09	5.33E-05
1427769_x_at	X67685	Mylc	0.028266522	1.07E-08	5.77E-05
1450813_a_at	NM_021467	Tnni1	0.02765536	2.31E-08	6.22E-05
1450621_a_at	NM_008221	Hbb-y	0.027440513	3.02E-08	6.66E-05
1417714_x_at	NM_008218	Hba-a1	0.027291822	3.64E-08	7.11E-05
1418726_a_at	NM_011619	Tnnt2	0.027028098	5.03E-08	7.55E-05
1449071_at	NM_022879	Mylc2a	0.026895521	5.92E-08	7.99E-05
1452114_s_at	BF225802	Igfbp5	0.026630268	8.16E-08	8.44E-05
1415927_at	NM_009608	Actc1	0.02657122	8.76E-08	8.88E-05
1437339_s_at	BB241731	Mm.180403.1	0.026173437	1.41E-07	9.33E-05
1448152_at	NM_010514	Igf2	0.025734869	2.36E-07	9.77E-05
1427768_s_at	X67685	Mylc	0.025273708	4.03E-07	0.000102138
1421951_at	AV335209	Lhx1	0.025272259	4.03E-07	0.000106579
1424967_x_at	L47552	Tnnt2	0.024645597	8.19E-07	0.00011102
1448696_at	NM_010417	Heph	0.023887426	1.89E-06	0.000115461
1450922_a_at	BF144658	Tgfb2	0.023248261	3.74E-06	0.000119901
1416286_at	NM_009062	Rgs4	0.023077973	4.47E-06	0.000124342
1448748_at	AF181829	Plek	0.022997964	4.86E-06	0.000128783
1434413_at	BG092677	Mm.45770.1	0.022875095	5.52E-06	0.000133224
1425978_at	AF384055	Mm.32257.2	0.022854141	5.64E-06	0.000137665
1450723_at	BQ176915	Isl1	0.022847578	5.68E-06	0.000142105
1420941_at	BF585144	Rgs5	0.022793554	6.01E-06	0.000146546
1418517_at	NM_008393	Irx3	0.022764135	6.19E-06	0.000150987
1438030_at	BB042252	Mm.38170.1	0.022448591	8.56E-06	0.000155428
1455851_at	AV032115	AU023399	0.022439936	8.63E-06	0.000159869
1418493_a_at	NM_009221	Snca	0.022343816	9.52E-06	0.000164309
1448925_at	NM_007855	Dermo1	0.02233237	9.63E-06	0.00016875
1436312_at	AV317621	Mm.39709.1	0.022175124	1.13E-05	0.000173191
1459737_s_at	AA408768	AA408768	0.022071391	1.25E-05	0.000177632
1423635_at	AV239587	Bmp2	0.02199237	1.36E-05	0.000182073
1450725_s_at	NM_011797	Car14	0.021875687	1.52E-05	0.000186513

1433964_s_at	BG066664	Mm.29670.2	0.021734274	1.75E-05	0.000190954
1450429_at	AI747133	Capn6	0.021631436	1.94E-05	0.000195395
1436178_at	AW553532	AW553532	0.021573815	2.05E-05	0.000199836
1421027_a_at	AI595932	Mef2c	0.021567213	2.06E-05	0.000204276
1453351_at	AK020409	9430010M06Rik	0.021370553	2.50E-05	0.000208717
1417466_at	NM_133736	1110070A02Rik	0.021332565	2.59E-05	0.000213158
1419593_at	NM_015764	5730583K22Rik	0.021275721	2.74E-05	0.000217599
1452001_at	L09600	Nfe2	0.021123965	3.17E-05	0.00022204
1416832_at	NM_026228	4933419D20Rik	0.021071726	3.33E-05	0.00022648
1424852_at	BB280300	Mef2c	0.020999986	3.57E-05	0.000230921
1448194_a_at	NM_023123	H19	0.020999421	3.57E-05	0.000235362
1447643_x_at	BB040443	Mm.111780.1	0.020953225	3.73E-05	0.000239803
1456014_s_at	BB113173	Mm.29670.3	0.020829603	4.19E-05	0.000244244
1434141_at	BG072799	Mm.32395.1	0.020665545	4.90E-05	0.000248684
1448554_s_at	NM_080728	Myh7	0.020618999	5.11E-05	0.000253125
1456180_at	AV333298	Mm.39720.1	0.02057246	5.34E-05	0.000257566
1449425_at	BC026373	Wnt2	0.020361322	6.50E-05	0.000262007
1418600_at	NM_010635	Klf1	0.020304184	6.85E-05	0.000266448
1434502_x_at	BB448377	Slc4a1	0.020269502	7.07E-05	0.000270888
1419829_a_at	AW049055	AI463667	0.020209632	7.47E-05	0.000275329
1416693_at	NM_013519	Foxc2	0.020182429	7.66E-05	0.00027977
1423222_at	AV261931	2810452G09Rik	0.020083377	8.39E-05	0.000284211
1450781_at	X58380	HMGI-C	0.020073856	8.46E-05	0.000288652
1455913_x_at	AV152953	Ttr	0.020036373	8.75E-05	0.000293092
1450428_at	AV335209	Lhx1	0.020026316	8.83E-05	0.000297533
1438020_at	BB036951	BB099155	0.019910981	9.81E-05	0.000301974
1437406_x_at	BB787243	Igfbp4	0.019809036	0.000107526	0.000306415
1418199_at	NM_053149	Hgn-pending	0.019794654	0.000108925	0.000310856
1449522_at	NM_009472	Unc5h3	0.019780051	0.000110364	0.000315296
1418084_at	AK011144	Nrp	0.019711592	0.000117349	0.000319737
1451046_at	AA014267	Zfpm1	0.019705693	0.00011797	0.000324178
1437347_at	BF100813	AU022549	0.01966762	0.000122054	0.000328619
1434465_x_at	AV333363	Vldlr	0.019490566	0.00014286	0.000333059
1454752_at	AV307961	AI606861	0.019333755	0.000164036	0.0003375
1440431_at	BB053468	Mm.210003.1	0.019290845	0.000170327	0.000341941
1417122_at	BC027242	Vav3	0.019280331	0.000171902	0.000346382
1451506_at	BB280300	Mef2c	0.019270694	0.000173358	0.000350823
1448553_at	NM_080728	Myh7	0.019211375	0.00018258	0.000355263
1423250_a_at	BF144658	Tgfb2	0.01919798	0.000184726	0.000359704
1437886_at	BM247104	Mm.86699.1	0.01916313	0.000190419	0.000364145
1449379_at	NM_010612	Kdr	0.019123532	0.000197088	0.000368586
1423756_s_at	BC019836	Igfbp4	0.018958722	0.000227277	0.000373027
1417184_s_at	BC027434	Hbb-b2	0.018856103	0.000248214	0.000377467
1450923_at	BF144658	Tgfb2	0.018823298	0.00025528	0.000381908
1450126_at	BB447551	Gata5	0.018777249	0.000265518	0.000386349

1423422_at	AV113827	Asb4	0.018762823	0.000268803	0.00039079
1455660_at	BB769628	AI848964	0.018739544	0.000274186	0.000395231
1418370_at	NM_009393	Tncc	0.018718975	0.000279025	0.000399671
1439946_at	BM221121	Mm.218554.1	0.018587209	0.000311981	0.000404112
1428444_at	AK003566	Asb2	0.018573369	0.000315646	0.000408553
1448471_a_at	NM_007796	Ctla2a	0.01848928	0.000338798	0.000412994
1438531_at	BM119567	Mm.111861.1	0.018417831	0.00035971	0.000417435
1455607_at	BG072958	AW742308	0.018414668	0.000360663	0.000421875
1447623_s_at	AV297026	Mm.141983.1	0.018334092	0.000385754	0.000426316
