# Supplementary material for: Estimating the Quality of Reprogrammed Cells Using ES Cell Differentiation Expression Patterns
Source: PLoS One. 2011 Jan 11;6(1):e15336. doi: 10.1371/journal.pone.0015336 (PMC3023460; doi:10.1371/journal.pone.0015336)
Supplement: Table S19 — Negative regulated genes in ES cell-derived Pancreatic islets cells Differentiation (GSE3653). (PDF) [file pone.0015336.s022.pdf]

Table S19 Negative regulated genes in ES cell-derived Pancreatic islets cells Differentiation (GSE3653)

Probe Set_ID	Acc_Num	Gene Name	Weight	P-value	FDR<0.1
1456909_at	BF017016	Mm.154410.1	-0.03675859	7.06E-14	4.44E-06
1417738_at	NM_016899	Rab25	-0.031993031	1.09E-10	8.88E-06
1422734_a_at	BC011513	Myb	-0.030791037	5.93E-10	1.33E-05
1450194_a_at	NM_033597	Myb	-0.030580965	7.92E-10	1.78E-05
1455966_s_at	BG070110	5730530J16Rik	-0.030164351	1.40E-09	2.22E-05
1440730_at	BB284266	Mm.132451.1	-0.028981267	6.69E-09	2.66E-05
1448136_at	BC003264	Enpp2	-0.028600477	1.09E-08	3.11E-05
1426186_a_at	AB016516	Fgf5	-0.028418947	1.38E-08	3.55E-05
1418911_s_at	NM_019477	Facl4	-0.028332295	1.54E-08	4.00E-05
1430735_at	AK015195	4930424G05Rik	-0.027822678	2.92E-08	4.44E-05
1419063_at	NM_011674	Ugt8	-0.027427225	4.76E-08	4.88E-05
1434700_at	BM123748	AW046379	-0.027395019	4.95E-08	5.33E-05
1453988_a_at	AK014703	Ide	-0.026659048	1.21E-07	5.77E-05
1421317_x_at	NM_033597	Myb	-0.026598948	1.30E-07	6.22E-05
1430177_at	AK011961	2610301N02Rik	-0.026418928	1.60E-07	6.66E-05
1448482_at	NM_026228	4933419D20Rik	-0.025781482	3.38E-07	7.11E-05
1417883_at	BC012707	Gstt2	-0.025532468	4.50E-07	7.55E-05
1437564_at	AV351762	Polg	-0.025290815	5.92E-07	7.99E-05
1421924_at	M75135	Slc2a3	-0.025285699	5.95E-07	8.44E-05
1459384_at	AV316207	Mm.212807.1	-0.025109337	7.26E-07	8.88E-05
1427770_a_at	X69698	Glut-3	-0.024985996	8.34E-07	9.33E-05
1442655_at	BB202790	Mm.122539.1	-0.024742212	1.09E-06	9.77E-05
1460132_at	BE944381	Mm.150378.1	-0.024727576	1.11E-06	0.000102138
1452424_at	AW493905	Mm.192098.1	-0.024618489	1.25E-06	0.000106579
1458687_at	BG070509	Mm.173259.1	-0.024464683	1.48E-06	0.00011102
1447181_s_at	AI790233	AI790233	-0.024302766	1.77E-06	0.000115461
1425362_at	BC003330	Mm.35097.1	-0.024152509	2.08E-06	0.000119901
1455896_a_at	AU043100	Kcnk1	-0.024088578	2.23E-06	0.000124342
1426165_a_at	D86352	Casp3	-0.024028484	2.38E-06	0.000128783
1421515_at	U09563	Nr6a1	-0.02392904	2.65E-06	0.000133224
1420342_at	NM_010268	Gdap10	-0.023630458	3.64E-06	0.000137665
1418351_a_at	NM_010068	Dnmt3b	-0.023452772	4.39E-06	0.000142105
1439305_at	BB011750	Mm.131842.1	-0.023376261	4.75E-06	0.000146546
1431701_a_at	AK006269	Pdzk1	-0.023229396	5.54E-06	0.000150987
1451701_x_at	BC012650	Cldn3	-0.023200407	5.71E-06	0.000155428
1440260_at	BG064532	Mm.102774.1	-0.023081264	6.45E-06	0.000159869
1437213_at	BG070110	5730530J16Rik	-0.022987513	7.11E-06	0.000164309
1453573_at	BB088582	Hist4	-0.022901065	7.77E-06	0.00016875
1449209_a_at	AB030503	Ube-1a	-0.022746941	9.09E-06	0.000173191
1445681_at	BE852160	Mm.163189.1	-0.022741567	9.14E-06	0.000177632

1448566_at	AF226613	Fpn1	-0.022713079	9.41E-06	0.000182073
1443339_at	AV373786	Mm.212991.1	-0.022654751	9.98E-06	0.000186513
1451348_at	BC004774	Mm.152666.1	-0.022550199	1.11E-05	0.000190954
1427912_at	AK003232	1110001J05Rik	-0.022402627	1.29E-05	0.000195395
1457692_at	BB641320	Mm.215696.1	-0.022341361	1.37E-05	0.000199836
1443353_at	BB201882	Mm.207472.1	-0.02221288	1.55E-05	0.000204276
1424809_at	BC024462	Mm.23982.1	-0.021966732	1.98E-05	0.000208717
1459078_at	BG069377	Mm.221062.1	-0.021917606	2.08E-05	0.000213158
1455840_at	BM230524	C86120	-0.021904038	2.11E-05	0.000217599
1434357_a_at	AW544889	Impnb	-0.021846544	2.23E-05	0.00022204
1444599_at	BB053466	Mm.183744.1	-0.0218423	2.24E-05	0.00022648
1419157_at	AI428101	Sox4	-0.021837363	2.25E-05	0.000230921
1422072_a_at	NM_008184	Gstm6	-0.021834838	2.25E-05	0.000235362
1429900_at	BM241296	5330406M23Rik	-0.021778727	2.38E-05	0.000239803
1448949_at	NM_007607	Car4	-0.021712055	2.54E-05	0.000244244
1441967_at	BB467791	Mm.139810.1	-0.021598691	2.83E-05	0.000248684
1442618_at	BB477709	Mm.134720.1	-0.021511295	3.08E-05	0.000253125
1422962_a_at	NM_010724	Psmb8	-0.021374341	3.52E-05	0.000257566
1445517_at	BB144876	Mm.212804.1	-0.021278298	3.85E-05	0.000262007
1428642_at	AK018094	6230421J19Rik	-0.021242124	3.99E-05	0.000266448
1444318_at	BF020847	Mm.86694.1	-0.021191277	4.18E-05	0.000270888
1431233_at	AK017367	5430430O18Rik	-0.021098087	4.57E-05	0.000275329
1422967_a_at	BB810450	Trfr	-0.021078752	4.65E-05	0.00027977
1443509_at	AU042807	Mm.214915.1	-0.020921052	5.39E-05	0.000284211
1446204_at	BB142014	Mm.215429.1	-0.020918429	5.41E-05	0.000288652
1418688_at	NM_007588	Calcr	-0.020694073	6.66E-05	0.000293092
1416776_at	NM_016669	Crym	-0.020681698	6.74E-05	0.000297533
1422882_at	BE333485	Pphn	-0.020671788	6.80E-05	0.000301974
1425245_a_at	BC019741	Rgs11	-0.020592581	7.31E-05	0.000306415
1440594_at	BG069643	Mm.103999.1	-0.020587203	7.35E-05	0.000310856
1442622_at	BB165231	Mm.218768.1	-0.020583304	7.37E-05	0.000315296
1417588_at	AK019995	Galnt3	-0.020572503	7.45E-05	0.000319737
1451777_at	BC013672	Mm.33332.1	-0.020542441	7.66E-05	0.000324178
1457491_at	BM238642	Mm.215460.1	-0.020345051	9.17E-05	0.000328619
1450947_at	AK012175	2610528J11Rik	-0.020206708	0.000103904	0.000333059
1428680_at	AK014670	4833409J18Rik	-0.020102926	0.00011407	0.0003375
1436317_at	BM115569	Mm.103539.1	-0.020064415	0.000118076	0.000341941
1435169_at	BB431047	Mm.52252.2	-0.020060605	0.000118479	0.000346382
1417895_a_at	BC019563	1810017F10Rik	-0.020001139	0.000124947	0.000350823
1448607_at	AW989410	Pbef-pending	-0.019971289	0.000128318	0.000355263
1418094_s_at	NM_007607	Car4	-0.019915297	0.000134873	0.000359704
1450738_at	NM_016705	Kif21a	-0.019893853	0.000137467	0.000364145
1442467_at	AU067780	AU067780	-0.019891908	0.000137704	0.000368586

1429680_at	BF466228	1500010G04Rik	-0.019820902	0.000146645	0.000373027
1448538_a_at	BE447520	D4Wsu53e	-0.019730476	0.000158825	0.000377467
1459185_at	BG072840	Mm.197239.1	-0.019573094	0.000182324	0.000381908
1425767_a_at	D50416	Six4	-0.019532616	0.000188876	0.000386349
1416702_at	NM_009250	Serpini1	-0.019511324	0.000192411	0.00039079
1449325_at	NM_019699	Fads2	-0.019503164	0.000193783	0.000395231
1453824_at	BG085812	A930028L21Rik	-0.019497173	0.000194795	0.000399671
1437540_at	AV313762	Mcoln3	-0.019480923	0.000197567	0.000404112
1439477_at	BB047737	Mm.25573.1	-0.019470307	0.000199397	0.000408553
1450378_at	AF043943	Tapbp	-0.019434356	0.000205716	0.000412994
1426332_a_at	AF087821	Cldn3	-0.019379222	0.000215772	0.000417435
1452297_at	BI872151	Mm.45584.1	-0.019373464	0.000216848	0.000421875
1449862_a_at	NM_028744	Pi4k2b-pending	-0.019247876	0.000241621	0.000426316
1431322_at	AK019524	4833439O17Rik	-0.019242154	0.000242811	0.000430757
1454215_at	AK010436	2410007B07Rik	-0.019218483	0.000247791	0.000435198
1439850_at	BB310805	Mm.131965.1	-0.019205106	0.000250648	0.000439639
1442886_at	BB667153	Mm.107289.1	-0.019173336	0.000257556	0.000444079
1447272_s_at	BM249532	Mm.217468.1	-0.019096336	0.000275049	0.00044852
1452638_s_at	BC027538	Mm.221051.1	-0.019086809	0.00027729	0.000452961
1458668_at	AI427778	Mm.150611.1	-0.019069072	0.000281506	0.000457402
1442681_at	AV294722	Mm.212975.1	-0.019048379	0.000286501	0.000461842
1431169_at	BG803764	5033403E17Rik	-0.019031603	0.000290611	0.000466283
1437219_at	AW553541	Mm.87267.1	-0.018943316	0.000313169	0.000470724
1416147_at	BE912771	Hspa4	-0.018906454	0.000323064	0.000475165
1455115_a_at	AU015319	Mm.23982.2	-0.01885363	0.000337756	0.000479606
1446085_at	BB022048	Mm.207132.1	-0.01884102	0.000341354	0.000484046
1448690_at	NM_008430	Kcnk1	-0.018817191	0.000348252	0.000488487
1448364_at	U95826	Ccng2	-0.018753719	0.000367268	0.000492928
1459497_at	AU019852	Mm.23234.1	-0.018710105	0.000380893	0.000497369
1456533_at	BM119324	Mm.191939.1	-0.018695625	0.000385521	0.00050181
1428946_at	BB417360	5730469D23Rik	-0.018569614	0.000428065	0.00050625
1421881_a_at	BB105998	Elavl2	-0.018552647	0.000434117	0.000510691
1460063_at	BB230617	D5Ertd798e	-0.018548878	0.000435473	0.000515132
1429262_at	AK005472	1600016B17Rik	-0.018546813	0.000436217	0.000519573
1451486_at	BC006902	1200006F02Rik	-0.018525866	0.000443834	0.000524014
1417216_at	NM_138606	DXCch3	-0.018511555	0.000449108	0.000528454
1442046_at	BM119167	Mm.215733.1	-0.018502457	0.000452492	0.000532895
1445746_at	BB118894	Mm.208089.1	-0.01848946	0.000457368	0.000537336
1427735_a_at	M12233	Acta1	-0.018456264	0.000470043	0.000541777
1416157_at	NM_009502	Vcl	-0.01844557	0.000474196	0.000546218
1416132_at	BB188557	C920006C10Rik	-0.018435824	0.00047801	0.000550658
1441317_x_at	BB316060	Mm.131980.1	-0.018416462	0.000485673	0.000555099
1420583_a_at	NM_013646	Rora	-0.01839682	0.000493564	0.00055954

1441677_at	BM244144	Mm.157408.1	-0.018390106	0.000496289	0.000563981
1443256_at	BB548833	Mm.137360.1	-0.018386316	0.000497833	0.000568422
1422643_at	NM_021509	3230402N08Rik	-0.018347528	0.000513896	0.000572862
1416188_at	BC004651	Gm2a	-0.018288236	0.000539388	0.000577303
1438641_x_at	BB391156	Mm.62181.1	-0.018278928	0.000543495	0.000581744
1432344_a_at	AK013376	Aplp2	-0.018240978	0.000560544	0.000586185
