# Supplementary material for: Estimating the Quality of Reprogrammed Cells Using ES Cell Differentiation Expression Patterns
Source: PLoS One. 2011 Jan 11;6(1):e15336. doi: 10.1371/journal.pone.0015336 (PMC3023460; doi:10.1371/journal.pone.0015336)
Supplement: Table S20 — Positive regulated genes in ES cell-derived Pancreatic islets cells Differentiation (GSE3653). (PDF) [file pone.0015336.s023.pdf]

Table S20 Positive regulated genes in ES cell-derived Pancreatic islets cells Differentiation (GSE3653)

Probe Set_ID	Acc_Num	Gene Name	Weight	P-value	FDR<0.1
1427883_a_at	AW550625	Col3a1	0.058471842	1.40E-34	4.44E-06
1450857_a_at	BF227507	Col1a2	0.056477587	2.53E-32	8.88E-06
1423606_at	BI110565	Osf2-pending	0.052162295	1.04E-27	1.33E-05
1423110_at	BF227507	Col1a2	0.047824188	1.95E-23	1.78E-05
1416168_at	NM_011340	Serpinf1	0.046145725	6.97E-22	2.22E-05
1437990_x_at	AV147727	Hbb-bh1	0.045958738	1.03E-21	2.66E-05
1416405_at	BC019502	Bgn	0.045469405	2.84E-21	3.11E-05
1437810_a_at	AV311770	Hbb-bh1	0.045441513	3.01E-21	3.55E-05
1416342_at	NM_011607	Tnc	0.044816709	1.08E-20	4.00E-05
1450757_at	NM_009866	Cdh11	0.04399251	5.70E-20	4.44E-05
1436823_x_at	AV148191	Hbb-y	0.039669134	2.10E-16	4.88E-05
1434376_at	AW146109	AW146109	0.038989806	7.05E-16	5.33E-05
1454677_at	BF168458	D11Bwg1104e	0.038824167	9.45E-16	5.77E-05
1416529_at	U25633	TMP	0.038799922	9.86E-16	6.22E-05
1423669_at	U08020	Col1a1	0.038294686	2.39E-15	6.66E-05
1436717_x_at	AV156860	Hbb-y	0.038219328	2.72E-15	7.11E-05
1448943_at	AK011144	Nrp	0.037750068	6.12E-15	7.55E-05
1448326_a_at	NM_013496	Crabp1	0.036645619	3.95E-14	7.99E-05
1420337_at	L39770	Gbx-2	0.036402714	5.92E-14	8.44E-05
1427760_s_at	X75557	mrpplf	0.035631538	2.09E-13	8.88E-05
1448416_at	NM_008597	Mglap	0.035276222	3.70E-13	9.33E-05
1421917_at	AW537708	Pdgfra	0.035163571	4.44E-13	9.77E-05
1448323_a_at	BC019502	Bgn	0.035059871	5.23E-13	0.000102138
1438658_a_at	AV238324	AI132464	0.035003618	5.72E-13	0.000106579
1450736_a_at	NM_008219	Hbb-bh1	0.034583639	1.11E-12	0.00011102
1434745_at	BQ175880	AA087124	0.034548273	1.18E-12	0.000115461
1449939_s_at	NM_010052	Dlk1	0.034505146	1.26E-12	0.000119901
1417133_at	NM_008885	Pmp22	0.034345679	1.61E-12	0.000124342
1424131_at	AF064749	Col6a3	0.034294162	1.75E-12	0.000128783
1448194_a_at	NM_023123	H19	0.034082159	2.43E-12	0.000133224
1428361_x_at	AK011116	Hba-a1	0.034025439	2.66E-12	0.000137665
1448613_at	NM_007899	Ecm1	0.033887064	3.29E-12	0.000142105
1418726_a_at	NM_011619	Tnnt2	0.033193009	9.47E-12	0.000146546
1448716_at	M26898	Hba-x	0.032846129	1.59E-11	0.000150987
1448590_at	NM_009933	Col6a1	0.032773841	1.78E-11	0.000155428
1457632_s_at	BB207647	Mm.215008.1	0.032532454	2.54E-11	0.000159869
1454974_at	BI143915	AI561871	0.032443237	2.90E-11	0.000164309
1424542_at	D00208	S100a4	0.032407958	3.05E-11	0.00016875
1426864_a_at	BB698413	Ncam1	0.032390505	3.13E-11	0.000173191
1426208_x_at	AF147785	Zac1	0.032193302	4.19E-11	0.000177632
1448123_s_at	NM_009369	Tgfbi	0.032115531	4.69E-11	0.000182073
1423760_at	M27130	Cd44	0.031933749	6.11E-11	0.000186513

1452757_s_at	AK011116	Hba-a1	0.031716685	8.37E-11	0.000190954
1452217_at	BE570050	AA589382	0.031637165	9.38E-11	0.000195395
1449145_a_at	AB029929	Cav	0.03161199	9.73E-11	0.000199836
1418153_at	NM_008480	Lama1	0.031587139	1.01E-10	0.000204276
1422914_at	NM_022435	Sp5	0.03143988	1.24E-10	0.000208717
1456110_at	BB320513	Mm.41929.2	0.031356951	1.40E-10	0.000213158
1425528_at	L06502	Prrx1	0.031198865	1.75E-10	0.000217599
1416740_at	AW744319	Col5a1	0.031158061	1.86E-10	0.00022204
1447643_x_at	BB040443	Mm.111780.1	0.031033675	2.22E-10	0.00022648
1422437_at	AV229424	Col5a2	0.03084138	2.91E-10	0.000230921
1437165_a_at	BB250811	Pcolce	0.030805145	3.06E-10	0.000235362
1452114_s_at	BF225802	Igfbp5	0.03078922	3.13E-10	0.000239803
1418467_at	NM_025891	Smarcd3	0.030694817	3.57E-10	0.000244244
1455899_x_at	BB241535	Cish3	0.030625078	3.93E-10	0.000248684
1418084_at	AK011144	Nrp	0.030590165	4.13E-10	0.000253125
1434479_at	AV246911	AI413331	0.030508614	4.62E-10	0.000257566
1423584_at	AI481026	Igfbp7	0.030317121	6.02E-10	0.000262007
1437463_x_at	BB532080	Tgfbi	0.030223731	6.85E-10	0.000266448
1421811_at	AI385532	Thbs1	0.030058879	8.59E-10	0.000270888
1448471_a_at	NM_007796	Ctla2a	0.029943188	1.01E-09	0.000275329
1455494_at	BI794771	Col1a1	0.029723635	1.35E-09	0.00027977
1456292_a_at	AV147875	Vim	0.029551743	1.71E-09	0.000284211
1450641_at	M24849	Vim	0.029443325	1.98E-09	0.000288652
1448254_at	BC002064	Ptn	0.029434651	2.00E-09	0.000293092
1436869_at	AV304616	Mm.129748.1	0.029351593	2.23E-09	0.000297533
1423407_a_at	BF228318	Fbln2	0.029108694	3.09E-09	0.000301974
1415938_at	NM_009258	Spink3	0.028741575	5.00E-09	0.000306415
1417065_at	NM_007913	Egr1	0.028711572	5.20E-09	0.000310856
1422603_at	BC005569	Rnase4	0.028615025	5.90E-09	0.000315296
1424967_x_at	L47552	Tnnt2	0.028598991	6.03E-09	0.000319737
1426865_a_at	BB698413	Ncam1	0.028541157	6.50E-09	0.000324178
1456212_x_at	BB831725	Cish3	0.028194923	1.02E-08	0.000328619
1440739_at	AW228853	AW228853	0.028189801	1.02E-08	0.000333059
1436041_at	BB029192	AI661148	0.028188882	1.02E-08	0.0003375
1421375_a_at	NM_011313	S100a6	0.028156577	1.07E-08	0.000341941
1426858_at	BB253137	Inhbb	0.028134573	1.10E-08	0.000346382
1448152_at	NM_010514	Igf2	0.028119115	1.12E-08	0.000350823
1416122_at	NM_009829	Ccnd2	0.02797934	1.34E-08	0.000355263
1455090_at	BF681826	AW260363	0.027973661	1.35E-08	0.000359704
1455956_x_at	AV310588	Ccnd2	0.027957252	1.38E-08	0.000364145
1438118_x_at	AV147875	Vim	0.027872717	1.53E-08	0.000368586
1415806_at	NM_008872	Plat	0.027770387	1.74E-08	0.000373027
1437171_x_at	AV025667	Gsn	0.027494607	2.47E-08	0.000377467
1421840_at	BB144704	Abca1	0.027379687	2.85E-08	0.000381908
1418157_at	NM_010151	Nr2f1	0.027310118	3.11E-08	0.000386349

1415931_at	NM_010514	Igf2	0.027256999	3.32E-08	0.00039079
1448213_at	NM_010730	Anxa1	0.027250768	3.34E-08	0.000395231
1436026_at	BI558298	AI430822	0.027195486	3.58E-08	0.000399671
1456250_x_at	BB533460	Tgfbi	0.027002424	4.54E-08	0.000404112
1416749_at	NM_019564	Prss11	0.026959177	4.79E-08	0.000408553
1423607_at	AK014312	Lum	0.02695137	4.84E-08	0.000412994
1436991_x_at	AV025559	Gsn	0.02687011	5.35E-08	0.000417435
1418133_at	NM_033601	Bcl3	0.026806762	5.78E-08	0.000421875
1436938_at	BB053506	Mm.133242.1	0.026782187	5.95E-08	0.000426316
1455096_at	AW555664	Mm.100844.1	0.026437255	9.04E-08	0.000430757
1423835_at	BB447914	Mm.21082.1	0.026412965	9.31E-08	0.000435198
1433919_at	AV302111	Asb4	0.026192596	1.21E-07	0.000439639
1438251_x_at	BB559067	Prss11	0.026187253	1.22E-07	0.000444079
1433776_at	AV149705	AI194968	0.026107547	1.34E-07	0.00044852
1436970_a_at	AA499047	Pdgfrb	0.026049653	1.44E-07	0.000452961
1448433_a_at	NM_008788	Pcolce	0.026012836	1.50E-07	0.000457402
1417129_a_at	U68384	Mrg1b	0.025949848	1.62E-07	0.000461842
1439766_x_at	BB089170	Mm.58184.1	0.025946892	1.62E-07	0.000466283
1418379_s_at	NM_054044	9530074E10Rik	0.025869528	1.78E-07	0.000470724
1427884_at	AW550625	Col3a1	0.025546708	2.59E-07	0.000475165
1416576_at	NM_007707	Cish3	0.025483593	2.79E-07	0.000479606
1455160_at	BM220421	AI848448	0.025444604	2.92E-07	0.000484046
1448229_s_at	NM_009829	Ccnd2	0.025416779	3.02E-07	0.000488487
1416158_at	AI463873	Nr2f2	0.025354695	3.24E-07	0.000492928
1460220_a_at	BM233698	Csf1	0.025231597	3.74E-07	0.000497369
1419417_at	NM_009506	Vegfc	0.025217128	3.80E-07	0.00050181
1451978_at	AF357006	Loxl1	0.025160981	4.05E-07	0.00050625
1457670_s_at	AV238225	Mm.87119.1	0.025147771	4.11E-07	0.000510691
1460302_at	AI385532	Thbs1	0.025034803	4.68E-07	0.000515132
1448826_at	BB481540	Myhca	0.025027453	4.72E-07	0.000519573
1423477_at	BB361162	Zic1	0.024982129	4.97E-07	0.000524014
1438651_a_at	BB483357	Agtrl1	0.024978147	4.99E-07	0.000528454
1419149_at	NM_008871	Serpine1	0.024966078	5.06E-07	0.000532895
1438403_s_at	BF537798	Ramp2	0.024911875	5.38E-07	0.000537336
1427233_at	AV291373	Sdccag33	0.024889747	5.52E-07	0.000541777
1426397_at	BG793483	1110020H15Rik	0.024873988	5.62E-07	0.000546218
1424186_at	BG074158	urb	0.024793601	6.15E-07	0.000550658
1416811_s_at	NM_007796	Ctla2a	0.024639685	7.32E-07	0.000555099
1450958_at	BQ177170	Tm4sf1	0.024539554	8.19E-07	0.00055954
1422580_at	NM_010858	Myla	0.024501179	8.55E-07	0.000563981
1416645_a_at	NM_007423	Afp	0.024457011	8.98E-07	0.000568422
1450429_at	AI747133	Capn6	0.024388932	9.68E-07	0.000572862
1460287_at	M93954	TIMP2	0.02435973	1.00E-06	0.000577303
1417395_at	BG069413	Klf4	0.024321395	1.04E-06	0.000581744
1423836_at	BB447914	Mm.21082.1	0.024228482	1.16E-06	0.000586185

1434895_s_at	BG064715	AI449786	0.024172205	1.23E-06	0.000590625
1435456_at	AW552254	AI428795	0.02415928	1.25E-06	0.000595066
1450781_at	X58380	HMGI-C	0.02408335	1.36E-06	0.000599507
1424086_at	BC025514	Mm.3776.1	0.024015569	1.46E-06	0.000603948
1449071_at	NM_022879	Mylc2a	0.023939296	1.59E-06	0.000608389
1437479_x_at	BB728182	BB131012	0.023761877	1.93E-06	0.000612829
1450992_a_at	AW547821	Meis1	0.023715696	2.03E-06	0.00061727
1423586_at	AA500897	Axl	0.02366397	2.14E-06	0.000621711
1431057_a_at	AK009847	2310046G15Rik	0.023590763	2.32E-06	0.000626152
1460208_at	NM_007993	Fbn1	0.023487375	2.59E-06	0.000630593
1430127_a_at	AK007904	Ccnd2	0.023482473	2.60E-06	0.000635033
1451382_at	BC025169	1810008K03Rik	0.02330931	3.13E-06	0.000639474
1436791_at	BB067079	Wnt5a	0.023249139	3.34E-06	0.000643915
1418517_at	NM_008393	Irx3	0.023241386	3.37E-06	0.000648356
1424051_at	BC013560	Col4a2	0.02320943	3.48E-06	0.000652797
1448944_at	AK011144	Nrp	0.023209151	3.48E-06	0.000657237
1452035_at	BF158638	Col4a1	0.023062768	4.06E-06	0.000661678
1418926_at	NM_011546	Zfhx1a	0.022782086	5.45E-06	0.000666119
1450813_a_at	NM_021467	Tnni1	0.022769552	5.52E-06	0.00067056
1450922_a_at	BF144658	Tgfb2	0.022766017	5.54E-06	0.000675001
1455913_x_at	AV152953	Ttr	0.022713546	5.85E-06	0.000679441
1425220_x_at	AF067062	variable group of 2-cell-stage gene family	0.022691481	5.99E-06	0.000683882
1422831_at	NM_010181	Fbn2	0.022641046	6.31E-06	0.000688323
1438702_at	BG075699	Mm.103204.1	0.022618007	6.46E-06	0.000692764
1436568_at	AU016127	AU016127	0.022555285	6.89E-06	0.000697205
1423250_a_at	BF144658	Tgfb2	0.022552334	6.91E-06	0.000701645
1418538_at	NM_134090	AI173274	0.022548648	6.94E-06	0.000706086
1416614_at	BC010712	ORF12	0.022319848	8.77E-06	0.000710527
1418733_at	NM_011658	Twist	0.022266011	9.26E-06	0.000714968
1456733_x_at	BB329489	Serpinh1	0.022160631	1.03E-05	0.000719408
1434530_at	BQ175876	R75022	0.022140609	1.05E-05	0.000723849
1417930_at	NM_008668	Nab2	0.022112865	1.08E-05	0.00072829
1418876_at	BB662927	Foxd1	0.022108164	1.09E-05	0.000732731
1416414_at	NM_133918	AW229038	0.022050409	1.15E-05	0.000737172
1459737_s_at	AA408768	AA408768	0.02198913	1.22E-05	0.000741612
1421654_a_at	NM_019390	Lmna	0.021915875	1.32E-05	0.000746053
1422912_at	NM_007554	Bmp4	0.021889726	1.35E-05	0.000750494
1416808_at	X14480	Nid1	0.02186865	1.38E-05	0.000754935
1452250_a_at	BI455189	Col6a2	0.021812826	1.46E-05	0.000759376
1436546_at	BM502719	Mm.88628.1	0.02177053	1.52E-05	0.000763816
1457033_at	BM198753	Mm.131747.1	0.021696275	1.64E-05	0.000768257
1419486_at	BB759833	Foxc1	0.02168061	1.67E-05	0.000772698
1423422_at	AV113827	Asb4	0.021635873	1.74E-05	0.000777139
1416646_at	NM_007423	Afp	0.021634423	1.74E-05	0.00078158

1448553_at	NM_080728	Myh7	0.021587703	1.83E-05	0.00078602
1426454_at	AK002516	Arhgdib	0.021582533	1.83E-05	0.000790461
1416121_at	M65143	Lox	0.021563209	1.87E-05	0.000794902
1448593_at	NM_018865	Wisp1	0.021550074	1.89E-05	0.000799343
1426238_at	L24755	Bmp-1	0.021547538	1.90E-05	0.000803784
1427768_s_at	X67685	Mylc	0.021532745	1.93E-05	0.000808224
1450923_at	BF144658	Tgfb2	0.021512495	1.97E-05	0.000812665
1417104_at	BC001999	Emp3	0.021478953	2.03E-05	0.000817106
1431088_at	BQ286886	1200010K03Rik	0.021473764	2.04E-05	0.000821547
1454674_at	AU067669	Mm.5264.1	0.021392363	2.21E-05	0.000825988
1453102_at	BE945486	5530600M07Rik	0.021302692	2.41E-05	0.000830428
1423812_s_at	BC024822	Mm.24642.1	0.021245424	2.55E-05	0.000834869
1437442_at	BG067986	Mm.25535.1	0.021233837	2.58E-05	0.00083931
1416123_at	NM_009829	Ccnd2	0.021216418	2.62E-05	0.000843751
1437671_x_at	BB378796	2310046G15Rik	0.021196577	2.67E-05	0.000848191
1417649_at	NM_009876	Cdkn1c	0.02113808	2.83E-05	0.000852632
1450621_a_at	NM_008221	Hbb-y	0.021112278	2.90E-05	0.000857073
1434369_a_at	AV016515	Cryab	0.020980021	3.29E-05	0.000861514
1450723_at	BQ176915	Isl1	0.020933031	3.44E-05	0.000865955
1428571_at	AK004383	Col9a1	0.020923583	3.48E-05	0.000870395
1449520_at	AW492543	BC002262	0.020902705	3.55E-05	0.000874836
1419584_at	AW492543	BC002262	0.020804198	3.89E-05	0.000879277
1419304_at	NM_009309	T	0.020761099	4.06E-05	0.000883718
1454849_x_at	BB433678	Clu	0.020756296	4.08E-05	0.000888159
1449070_x_at	BB770932	EIG180	0.020714049	4.24E-05	0.000892599
1443961_at	BG071029	AU017962	0.020656521	4.48E-05	0.00089704
1433924_at	BM200248	Peg3	0.020636451	4.56E-05	0.000901481
1415812_at	NM_010354	Gsn	0.020395044	5.72E-05	0.000905922
1417439_at	NM_054042	Tem1-pending	0.020392436	5.74E-05	0.000910363
1416503_at	NM_016753	Lxn	0.020328438	6.09E-05	0.000914803
1417447_at	NM_011545	Tcf21	0.020326079	6.10E-05	0.000919244
1448797_at	BC005686	Elk3	0.020214254	6.77E-05	0.000923685
1454666_at	AV230488	Mm.30693.1	0.020198909	6.86E-05	0.000928126
1456778_at	BB409477	Mm.186992.1	0.020055425	7.83E-05	0.000932567
1424638_at	AK007630	Cdkn1a	0.020054106	7.84E-05	0.000937007
1453055_at	BB462688	1110067B02Rik	0.020009261	8.17E-05	0.000941448
1426947_x_at	BI455189	Col6a2	0.020001146	8.23E-05	0.000945889
1418188_a_at	AF146523	Ramp2	0.019927517	8.80E-05	0.00095033
1437889_x_at	AI931862	Bgn	0.019913525	8.91E-05	0.000954771
1450780_s_at	X58380	Hmga2	0.019880654	9.18E-05	0.000959211
1449590_a_at	AB004879	Mras	0.019864448	9.32E-05	0.000963652
1418136_at	NM_009365	Tgfb1i1	0.019811035	9.78E-05	0.000968093
1455280_at	BI452538	Mm.116505.1	0.019742028	0.000104096	0.000972534
1417937_at	NM_021532	Thyex3-pending	0.019731351	0.000105102	0.000976974
1416211_a_at	BC002064	Ptn	0.019707209	0.000107413	0.000981415

1450350_a_at	NM_030887	Jundp2-pending	0.01966349	0.000111719	0.000985856
1439498_at	BG093601	AI851425	0.019621912	0.000115965	0.000990297
1416159_at	AI463873	Nr2f2	0.019588102	0.00011953	0.000994738
1460330_at	AW702161	Anxa3	0.01956759	0.000121743	0.000999178
1426225_at	U63146	RBP	0.019529	0.000126011	0.001003619
1440935_at	BQ176837	Mm.212560.1	0.019499021	0.000129424	0.00100806
1425896_a_at	AF007248	Fbn1	0.019456344	0.000134433	0.001012501
1432331_a_at	AK019971	Prrx2	0.019381456	0.000143667	0.001016942
1420957_at	NM_007462	Apc	0.019379784	0.00014388	0.001021382
1448818_at	BC018425	Wnt5a	0.019366702	0.000145556	0.001025823
1418317_at	NM_010710	Lhx2	0.019360358	0.000146375	0.001030264
1448995_at	NM_019932	Pf4	0.019355247	0.000147038	0.001034705
1454991_at	BB264620	AI447493	0.019284748	0.000156478	0.001039146
1425427_at	AF067058	variable group of 2-cell-stage gene family	0.019273901	0.00015798	0.001043586
1430700_a_at	AK005158	Pla2g7	0.019243894	0.000162207	0.001048027
1421072_at	NM_018826	Irx5	0.019145332	0.000176851	0.001052468
1450644_at	M58566	Zfp36l1	0.019139967	0.000177683	0.001056909
1429719_at	AK009204	2310007G05Rik	0.019113588	0.000181827	0.00106135
1424886_at	BC025145	Ptprd	0.019111576	0.000182147	0.00106579
1454822_x_at	BB271021	EIG180	0.019109095	0.000182542	0.001070231
1425476_at	BM250666	Col4a5	0.019092243	0.000185246	0.001074672
1416454_s_at	NM_007392	Acta2	0.019052348	0.0001918	0.001079113
1427445_a_at	BC025840	1100001C23Rik	0.019049155	0.000192334	0.001083554
1417466_at	NM_133736	1110070A02Rik	0.019029912	0.000195581	0.001087994
1423628_s_at	BB731207	Pcdhgc5	0.018990017	0.000202477	0.001092435
1437689_x_at	AV152288	Clu	0.018956578	0.000208433	0.001096876
1427769_x_at	X67685	Mylc	0.018912629	0.000216512	0.001101317
1442025_a_at	AI467657	AI467657	0.01888789	0.000221188	0.001105757
1450377_at	AI385532	Thbs1	0.018887826	0.0002212	0.001110198
1437633_at	BB320513	Mm.41929.2	0.018856854	0.000227188	0.001114639
1428909_at	C85657	1200015M12Rik	0.018759039	0.000247115	0.00111908
1422053_at	NM_008380	Inhba	0.018742624	0.000250615	0.001123521
1448925_at	NM_007855	Dermo1	0.018740834	0.000251	0.001127961
1423071_x_at	AW549928	6720475J19Rik	0.018725126	0.000254398	0.001132402
1416625_at	NM_009776	Serping1	0.018703383	0.000259174	0.001136843
1424659_at	BG963150	Slit2	0.018691559	0.000261806	0.001141284
1454608_x_at	BG141874	Ttr	0.018667185	0.00026731	0.001145725
1453724_a_at	AK012411	Serpinf1	0.018663831	0.000268076	0.001150165
1419065_at	NM_025684	5730521E12Rik	0.018636532	0.000274388	0.001154606
1451046_at	AA014267	Zfpm1	0.018566296	0.000291272	0.001159047
1417394_at	BG069413	Klf4	0.018564199	0.00029179	0.001163488
1448405_a_at	BC010712	ORF12	0.018552611	0.000294673	0.001167929
1420360_at	NM_010051	Dkk1	0.018528081	0.000300862	0.001172369
1448601_s_at	BC016426	Msx1	0.018508784	0.000305817	0.00117681

1416803_at	NM_010222	Fkbp7	0.018472401	0.000315367	0.001181251
1454966_at	BQ175493	AI447669	0.018452491	0.000320711	0.001185692
1422851_at	X58380	Hmga2	0.018435927	0.000325221	0.001190133
1448554_s_at	NM_080728	Myh7	0.018424257	0.000328434	0.001194573
1452183_a_at	Y13832	Meg3	0.018411117	0.000332088	0.001199014
1416257_at	NM_009794	Capn2	0.018390166	0.000337992	0.001203455
1430781_at	AV256298	4930502N02Rik	0.018377003	0.000341752	0.001207896
1434378_a_at	BG868949	Lamb1-1	0.01834443	0.000351224	0.001212337
1426246_at	Z25469	Pros1	0.018279996	0.000370689	0.001216777
1431375_s_at	BI690209	2010012A22Rik	0.018259989	0.000376936	0.001221218
1449351_s_at	NM_019971	Pdgfc	0.01825672	0.000377966	0.001225659
1449397_at	NM_134032	AI894218	0.018235259	0.000384793	0.0012301
1456139_at	BM124989	Mm.138484.1	0.01817426	0.000404833	0.00123454
1433662_s_at	BF168458	D11Bwg1104e	0.018083903	0.000436317	0.001238981
1426413_at	BM116592	Neurod1	0.018035276	0.000454193	0.001243422
1447861_x_at	AV329643	Mm.92045.1	0.018003975	0.000466059	0.001247863
1450397_at	BB731480	Mtap1b	0.017971772	0.000478568	0.001252304
1423824_at	BC018381	5031439A09Rik	0.017919291	0.000499628	0.001256744
1453009_at	AK004327	1110060I01Rik	0.01789655	0.00050902	0.001261185
1434286_at	BQ176054	AI115454	0.017894346	0.000509939	0.001265626
1435777_at	BB794845	Mm.1759.1	0.017884766	0.000513951	0.001270067
1441107_at	BB292639	Mm.32825.1	0.017843705	0.000531483	0.001274508
1437458_x_at	AV075715	Clu	0.017830264	0.000537342	0.001278948
1436931_at	AV255458	Mm.32654.1	0.017819043	0.000542279	0.001283389
1419095_a_at	NM_018816	Apom	0.017815679	0.000543768	0.00128783
1436742_a_at	BG070524	Mm.9889.1	0.017775258	0.000561953	0.001292271
1434285_at	BB701578	2700017I06Rik	0.017744296	0.000576264	0.001296712
1418496_at	NM_008259	Foxa1	0.017742742	0.000576991	0.001301152
1417862_at	NM_021427	AB041569	0.017735259	0.000580504	0.001305593
1425603_at	BC006049	Mm.153276.1	0.0177118	0.000591648	0.001310034
1450716_at	D67076	ADAMTS-1	0.017709121	0.000592933	0.001314475
1451416_a_at	BC026422	Tgm1	0.017678709	0.000607707	0.001318916
1437434_a_at	BM241735	5031439A09Rik	0.017678114	0.000607999	0.001323356
1427005_at	BM234765	Snk	0.017676473	0.000608806	0.001327797
1455101_at	BE631955	AV158170	0.017632171	0.000630978	0.001332238
1433481_at	BB027759	Mm.64032.1	0.017615565	0.000639481	0.001336679
1452903_at	AK020090	6230427J02Rik	0.017582869	0.000656535	0.00134112
1415871_at	NM_009369	Tgfbi	0.017582855	0.000656542	0.00134556
1440091_at	BB212184	Mm.209554.1	0.01757034	0.000663181	0.001350001
1421679_a_at	NM_007669	Cdkn1a	0.017569346	0.000663711	0.001354442
1418370_at	NM_009393	Tncc	0.017566808	0.000665066	0.001358883
1456312_x_at	AV224521	Gsn	0.017546935	0.000675765	0.001363323
1435197_at	BE993443	Mm.40572.1	0.017543085	0.000677856	0.001367764
1418382_at	BB770932	EIG180	0.017541541	0.000678696	0.001372205
1417143_at	U70622	Edg2	0.017538	0.000680627	0.001376646

1455151_at	C79026	C79026	0.017515031	0.000693276	0.001381087
1451413_at	AB026997	CAST	0.017466642	0.000720642	0.001385527
1448259_at	BI452727	Fstl	0.017438819	0.000736828	0.001389968
1425463_at	BM214048	Gata6	0.017424457	0.000745315	0.001394409
1416693_at	NM_013519	Foxc2	0.017387499	0.000767574	0.00139885
1417837_at	NM_009434	Tssc3	0.017383794	0.000769839	0.001403291
1460285_at	NM_133721	Itga9	0.017360333	0.000784327	0.001407731
1422833_at	NM_010446	Foxa2	0.017295445	0.000825726	0.001412172
1454995_at	AW556888	AW050362	0.017233145	0.000867373	0.001416613
1429310_at	BE945486	5530600M07Rik	0.017233144	0.000867374	0.001421054
1426241_a_at	AB030906	scmh1	0.017228223	0.000870745	0.001425495
1434129_s_at	BG917242	AI447312	0.017225372	0.000872704	0.001429935
1437886_at	BM247104	Mm.86699.1	0.017212626	0.000881511	0.001434376
1434325_x_at	BB274009	Prkar1b	0.017204386	0.000887248	0.001438817
1417962_s_at	NM_010284	Ghr	0.017187676	0.00089899	0.001443258
1418189_s_at	AF146523	Ramp2	0.017173343	0.000909176	0.001447699
1441137_at	AV260198	Mm.179033.1	0.017161839	0.000917428	0.001452139
1438840_x_at	AI527359	Apoa1	0.017131974	0.000939177	0.00145658
1452352_at	BG064656	Ctla2b	0.016996526	0.001043946	0.001461021
1438325_at	AI647591	Mm.35515.1	0.0169955	0.001044779	0.001465462
1418269_at	NM_013586	Loxl3	0.016976235	0.001060541	0.001469903
1454830_at	AV010392	Mm.29576.1	0.016974621	0.001061872	0.001474343
1449559_at	NM_013601	Msx2	0.016958414	0.001075318	0.001478784
1416761_at	BC014753	Hsd11b2	0.016935516	0.001094584	0.001483225
1448029_at	AA543734	D5Ertd189e	0.01692248	0.001105694	0.001487666
1436905_x_at	BB218107	Laptm5	0.016906476	0.001119476	0.001492106
1422889_at	BM218630	Pcdh18	0.016887285	0.001136212	0.001496547
1417272_at	NM_026667	9130005N14Rik	0.016857882	0.001162303	0.001500988
1419233_x_at	NM_009692	Apoa1	0.016847769	0.001171405	0.001505429
1418673_at	NM_011415	Snai2	0.016847768	0.001171405	0.00150987
1460351_at	BC021916	S100a11	0.016824971	0.001192164	0.00151431
1416904_at	NM_020007	Mbnl	0.016798301	0.001216879	0.001518751
1438020_at	BB036951	BB099155	0.016757883	0.001255239	0.001523192
1438672_at	BI134721	AI595373	0.016747515	0.001265257	0.001527633
1455626_at	AA987181	Hoxa9	0.016740451	0.001272125	0.001532074
1435639_at	BF580962	2610528A11Rik	0.016738429	0.001274098	0.001536514
1449522_at	NM_009472	Unc5h3	0.016702823	0.001309293	0.001540955
1451112_s_at	BC024876	4921531N22Rik	0.016676449	0.001335939	0.001545396
1452378_at	AW012617	Mm.220932.1	0.016655191	0.001357779	0.001549837
1448694_at	NM_010591	Jun	0.016642009	0.001371487	0.001554278
1433977_at	BG918344	AW536289	0.016632515	0.001381438	0.001558718
1437082_at	BB246410	C79026	0.016525157	0.0014987	0.001563159
1434797_at	BB054275	Mm.89552.1	0.01647659	0.001554703	0.0015676
