# Supplementary material for: Estimating the Quality of Reprogrammed Cells Using ES Cell Differentiation Expression Patterns
Source: PLoS One. 2011 Jan 11;6(1):e15336. doi: 10.1371/journal.pone.0015336 (PMC3023460; doi:10.1371/journal.pone.0015336)
Supplement: Table S21 — Significant Up-regulated Common Genes in GSE8884 and GSE9940. (PDF) [file pone.0015336.s024.pdf]

Table S21 Significant Up-regulated Common Genes in GSE8884 and GSE9940

Significant Up-regulated Common Genes

Transcriptional regulation related And Signaling		***Subcellsular Location***
MEIS1	Transcriptional regulator of PAX6	Nucleus
MEIS2	Belongs to the TALE/MEIS homeobox family	Nucleus
PLAGL1	Transcriptional regulator of the type 1 receptor for pituitary adenylate cyclase-activating polypeptide	Nucleus
ZBTB16	May play a role in myeloid maturation and in the development and/or maintenance of other differentiated tissues	Nucleus
ZNF503	May function as a transcriptional repressor	Nucleus
MYOZ2	Plays an important role in the modulation of calcineurin signaling	Cytoplasm
GUCY1A3	cGMP biosynthesis, GTP cyclase, generate the second messenger cGMP	Cytoplasm
DLK1	Neuroendocrine differentiation	Membrane
THBD	Specific endothelial cell receptor	Membrane
SPON1	Promotes the attachment of spinal cord and sensory neuron cells and the outgrowth of neurites	Extracellsular matrix
VTN	Growth hormone-dependent serum factor with protease-inhibiting activity	Extracellsular matrix
TTR	Thyroid hormone-binding protein	Extracellsular matrix
IGF2	Insulin-like growth factor 2	Extracellsular matrix
TBC1D9	May act as a GTPase-activating protein for Rab family protein(s).	-

Other Protein		***Subcellsular Location***
ALDH1A1	Retinoic acid synthesis	Cytoplasm
NCAM1	Involved in neuron-neuron adhesion, neurite fasciculation, outgrowth of neurites	Membrane
PRTG	May play a role in anteroposterior axis elongation	Membrane
COL11A1	Fibrillogenesis regulator	Extracellsular matrix
ADAMTS9	Glycans Cleaves and metalloproteinase, control of organ shape during development	Extracellsular matrix
RELN	Plays a role in layering of neurons in the cerebral cortex and cerebellum	Extracellsular matrix
LUM	Binds to laminin	Extracellsular matrix
CXCL14	CXC chemokine, down regulated in cancer cells	Extracellsular matrix
SST	Somatostatin inhibits the release of somatotropin.	Extracellsular matrix
SLIT2	Act as molecular guidance cue in cellular migration	Extracellsular matrix
SERPINA1	Inhibitor of serine proteases	Extracellsular matrix
COL3A1	Collagen type III	Extracellsular matrix
MMRN1	Adhesive protein and Carrier protein of platelet factor V/Va	Extracellsular matrix
