# Supplementary material for: Estimating the Quality of Reprogrammed Cells Using ES Cell Differentiation Expression Patterns
Source: PLoS One. 2011 Jan 11;6(1):e15336. doi: 10.1371/journal.pone.0015336 (PMC3023460; doi:10.1371/journal.pone.0015336)
Supplement: Table S22 — Significant Down-regulated Common Genes in GSE8884 and GSE9940. (PDF) [file pone.0015336.s025.pdf]

Table S22 Significant Down-regulated Common Genes in GSE8884 and GSE9940

Significant Down-regulated Common Genes

Transcriptional regulation related And Signaling		***Subcellsular Location***
NANOG	Development regulator	Nucleus
POU5F1	Development regulator	Nucleus
FOXH1	Transcriptional activator	Nucleus
POLR3G	Component of RNA polymerase III	Nucleus
SOHLH2	Transcription factor may be involved in spermatogenesis and oogenesis	Nucleus
ZFP42	Transcriptional regulator in self-renew of ES cells	Nucleus
ZSCAN10	Transcriptional regulator in self-renew of ES cells	Nucleus
RBM35A	mRNA splicing factor that regulates the formation of epithelial cell-specific isoforms	Nucleus
DNMT3B	Genome wide de novo methylation during development	Nucleus
LARP7	Negative transcriptional regulator of polymerase II genes	Nucleus
TDGF1	Determination of the epiblastic cells	Cells membrane
EPHA1	Inhibition of antigen receptor-induced apoptosis	Cells membrane
LCK	T-cells development	Cells membrane
IFITM1	Implicated in the control of cell growth	Cells membrane
CXCL5	Neutrophil activation	Extracellsular matrix
DMKN	Regulator of keratinocyte differentiation	Extracellsular matrix
LEFTY1	Developmental axis regulator	Extracellsular matrix
KLKB1	Factor XII activation, cleaves Lys-Arg and Arg-Ser bonds	Extracellsular matrix
LGALS1	May regulate apoptosis, cell proliferation and cell differentiation	Extracellsular matrix
PMAIP1	Promotes activation of caspases and apoptosis	Mitochondria

Other Protein		***Subcellsular Location***
SCNN1A	Sodium ion channel	Cells membrane
PPAP2C	Phosphatidic acid metabolism	Cells membrane
CYP26A1	Retinoic acid metabolism	ER membrane
CYP2S1	Extrahepatic xenobiotic metabolism	ER membrane
GAL	Protein glycans modification	Golgi apparatus
TIMP4	Metalloproteinases inactivator	Extracellsular matrix
SCGB3A2	Secretoglobin	Extracellsular matrix
TERF1	negatively regulates telomere length	Telosome
HESRG	Marker of undifferentiated ES cells	-
L1TD1	LINE-1 type transposase domain-containing protein 1/ ES cell-associated protein 11	-
MT1H	Metallothioneins	-
MT1X	Metallothioneins	
CDA	UMP synthesis	-
