# Supplementary material for: Estimating the Quality of Reprogrammed Cells Using ES Cell Differentiation Expression Patterns
Source: PLoS One. 2011 Jan 11;6(1):e15336. doi: 10.1371/journal.pone.0015336 (PMC3023460; doi:10.1371/journal.pone.0015336)
Supplement: Table S23 — Significant Up-regulated Common Genes in GSE10970 and GSE3653. (PDF) [file pone.0015336.s026.pdf]

Table S23 Significant Up-regulated Common Genes in GSE10970 and GSE3653

Significant Up-regulated Common Genes

Transcriptional regulation related And Signaling		***Subcellsular Location***
Foxc2	Transcriptional activator.	Nucleus
Hbb-b2	Hemoglobin subunit beta-2	Nucleus
Rgs5	Regulator of G-protein signaling 5	
Mef2c	Myocyte-specific enhancer factor 2C	Nucleus
Igf1	Insulin-like growth factor I	Secreted
Prdm6	Putative histone-lysine N-methyltransferase PRDM6	Nucleus
Pcsk5	Proprotein convertase subtilisin/kexin type 5	
Ednrb	Endothelin B receptor	Cell membrane
Klhl6	Kelch-like protein 6	
Hapln1	Hyaluronan and proteoglycan link protein 1	Secreted
Rasgrp3	Rasgrp3	
Unc5c	Netrin receptor UNC5C	Cell membrane
Ttr	Thyroid hormone-binding protein	Secreted
Pkdcc	rotein kinase which is required for longitudinal bone growth through regulation of chondrocyte differentiation	Golgi apparatus
9430010M06Rik		
A730054J21Rik		
