# Supplementary material for: Estimating the Quality of Reprogrammed Cells Using ES Cell Differentiation Expression Patterns
Source: PLoS One. 2011 Jan 11;6(1):e15336. doi: 10.1371/journal.pone.0015336 (PMC3023460; doi:10.1371/journal.pone.0015336)
Supplement: Table S24 — Significant Down-regulated Common Genes in GSE10970 and GSE3653. (PDF) [file pone.0015336.s027.pdf]

Table S24 Significant Down-regulated Common Genes in GSE10970 and GSE3653

Significant Down-regulated Common Genes		
Transcriptional regulation related And Signaling		***Subcellsular Location***
Pou5f1	Transcription factor that binds to the octamer motif (5'-ATTTGCAT-3')	Nucleus
BB663928		
