# Supplementary material for: Estimating the Quality of Reprogrammed Cells Using ES Cell Differentiation Expression Patterns
Source: PLoS One. 2011 Jan 11;6(1):e15336. doi: 10.1371/journal.pone.0015336 (PMC3023460; doi:10.1371/journal.pone.0015336)
Supplement: Table S25 — Noise Random permutation testing to developing line (GSE13149): The Projection location of Dataset GSE6998. (PDF) [file pone.0015336.s028.pdf]

Table S25 Noise Random permutation testing to developing line(GSE13149): The Projection location of Dataset GSE6998

Percentage of replaced genes in developing lines(GSE13149)	Projetion location of Dataset GSE6998				
	10.5D	11.5D	12.5D	13.5D	14.5D
No noise control of GSE13149	-37.3966	-19.6526	1.357495	11.11046	21.20649
1% (450 genes)	-36.9312	-18.8901	0.270291	10.9965	20.8572
2% (900 genes)	-25.9895	-13.4367	-0.48816	8.466691	15.11989
3% (1350 genes)	-20.0673	-11.1809	0.249739	6.620223	12.21028
4% (1800 genes)	-14.8412	-9.27313	0.225472	5.085116	10.01312
5% (2250 genes)	-12.9454	-7.99962	1.078722	4.285212	8.123463
6% (2700 genes)	-10.351	-7.03866	1.001951	4.010671	6.664419
7% (3150 genes)	-8.34155	-6.15171	0.900379	3.045977	6.315705
8% (3600 genes)	-8.28387	-4.91383	1.059235	3.035469	5.80551
9% (4050 genes)	-8.0561	-4.43786	0.728134	3.048114	5.112041
10% (4500 genes)	-7.25408	-3.38691	1.358144	2.682438	3.532475
11% (4950 genes)	-6.08592	-2.86441	0.544886	2.894816	2.946832
12% (5400 genes)	-5.05158	-3.32822	0.645468	2.39346	3.39673
13% (5850 genes)	-4.74451	-2.99009	0.507514	2.099745	3.421113
14% (6300 genes)	-3.92935	-2.831	-0.27865	1.554619	3.883645
15% (6750 genes)	-3.263	-2.25544	-0.63148	1.766049	2.857827
16% (7200 genes)	-3.0765	-2.2089	-0.26018	1.443825	2.525469
17% (7650 genes)	-2.37174	-2.81527	-0.61043	2.143308	2.242622
18% (8100 genes)	**-1.91177**	**-2.53789**	**-0.25003**	**2.02181**	**1.653655**
19% (8550 genes)	**-1.20862**	**-1.34734**	**-0.53392**	**1.768662**	**1.117046**
20% (9000 genes)	**-1.2182**	**-0.97505**	**-0.55767**	**1.958003**	**0.725964**
